# Efficacy and safety-in analysis of short-course radiation followed by mFOLFOX-6 plus avelumab for locally advanced rectal adenocarcinoma

**DOI:** 10.1186/s13014-020-01673-6

**Published:** 2020-10-07

**Authors:** Ali Shamseddine, Youssef H. Zeidan, Ziad El Husseini, Malek Kreidieh, Monita Al Darazi, Rim Turfa, Joseph Kattan, Ibrahim Khalifeh, Deborah Mukherji, Sally Temraz, Kholoud Alqasem, Rula Amarin, Tala Al Awabdeh, Samer Deeba, Faek Jamali, Issa Mohamad, Mousa Elkhaldi, Faiez Daoud, Mahmoud Al Masri, Ali Dabous, Ahmad Hushki, Omar Jaber, Maya Charafeddine, Fady Geara

**Affiliations:** 1 Division of Hematology/Oncology, Department of Internal Medicine, Naef K. Basile Cancer Institute - NKBCI, American University of Beirut Medical Center, Beirut, Lebanon; 2 Department of Radiation Oncology, American University of Beirut Medical Center, Beirut, Lebanon; 3 Division of Hematology/Oncology, Department of Internal Medicine, King Hussein Cancer Center, Amman, Jordan; 4 Department of Hematology/Oncology, Hotel-Dieu de France University Hospital, Beirut, Lebanon; 5 Department of Pathology and Laboratory Medicine, American University of Beirut Medical Center, Beirut, Lebanon; 6 Division of General Surgery, Department of Surgery, American University of Beirut Medical Center, Beirut, Lebanon; 7 Department of Radiation Oncology, King Hussein Cancer Center, Amman, Jordan; 8 Department of Surgical Oncology, King Hussein Cancer Center, Amman, Jordan; 9 Division of Gastroenterology, Department of Internal Medicine, King Hussein Cancer Center, Amman, Jordan; 10 Department of Pathology, King Hussein Cancer Center, Amman, Jordan

**Keywords:** Rectal cancer, Radiotherapy, Chemotherapy, Immunotherapy, Neo-adjuvant therapy

## Abstract

**Background:**

Neoadjuvant chemotherapy and short-course radiotherapy followed by resection has been gaining recognition in the treatment of rectal cancer. Avelumab is a fully human immunoglobulin that binds Programmed Death-Ligand 1 (PD-L1) and prevents the suppression of the cytotoxic T cell immune response. This phase II trial evaluates the safety and pathologic response rate of short-course radiation followed by 6 cycles of mFOLFOX6 with avelumab in patients with locally advanced rectal cancer (LARC).

**Methods:**

This study is prospective single-arm, multicenter phase II trial adopting Simon’s two-stage. Short-course radiation is given over 5 fractions to a total dose of 25 Gy. mFOLFOX6 plus avelumab (10 mg/kg) are given every 2 weeks for 6 cycles. Total mesorectal excision is performed 3–4 weeks after the last cycle of avelumab. Follow up after surgery is done every 3 months to a total of 36 months. Adverse event data collection is recorded at every visit.

**Results:**

13 out of 44 patients with LARC were enrolled in the first stage of the study (30% from total sample size). All patients met the inclusion criteria and received the full short-course radiation course followed by 6 cycles of mFOLFOX6 plus avelumab. 12 out of the 13 patients completed TME while one patient had progression of disease and was dropped out of the study. The sample consisted of 9 (69%) males and 4 (31%) females with median age of 62 (33–73) years. The first interim analysis revealed that 3 (25%) patients achieved pathologic complete response (pCR) (tumor regression grade, TRG 0) out of 12. While 3 (25%) patients had near pCR with TRG 1. In total, 6 out of 12 patients (50%) had a major pathologic response. All patients were found to be MMR proficient. The protocol regimen was well tolerated with no serious adverse events of grade 4 reported.

**Conclusion:**

In patients with LARC, neoadjuvant radiation followed by mFOLFOX6 with avelumab is safe with a promising pathologic response rate.

*Trial Registration Number and Date of Registration* ClinicalTrials.gov NCT03503630, April 20, 2018. https://clinicaltrials.gov/ct2/show/NCT03503630?term=NCT03503630&draw=2&rank=1.

## Background

Colorectal cancer currently ranks third in diagnosis following lung and female breast cancers [1, 2]. The treatment for the locally advanced rectal cancer has a trimodal approach consisting of neoadjuvant chemoradiotherapy followed by total mesorectal excision (TME) and adjuvant chemotherapy. In an effort to improve compliance to treatment and toxicity, chemotherapy is delivered before surgery without compromising response rates [3]. For this reason, the NCCN guidelines accepted total neoadjuvant treatment for locally advanced rectal cancer [4]. A recent phase II trial concluded that giving up to 6 cycles of mFOLFOX6, after chemoradiation and before TME, leads to an increase in pathologic complete response rates [5]. Multiple studies indicate that tumor response to preoperative treatment strongly predicts the disease-free survival of patients [6].

The DNA mismatch repair (MMR) proteins serve to decrease DNA damage. When deficient (dMMR), mutations accumulate leading to carcinogenesis. dMMR patients are associated with poorer response to adjuvant chemotherapy in colon cancer while showing a significant response to immune checkpoint inhibitors [7]. A phase II clinical trial showed that dMMR cases, irrespective of the primary tumor, were more responsive to pembrolizumab, an anti-PD-1 antibody, than MMR proficient (pMMR) cases [8]. Additional evidence suggests that radiotherapy can act as an in-situ vaccine, with well-documented immunogenic response leading in some cases to distant effects also known as, abscopal effect. Preclinical data showed that fractionation, and not single dose radiotherapy, synergizes with immunotherapy to induce the abscopal effect [9]. Moreover, radiation augments antigen presentation in tumor cells, increases T lymphocytes infiltration and expands the T cell receptor repertoire [10]. Demaria et al. showed that the combination of radiotherapy with anti-cytotoxic T-lymphocyte-associated protein 4 (CTLA-4) led to an improvement in the overall survival in mice [11].

On the other hand, the programmed death-ligand 1 (PD-L1) is used by tumor cells to avoid the adaptive immune response [12]. Where fractionated radiotherapy was found to induce the upregulation of PD-L1 on tumor cells and the combination of anti-PD-L1 with radiotherapy led to improvement in local control and survival [13].

Taking this into consideration, the current phase II trial combines short-course radiation with avelumab, an anti-PD-L1, and chemotherapy (mFOLFOX6) in order to evaluate the safety and efficacy of this combination in patients with LARC.

## Methods

### Eligibility criteria

Patients were enrolled according to the following inclusion criteria: patients aged ≥ 18 years, had locally advanced rectal cancer with a biopsy documenting rectal adenocarcinoma (cT2 N1-3, cT3 N0-3 or cT4a N0-3), distance from anal verge was < 15 cm, an Eastern Cooperative Oncology Group (ECOG) performance score of ≤ 1 and with adequate organ function. Patients were excluded if they presented with distant metastasis, clinical stage of T2N0 or T4b, or recurrent rectal cancer.

### Study design and endpoints

**Fig. 1:**
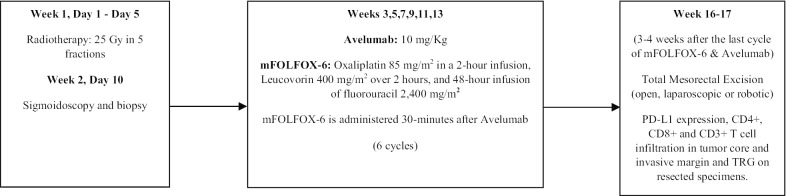
Treatment protocol

The overall study protocol is provided in Fig. 1. During the first week, patients underwent short course radiation therapy. Prior to radiotherapy patients underwent 3 dimensional CT simulation. Intensity modulated radiotherapy and 3 D conformal treatment were allowed for treatment delivery. Treatment volumes were defined as follows: GTV includes all primary tumor extent, and enlarged lymph nodes, CTV includes GTV with 0.5 cm extension and all perirectal, presacral, and internal iliac lymph nodes all the way up to the sacral promontory, PTV is a 1 cm expansion of the CTV in all directions. Prescription dose was 25 Gy (prescribed to PTV) delivered over 5 fractions. After one week, patients received the first cycle of mFOLFOX6 (oxaliplatin 85 mg/m^2^ in a 2-h infusion, leucovorin 400 mg/m^2^ over 2 h and 48-h infusion of fluorouracil 2400 mg/m^**2**^) 30 min after avelumab (10 mg/Kg). The same regimen was repeated every 2 weeks for 6 cycles. Then 3–4 weeks after the last cycle, patients underwent total mesorectal excision.

Primary endpoint was to evaluate the extent of pathologic response. Secondary endpoints were to assess the safety and tolerability of mFOLFOX6 with avelumab after short-course radiation, and correlate PD-L1 expression, CD3, CD4 and CD8 positive T lymphocytes infiltration (before and after treatment) and tumor regression grade with clinical outcomes (Additional file 1: Protocol).

### Follow-up evaluation

Patients will follow up after surgery every 3 months for a total of 36 months. At each follow up visit, vital signs, tumor markers, CEA and CA 19–9, are ordered and adverse events are documented according to the National Cancer Institute Common Terminology Criteria for Adverse Events version 3. Patients undergo chest, abdomen and pelvis CT scan every 6 months and colonoscopy yearly after surgery.

### Statistical analysis

The trial follows a Simon’s two-stage design with a null hypothesis pCR rate ≤ 16% versus the alternative that pCR rate ≥ 35%, a type I error of 0.05 and a power of 80%. The use of the Simon 2-stage design enables an interim analysis for both efficacy and safety to be performed following treatment of the eligible patients As per the Simon two-stage calculation, the optimal stage design for the first stage was 13 patients with probability of early stopping of 0.6537 [14]. The optimal stage design, which achieves reductions in expected sample size, was preferred to minimize the number of patients exposed to inactive treatment. The duration of this study depends on the results of the interim analysis, i.e., the probability of early termination, if only 2 or fewer patients achieve pCR at stage one. The results of the first 13 patients were assessed in the first preliminary stage. More than 2 patients with pCR were needed to continue into second stage. A total of 44 patients will be enrolled in the second stage. If overall 10 or more patients achieve pCR, then the null hypothesis will be rejected.

### Ethical approval

The study was approved by the Institutional Review Board at the American University of Beirut with 11 voting members and registered in an international public registry.

## Results

### Patients’ characteristics

A total of 13 patients were enrolled with 9 (69.2%) males and 4 (30.8%) females (Table 1). Median age was 62 years ranging between 33 and 74 years. Accrual was from 3 centers, 2 in Lebanon and 1 in Jordan, 8 participants (61.5%) were Lebanese, 2 (15.4%) were Iraqi and 3 (23.1%) were Jordanian. All patients had an ECOG score of ≤ 1. None of the patients were found to have a deficient MMR profile. Pathology review showed that 10 (76.9%) had intestinal histology type, 1 (7.7%) mucinous, 1 (7.7%) combined mucinous and intestinal and 1 (7.7%) combined signet ring cell and intestinal. 2 (15.3%) patients had a well differentiated tumor, 10 (77%) moderately differentiated and 1 (7.7%) had a poorly differentiated tumor. The median distance from the anal verge was 10 cm, ranging between 3 and 14 cm. Clinical stage at diagnosis was distributed as 1 (7.7%) cT3N0, 4 (30.8%) cT3N1, 7 (53.8%) cT3N2 and 1 (7.7%) cTxN1 (under radiologic review). Baseline MRI results showed 3 out of 6 (50%) with positive CRM and 1 out of 6 (16.7%) with positive EMVI. Patient characteristics are shown in Table 1.

**Table 1:** Patients’ characteristics

Demographics, n	13
Median age, years (range)	62.2 (33.7–74.0)
Sex, n (%)	
Male	9 (69.2)
Female	4 (30.8)
Nationality, n (%)	
Lebanese	8 (61.5)
Iraqi	2 (15.4)
Jordanian	3 (23.1)
ECOG performance status, n (%)	
≤ 1	13 (100)
> 1	0 (0)
MMR mutational status, n (%)	
MSS	13 (100)
MSI-H	0 (0)
Tumor histology type, n (%)	
Intestinal	10 (76.9)
Mucinous	1 (7.7)
Combined Mucinous and Intestinal	1 (7.7)
Combined Signet ring cell and Intestinal	1 (7.7)
Tumor differentiation, n (%)	
Well	2 (15.3)
Moderate	10 (77)
Poorly	1 (7.7)
Median distance from anal verge, cm (range)	10 (3–14)
Clinical stage, n (%)	
TxN1	1 (7.7)
T3N0	1 (7.7)
T3N1	4 (30.8)
T3N2	7 (53.8%)

ECOG, Eastern Cooperative Group Oncology Status; MMR, mismatch repair; MSI-H, microsatellite instability-high; MSS, microsatellite-stable

### Response rate

**Fig. 2:**
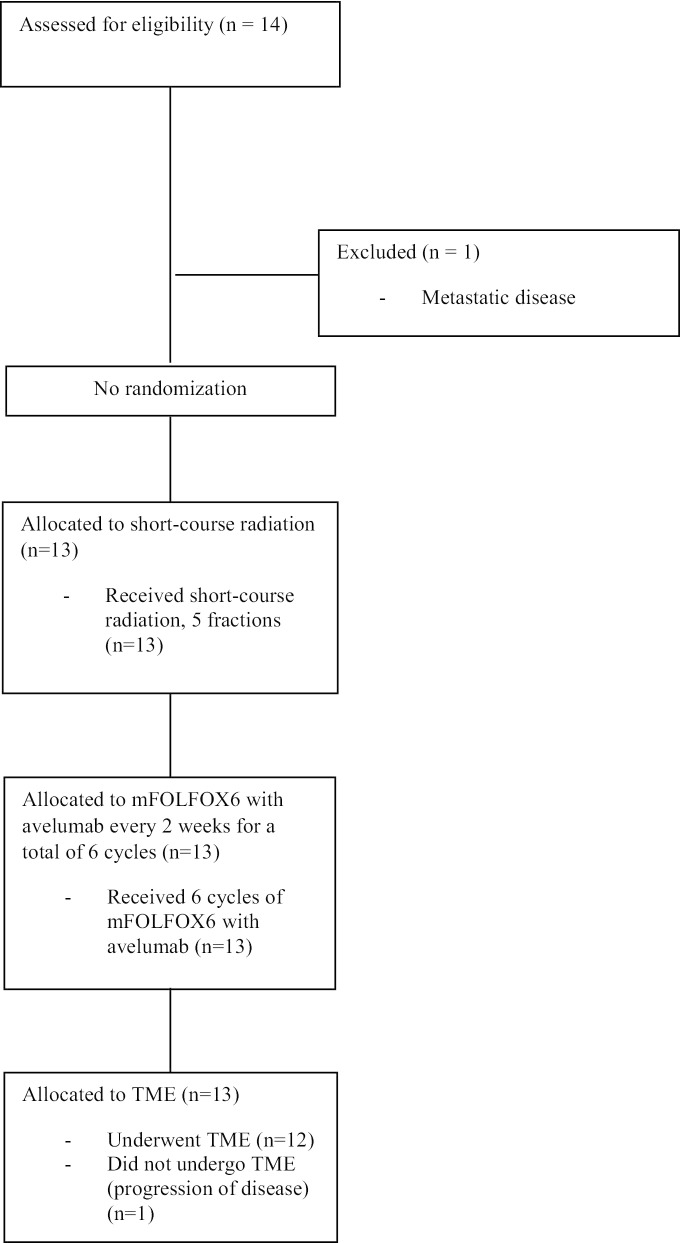
Consort diagram

Of the 13 patients, 12 underwent total mesorectal excision. 1 patient progressed after treatment with 6 cycles of avelumab and mFOLFOX and had to be dropped out of the study before undergoing TME (Fig. 2). 3 (25%) patients had pathologic complete response (TRG 0), 3 (25%) had < 10% viable tumor cells (TRG 1), 4 (33.3%) had 10–50% viable tumor cells (TRG 2) and 2 (16.7%) had > 50% viable tumor cells (TRG 3). As for the pathologic staging following surgery, 3 (25%) patients had ypT0N0, 1 (8.3%) patient ypT2N0, 4 (33.3%) patients ypT3N0, 2 (16.7%) patients ypT3N1, 1 (8.3) patient ypT3N2 and 1 (8.3%) patient ypT4aN2. The pathologic response data is re-demonstrated in Table 2.

**Table 2:** Response rate

Pathology specimens, n	12
Pathologic responses, n (%)	
Complete pathologic response (TRG 0)	3 (25)
Partial pathologic response	
< 10% viable cells (TRG 1)	3 (25)
10–50% viable cells (TRG 2)	4 (33.3)
> 50% viable cells (TRG 3)	2 (16.7)
Pathologic staging, n (%)	
ypT0N0	3 (25)
ypT2N0	1 (8.3)
ypT3N0	4 (33.3)
ypT3N1	2 (16.7)
ypT3N2	1 (8.3)
ypT4aN2a	1 (8.3)

### Quality of TME surgery

The complete resection of mesorectum in TME is assessed based on the criteria of the college of American Pathologists (CAP, 2013). Indeed, the specimens are macroscopically assessed and graded as: complete (mesorectum is intact and smooth and any defect in surface is not deeper than 5 mm, CRM is smooth and regular), nearly complete (the mesorectum is irregular however no muscularis propria is visible, CRM is irregular) and incomplete (the mesorectum is little bulk with defects reaching muscularis propria, CRM is irregular). 6 images of the 12 specimens were taken. All the 6 assessed specimen were grade as complete (100%); However, the grading of the rest of the specimens are not available due to the lack of the gross specimen photos.

### Safety

A total of 3 adverse events, grade 3 (severe) were documented. One case of small intestinal obstruction, one case of salmonella colitis and one case of acute kidney injury (AKI). A total of 27 adverse events graded less than 3 were documented, with 36% of the cases being diarrhea and fatigue. None of the adverse events were secondary to avelumab use. Only the AKI was related to the TME surgery. In brief, 10 days post TME, the patient presented to the clinic with diarrhea. sweating and dehydration. Patient was hospitalized for 3 days and treated with hypertonic intravenous hydration. Patient was discharged with no sequalae. Adverse events are shown in Table 3.

**Table 3:** Adverse events

Adverse events	Grade < 3, n (%)	Grades 3 or 4, n (%)
White blood cells decreased	1 (3.3)	0
Hypotension	1 (3.3)	0
Diarrhea	6 (20)	0
Anorexia	1 (3.3)	0
Nausea	2 (6.7)	0
Abdominal distention	2 (6.7)	0
Abdominal pain	1 (3.3)	0
Anal pain	1 (3.3)	0
Constipation	1 (3.3)	0
Hemorrhoids	1 (3.3)	0
Vomiting	2 (6.7)	0
Chills	2 (6.7)	0
Fatigue	5 (16.7)	0
Fever	1 (3.3)	0
Gram negative Bacilli	1 (3.3)	0
UTI	1 (3.3)	0
Localized shoulder edema	1 (3.3)	0
Insomnia	2 (6.7)	0
Dizziness	1 (3.3)	0
Vaginal discharge	1 (3.3)	0
Vulvar infection-herpes	1 (3.3)	0
Upper respiratory tract infection	2 (6.7)	0
Cough	1 (3.3)	0
Dry skin	3 (10)	0
Rash	2 (6.7)	0
Palmar-plantar erythrodysesthesia syndrome	1 (3.3)	0
Skin irritation	1 (3.3)	0
Small intestinal obstruction	0	1 (3.3)
Colitis	0	1 (3.3)
Acute kidney injury	0	1 (3.3)

## Discussion

To our knowledge, this is the first phase II trial studying the efficacy and safety of avelumab with mFOLFOX6 after short-course radiotherapy. The total neoadjuvant approach has been receiving increasing attention lately in the treatment of locally advanced rectal cancer. This treatment approach is supported by better compliance rates, fewer toxicity profiles, and better pathologic complete response rates [15]. In the first stage of this trial, we reached a 25% pathologic complete response rate, which is higher than historic preoperative standard [16]. In comparison, a phase II trial studying the combination of long course chemoradiation followed by 6 cycles of FOLFOX prior to TME reached a pCR rate of 37% [5].

None of the 13 enrolled patients were MMR deficient which could have impacted our pCR rates. Prior studies showed a clinical benefit in MMR deficient patients treated with anti-PD-1 in comparison to MMR proficient patients [10]. This is secondary to the cytotoxic T cell infiltration associated with MMR deficient cases that expands with the blockade of PD-1 or PD-L1. For this reason, efforts are made to induce T cell activation in MMR proficient patients through combining immunotherapy with radiotherapy and chemotherapy. Radiotherapy is well known and used for its direct cytotoxic effects, but recently more studies are exploring its immunogenic effects. In a study comparing short-course radiation, 25 Gy in 5 fractions, with long-course chemoradiation, 50.4 Gy in 28 fractions, it was demonstrated that only with short-course radiation the cytotoxic T cells increased inside the tumor [17]. Hence, higher dose per fraction may result in differential immunogenic reaction. Currently, the RAPIDO trial is comparing preoperative long-course radiation with short-course radiation in a two-arm prospective randomized trial to test the hypothesis of that short-course radiation with neoadjuvant chemotherapy increases disease-free and overall survival [18]. Other clinical trials are currently studying the effects of immunotherapy on metastatic and deficient mismatch repair colorectal cancer [19]. This trial is the first to assess the efficacy of an immune checkpoint inhibitor post short course radiation with chemotherapy in LARC. Our results indicate that the avelumab with mFOLFOX6 after short-course radiation is well tolerated with promising efficacy.

One of the main controversies in the treatment of LARC is the “watch and wait” approach. Many are championing for skipping surgery and following up with imaging after a clinical complete response. But this approach is limited by the post-radiation effects of inflammation and fibrosis that can be mistaken for tumor on imaging [20]. Alternatively, in a prospective study for patients with clinical complete response, only 1 patient out of 21 developed local recurrence [21]. This result was reached after strict MRI and colonoscopy follow ups. Such an approach needs to be carefully studied in a clinical trial protocol. The OPRA trial is currently evaluating in a randomized trial the outcome in patients treated with chemoradiation and neoadjuvant chemotherapy with either TME or watch-and-wait policy [22]. Further studies will help clarify which patient population is ideal for such an approach.

Finally, our study has several limitations that should be discussed. Initially, since this is a phase II nonrandomized clinical trial, the pathologic complete response rate reached could be secondary to the adoption of total neoadjuvant treatment without the added effect of an immune checkpoint inhibitor. A randomized trial is needed to prove the efficacy and superiority of adding an immune checkpoint inhibitor to the treatment of LARC. Secondly, this is a report of the results obtained in the first stage of this trial, where only 13 patients were enrolled. A more comprehensive report in the future should include all 44 patients planned to be enrolled. Additionally, the follow up period is still too short to analyze the disease-free and overall survival results. Nonetheless, the results achieved are enough to pursue this treatment combination in this phase II trial and could allude to future phase III trials.

## Conclusion

As more studies prove the superiority of total neoadjuvant therapy in comparison to standard therapy, guidelines will shift into maximizing treatment before surgery for better compliance, toxicity profiles, and pathologic complete response rates. Combining radiotherapy with immunotherapy and chemotherapy is a promising approach in patients with LARC. Interim results from our ongoing phase II trial show that short-course radiation, followed by mFOLFOX6 with avelumab is well tolerated with an encouraging pathologic response rate.

## Supplementary information

**Additional file1:** Protocol. Protocol of Averectal study.

## Data Availability

Research data are not available at this time.
