# Supplementary material for: Efficacy and safety-in analysis of short-course radiation followed by mFOLFOX-6 plus avelumab for locally advanced rectal adenocarcinoma
Source: Radiat Oncol. 2020 Oct 7;15:233. doi: 10.1186/s13014-020-01673-6 (PMC7542723; doi:10.1186/s13014-020-01673-6)
Supplement: Supplementary file 1 — Additional file1: Protocol. Protocol of Averectal study. [file 13014_2020_1673_MOESM1_ESM.doc]

**SHORT-COURSE RADIATION FOLLOWED BY MFOLFOX-6 PLUS AVELUMAB FOR LOCALLY-ADVANCED RECTAL ADENOCARCINOMA**

**CLINICAL STUDY PROTOCOL - BIO-2017-0422**

**Funder ID number: MS100070_0021 ISS**

**SPONSOR** Investigator Sponsored Study

American University of Beirut

Beirut PO Box 11-0236, Lebanon

**PRINCIPAL INVESTIGATOR** Dr Ali Shamseddine, MD, FRCP

**AND MEDICAL CONTACT**  Department of Internal Medicine

Division of Hematology/Oncology

American University of Beirut Medical Center

Beirut P.O. Box 11-0236, Lebanon

Phone: +961 1 350 000 (Ext.: 5390)

Email: as04@aub.edu.lb

**CLINICAL RESEARCH**  Phoenix Clinical Research

**ORGANIZATION** Berytech Technology and Health

Damascus Road – Museum area

Beirut, Lebanon

+961 1 612 500 (Ext.: 3090)

Email: glabaki@phoenix-cr.com

**VERSION / RELEASE DATE** 2.3 including Amendment I / 04 October 2018

# OVERVIEW OF AMENDMENTS INTEGRATED IN THE PROTOCOL TEXT OF VERSION 1.0 OF 22 SEPTEMBER 2017

| **Amendment No.** | **Protocol version** | | **Date** | | **Sections concerned** | **Description of modifications** |
| --- | --- | --- | --- | --- | --- | --- |
| Creation of the document | 1.0 | | 22 September 2017 | | - | - |
| I | | 2.0 | | 22 February 2018 | Protocol number/code | - Change of the protocol number/code from IM.AS.36 to BIO-2017-0422. |
|  |  |  |  |  | Table 1 | - TSH, free T4 and pregnancy test were removed during the follow-up phase and last visit. - TSH, free T4 and pregnancy test are to be done on visit 1 (inclusion visit), visit 10 (cycle 3 of chemotherapy and week 7) and visit 14 (surgery and week 16/17) only. - Other scheduled thyroid tests were removed (weeks 8, 12, 13, follow-up visits and end of study visit). - Visit time window to be +/- 3 days. |
|  |  |  |  |  | Section 3.5 | - Deletion of the following information: No formal Data and Safety Monitoring Board (DSMB) will be set up. However, the Coordination Investigator and the co-Principal Investigators will review the safety and efficacy data at the end of the first stage of the study, to decide about proceeding with the second stage of the study. - To be replaced by: A Data and Safety Monitoring Board (DSMB) will be constituted to ensure patient safety … involvement in clinical research and investigation development methods. |
|  |  |  |  |  | Section 8.1 | - Deletion of the following information: If not possible, informed consent may be only signed by the investigator if obtained in an emergency procedure. The patient’s written consent will be requested as soon as the patient is capable of doing so. |
|  |  |  |  |  | Sections 8.2.6 and 8.2.7 | - TSH, free T4 and pregnancy test were removed during the follow-up phase and last visit. |
|  |  |  |  |  | Section 9.4 | - Deletion of the following information: In case of anaphylactic shock, standard medical treatment should be implemented. |
|  |  |  |  |  | Section 11.1.2.6 | - Addition of the following information: The CRAs or other agents of the sponsor shall not access the site’s medical records unaccompanied by the site’s medical staff. Also, no personal health information is recorded by the CRA except as authorized by the IRB approved protocol; the IRB approved signed subject consent form and the signed clinical trial agreement. |
|  |  |  |  |  | Section 17 | - Virology testing is moved from ‘standard of care’ to ‘Trial-specific procedures’ |
|  |  |  |  |  | Appendix 20.0 | - Addition of the name of Dr. Elie Chouillard as co-investigator. |
| I | | 2.1 | | 12 April 2018 | Section 3.5 | - Names of experts are included in the DSMB: Dr. Ahmad Awada and Dr. Alain Hendlisz. Both are gastrointestinal oncologists at Institut Jules Bordet, Brussels, Belgium. |
| I | | 2.2 | | 11 May 2018 | Section 3.5 | - Name of a new expert is included in the DSMB: Dr. Hani Tamim, PhD, Associate Professor of Medicine, and Director of the Biostatistics Unit at the Clinical Research Institute at the Faculty of Medicine, American University of Beirut. |
|  |  |  |  |  | Sections 9.5.4 and 9.6 | - Replacement of GlobalDrugSafety@merckgroup.com with [ICSR_GDS@merckgroup.com](mailto:ICSR_GDS@merckgroup.com) |
| I | | 2.3 | | 07 August 2018 | Table 1 | - PT and PTT should be assessed on visit 14 (a couple of days prior to surgery on week 16 or 17) rather than on visit 13 (week 13 ± 3 days). |
|  |  |  |  |  |  | - PD-L1 expression & CD4+, CD8+ and CD3+ T cell infiltration are determined on baseline biopsy during visit one. |
|  |  |  |  |  |  | - MSI or MMR status is determined once on either the baseline biopsy or D10 biopsy rather than on the D10 biopsy or surgical specimen. |
|  |  |  |  |  |  | - Addition of a section for avelumab premedication. |
|  |  |  |  |  | Synopsis and Section 3.1 | - Deletion of the following information that mFOLFOX-6 chemotherapy plus avelumab (10 mg/kg) will be given every 2 weeks simultaneously. - To be replaced by the following: mFOLFOX-6 chemotherapy plus avelumab (10 mg/kg) will be given every 2 weeks in a successive manner such that mFOLFOX is administered 30 minutes after avelumab has already been administered. |
|  |  |  |  |  | Figure 1 | - MSI or MMR status is determined once on either the baseline biopsy or D10 biopsy rather than on D10 biopsy or surgical specimen. |
|  |  |  |  |  | Section 3.2 | - The Arabic version 4 of 03 September 2014 of the FACT-C questionnaire is included in addition to the English version 4 of 16 November 2007 as a tool in quality of life assessment. |
|  |  |  |  |  | Section 3.5 | - Addition that a report will be sent to the DSMB members after the first stage of the trial is completed and 13 patients are accrued from all centers to decide about proceeding with the second stage. - Addition that an annual meeting among the DSMB members will be held thereafter. |
|  |  |  |  |  |  | - Name of a new expert is included in the DSMB: Dr Ghassan Abou-Alfa who is a gastrointestinal oncologist at Memorial Sloan Kettering Cancer Center. |
|  |  |  |  |  | Synopsis and Section 4 | - Replacement of 15 January 2018 with 20 July 2018 as the date corresponding to the expected first patient first visit. |
|  |  |  |  |  |  | - Replacement of 15 July 2019 with 02 May 2023 as the date corresponding to the expected last patient last visit. |
|  |  |  |  |  | Section 5.1 | - Replacement of September 2018 with April 2019 as the month at which the first stage of the trial is expected to be reached with 13 patients being accrued from all centers. |
|  |  |  |  |  | Synopsis and Section 5.2.1 | - Replacement of <12 cm from anal verge with <15 cm from anal verge as an inclusion criterion. |
|  |  |  |  |  | Synopsis and Section 5.3 | - Replacement of 15 January 2018 with 20 July 2018 as the expected date of the first-patient in. |
|  |  |  |  |  |  | - Replacement of 15 July 2019 with 20 January 2020 as the expected date of the last-patient in. |
|  |  |  |  |  |  | - Replacement of 27 October 2022 with 02 May 2023 as the expected date of the last-patient last visit. |
|  |  |  |  |  |  | - Replacement of 27 November 2022 with 02 June 2023 as the expected date of database lock. |
|  |  |  |  |  |  | - Replacement of 15 January 2023 with 20 July 2023 as the expected date of key statistics initiation. |
|  |  |  |  |  |  | - Replacement of 15 April 2023 with 20 October 2023 as the expected date of Clinical Study Report approval. |
|  |  |  |  |  | Section 6.4.2.1 | - Addition that the required dose of avelumab is based on the patient’s bodyweight on each visit as illustrated by Equation (1): Required dose (mg) = 10 (mg/kg) x Patient weight (kg). |
|  |  |  |  |  |  | - Addition that the volume of avelumab and 0.9% saline solution or 0.45% saline solution needed for the dilution can be calculated using the following equations:   - Equation (2): Volume of avelumab (mL) = (Required dose from 𝐄𝐪𝐮𝐚𝐭𝐢𝐨𝐧 𝟏 in mg) / (20 mg/mL)   - Equation (3): Volume of Normal Saline (mL) = 250 mL – Volume of avelumab (mL). |
|  |  |  |  |  | Section 6.4.2.2 | - Deletion of the following information that avelumab 10 mg/kg is administered as an intravenous infusion over 60 minutes every 2 weeks, simultaneously with mFOLFOX-6 chemotherapy to every patient. - To be replaced by the following: Avelumab 10 mg/kg is administered to every patient as an intravenous infusion over 60 minutes, followed 30 minutes later by mFOLFOX-6 chemotherapy every 2 weeks as per the institution’s standard of practice. |
|  |  |  |  |  | Section 8.2.1 | - PD-L1 expression & CD4+, CD8+ and CD3+ T cell infiltration are determined on baseline biopsy during visit one. |
|  |  |  |  |  | Section 8.2.2 | - Replacement of the statement that the use of IMRT is strongly encouraged with the statement that both techniques are approved, so either technique may be used. |
|  |  |  |  |  |  | - Replacement of the statement that patients must be lying prone on a belly board or similar devices for bowel exclusion with the statement that patients are to be placed in a position that best suits the technique used and ensures immobilization and displacement of normal tissues. |
|  |  |  |  |  |  | - MSI or MMR status is determined once on either the baseline biopsy or D10 biopsy rather than on the D10 biopsy or surgical specimen. - Addition that an image of the biopsy taken during sigmoidoscopy is to be provided. |
|  |  |  |  |  | Section 8.2.5 | - Removal of “minimally invasive” as all TME procedures, whether open or laparoscopic or robotic, are not minimally invasive. |
|  |  |  |  |  |  | - Addition that the videotapes corresponding to the TME procedures and the images of the resected specimens are to be provided. |
|  |  |  |  |  |  | - Removal of MSI as one of the items to be evaluated on the resected tumor specimen. |
|  |  |  |  |  | Sections 8.2.4 and 8.2.5 | - PT and PTT should be assessed on visit 14 (a couple of days prior to surgery on week 16 or 17) rather than on visit 13 (week 13 ± 3 days). |
|  |  |  |  |  | Section 8.2.6 | - Addition of “Disease status” as the seventh thing to be assessed every 3 months for 3 years after surgery. |
|  |  |  |  |  | Section 8.2.7 | - Addition of the “Disease status” as the seventh thing to be assessed 3 years after surgery at the last follow-up visit. |
|  |  |  |  |  | Section 8.3.2 | - Replacement of the statement that MSI will be evaluated “once on either the resected specimen or D10 biopsy if there is a pathological complete response” from the second row with the statement that it will be evaluated “once on either the baseline biopsy or D10 biopsy”. |
|  |  |  |  |  | Section 9.5.4 | - Replacement of '24 hours” with “24 working hours”. |
|  |  |  |  |  | Section 10.3 | - The Arabic version 4 of 03 September 2014 of the FACT-C questionnaire is included in addition to the English version 4 of 16 November 2007 as a tool in quality of life assessment. |
|  |  |  |  |  | Appendix 20.0 | - Addition of the name of Dr Assaad Soweid as one of the co-investigators. |
|  | |  |  | 17 August 2018 | Table 1 | - Replacement of “urine pregnancy test for women of childbearing potential must be performed at baseline and least every month during treatment” with “urine or serum pregnancy test for women of childbearing potential and who are sexually active must be performed at baseline and least every month during treatment”. |
|  |  |  |  |  | Synopsis and Section 5.2.1 | - Replacement of “negative serum or urine pregnancy test at screening for women of childbearing potential” with “negative serum or urine pregnancy test at screening for women of childbearing potential who are sexually active”. |
|  |  |  |  |  | Section 8.2.1 | - Replacement of “urine pregnancy test for women of childbearing potential” with “urine or serum pregnancy test for women of childbearing potential who are sexually active”. - Addition of the following: “PDL-1 expression will be classified as negative or positive or not applicable (categorical variable)”. - Addition of the following: “CD4+, CD8+ and CD3+ T cell infiltration will be quantified in mm^2^ in the most abundant tumor-infiltrating area in both, the stroma and the tumor, of the baseline biopsy (continuous variable)”. - Addition of the following: “after the recruitment of around 5 patients, a cut-off value will be agreed upon and the numerical values corresponding to T cell infiltration (continuous variable) will then be classified as low or high or not applicable (categorical variable)”. - Addition of the following: “Microsatellite instability (MSI or MMR status) will be evaluated once on either the baseline biopsy or D10 biopsy”. - Addition of the following: “the MSI predictive markers to be assessed are: MLH-1, MSH-2, MSH-6, and PMS-2”. - Replacement of “each patient must be identified by 5-digit number which is a combination of his/her 2-digit number and 3-digit subject number” with “each patient must be identified by 6-digit number which is a combination of his/her 3-digit number and 3-digit subject number”. |
|  |  |  |  |  | Section 8.2.2 | - Addition of the following: “PDL-1 expression will be classified as negative or positive or not applicable (categorical variable)”. - Addition of the following: “CD4+, CD8+ and CD3+ T cell infiltration will be quantified in mm^2^ in the most abundant tumor-infiltrating area in both, the stroma and the tumor, of the D10 biopsy (continuous variable)”. - Addition of the following: “after the recruitment of around 5 patients, a cut-off value will be agreed upon and the numerical values corresponding to T cell infiltration (continuous variable) will then be classified as low or high or not applicable (categorical variable)”. - Addition of the following: “the MSI predictive markers to be assessed are: MLH-1, MSH-2, MSH-6, and PMS-2”. |
|  |  |  |  |  | Section 8.2.3 | - Replacement of “urine pregnancy test for women of childbearing potential” with “urine or serum pregnancy test for women of childbearing potential who are sexually active”. |
|  |  |  |  |  | Section 8.2.5 | - Replacement of “urine pregnancy test for women of childbearing potential” with “urine or serum pregnancy test for women of childbearing potential who are sexually active”. - Addition of the following: “PDL-1 expression will be classified as negative or positive or not applicable (categorical variable)”. - Addition of the following: “CD4+, CD8+ and CD3+ T cell infiltration will be quantified in mm^2^ in the most abundant tumor-infiltrating area in both, the stroma and the tumor, of the surgical specimen (continuous variable)”. - Addition of the following: “in case of pathologic complete response, the T cell count corresponding to the tumor area of the surgical specimen will be noted as “not applicable”. - Addition of the following: “after the recruitment of around 5 patients, a cut-off value will be agreed upon and the numerical values corresponding to T cell infiltration (continuous variable) will then be classified as low or high or not applicable (categorical variable)”. - Addition of the following: “the Becker et al. tumor regression grading system will be used to categorize the amount of regressive changes after cytotoxic treatment as follows:   - 1a. No residual tumor/ tumor bed + chemotherapy effect;   - 1b. <10% residual tumor/ tumor bed + chemotherapy effect;   - 2. 10-50% residual tumor/ tumor bed + chemotherapy effect;   - 3. >50% residual tumor/ tumor bed **±** chemotherapy effect”. |
|  |  |  |  |  | Section 8.3.1 | - Replacement of “urine pregnancy test for women of childbearing potential must be performed at baseline and least every month during treatment” with “urine or serum pregnancy test for women of childbearing potential and who are sexually active must be performed at baseline and least every month during treatment”. |
|  |  |  |  |  | Section 10.2 | - Replacement of “urine pregnancy test for women of childbearing potential must be performed at baseline and least every month during treatment” with “urine or serum pregnancy test for women of childbearing potential and who are sexually active must be performed at baseline and least every month during treatment”. |
|  |  |  |  | 28 August 2018 | Table 1 | - Replacement of “PDL-1 expression” with “PDL-1 expression on tumor and infiltrating immune T cells”. |
|  |  |  |  |  | Section 8.2.1 | - - - - - Addition of the following: “PDL-1 expression will be evaluated on tumor cells and infiltrating immune T cells”. |
|  |  |  |  |  | Section 8.2.2 | - Addition of the following: “PDL-1 expression will be evaluated on tumor cells and infiltrating immune T cells”. |
|  |  |  |  |  | Section 8.2.5 | - Addition of the following: “PDL-1 expression will be evaluated on tumor cells and infiltrating immune T cells”. |
|  |  |  |  |  | Section 8.3.2 | - Replacement of “PDL-1 expression” with “PDL-1 expression on tumor and infiltrating immune T cells”. |
|  |  |  |  |  | Figure 1 | - Replacement of “Sigmoidoscopy and biopsy: PDL-1 expression” with “Sigmoidoscopy and biopsy: PDL-1 expression on tumor and infiltrating immune T cells”. |
|  |  |  |  |  |  | - Replacement of “Resected Specimen: PDL-1 expression” with “Resected Specimen: PDL-1 expression on tumor and infiltrating immune T cells”. |
|  |  |  |  | 04 October 2018 | Synopsis | - Replacement of “bolus fluorouracil 400 mg/m^2^ followed by a 48-hour infusion of fluorouracil 2,400 mg/m^2^” with only “a 48-hour infusion of fluorouracil 2,400 mg/m^2^”. |
|  |  |  |  |  | Table 1 | - Addition of the following: “HCV polymerase chain reaction (PCR) for patients positive for HCV antibodies (to differentiate active infection from past infection with HCV)”. |
|  |  |  |  |  | Section 7.2 | - Replacement of “bolus fluorouracil 400 mg/m^2^ followed by a 48-hour infusion of fluorouracil 2,400 mg/m^2^” with only “a 48-hour infusion of fluorouracil 2,400 mg/m^2^”. |
|  |  |  |  |  | Section 8.2.1 | - Addition of the following: “Conduct of HCV polymerase chain reaction (PCR) for patients positive for HCV antibodies (to differentiate active infection from past infection with HCV)”. |
|  |  |  |  |  | Section 8.2.3 | - Replacement of “bolus fluorouracil 400 mg/m^2^ followed by a 48-hour infusion of fluorouracil 2,400 mg/m^2^” with only “a 48-hour infusion of fluorouracil 2,400 mg/m^2^”. |
|  |  |  |  |  | Section 8.2.5 | - Adjustment of the description of the Becker et al. tumor regression grading system as follows:   - 1a. No viable tumor/ tumor bed + chemotherapy effect;   - 1b. <10% viable tumor/ tumor bed + chemotherapy effect;   - 2. 10-50% viable tumor/ tumor bed + chemotherapy effect;   - 3. >50% viable tumor/ tumor bed ± chemotherapy effect”. |
|  |  |  |  |  | Section 12.7.2 | - Replacement of “bolus fluorouracil 400 mg/m^2^ followed by a 48-hour infusion of fluorouracil 2,400 mg/m^2^” with only “a 48-hour infusion of fluorouracil 2,400 mg/m^2^”. |
|  |  |  |  |  | Figure 1 | - Replacement of “bolus fluorouracil 400 mg/m^2^ followed by a 48-hour infusion of fluorouracil 2,400 mg/m^2^” with only “a 48-hour infusion of fluorouracil 2,400 mg/m^2^.” |
|  | |  | |  | Section 20 Appendices | - In the section 20.0 appendices Cyril Tohmeh and Elie Choulliard are removed from the study and Ghassan Chakhtoura added as Co- Investigator. |

# SIGNATURE PAGE

This clinical study is carried out in accordance with the international guidelines on Good Clinical Practice (ICH-GCP) and in compliance with applicable regulatory authority requirements. It is confirmed that the clinical study will be carried out and documented in accordance with this study protocol.

| **Principal Investigator**  Ali Shamseddine, MD, FRCP |  |
| --- | --- |
|  | Date, signature |

# SIGNATURE PAGE FOR INVESTIGATORS

**Declaration of the Principal Investigator**

I have read and understood this clinical study protocol and agree to the following:

- To adhere to the ethical and scientific principles of good clinical practice, and the principles of the Declaration of Helsinki, the local laws and regulations, and the applicable regulatory requirements.
- To conduct the clinical study as set out in the protocol.

This includes:

- To wait until I have received approval from the appropriate Independent Ethics Committee / Institutional Review Board (IEC/IRB) before enrolling any patient in this study.
- To obtain informed consent of each patient prior to any study-related procedures performed.
- To permit study-related monitoring, audits, IEC/IRB review, and regulatory authority inspections.
- To provide direct access to all study-related records, source documents, and patient files for the monitor, auditor, IEC/IRB, or regulatory authority upon request.
- To understand that changes to the clinical study protocol must be made in the form of an amendment that has the prior written approval of AUB and, as applicable, of the appropriate IEC/IRB and regulatory authority.
- To comply with the reporting obligations for Adverse Events / Serious Adverse Events (AE/SAEs).

I understand that all documentation that has not been previously published will be kept in the strictest confidence. This documentation includes the Clinical Study Protocol, Investigator’s Brochure, Case Report Forms, and other scientific data.

| **Co-Principal Investigator**  <Name> |  |
| --- | --- |
|  | Date, signature |

Investigator stamp:

# TABLE OF CONTENTS

[OVERVIEW OF AMENDMENTS INTEGRATED IN THE PROTOCOL TEXT OF VERSION 1.0 OF 22 SEPTEMBER 2017 2](#__RefHeading___Toc526418249)

[SIGNATURE PAGE 10](#__RefHeading___Toc526418250)

[SIGNATURE PAGE FOR INVESTIGATORS 11](#__RefHeading___Toc526418251)

[TABLE OF CONTENTS 12](#__RefHeading___Toc526418252)

[LIST OF TABLES 16](#__RefHeading___Toc526418253)

[LIST OF FIGURES 16](#__RefHeading___Toc526418254)

[ABBREVIATIONS AND DEFINITIONS OF TERMS 17](#__RefHeading___Toc526418255)

[PROTOCOL SYNOPSIS 21](#__RefHeading___Toc526418256)

[1. INTRODUCTION AND BACKGROUND INFORMATION 30](#__RefHeading___Toc526418257)

[1.1. Disease and context 30](#__RefHeading___Toc526418258)

[1.2. Target 30](#__RefHeading___Toc526418259)

[1.3. Non-clinical and clinical information 31](#__RefHeading___Toc526418260)

[1.3.1. Non-clinical information 31](#__RefHeading___Toc526418261)

[1.3.2. Clinical information 32](#__RefHeading___Toc526418262)

[1.4. Study rationale 33](#__RefHeading___Toc526418263)

[1.4.1. Clinical study rationale 33](#__RefHeading___Toc526418264)

[1.4.2. Primary endpoint rationale 34](#__RefHeading___Toc526418265)

[1.4.3. Neoadjuvant treatment rationale 34](#__RefHeading___Toc526418266)

[1.4.4. Short-course radiation therapy rationale 34](#__RefHeading___Toc526418267)

[1.4.5. Avelumab dose regimen rationale 35](#__RefHeading___Toc526418268)

[2. STUDY OBJECTIVES 35](#__RefHeading___Toc526418269)

[2.1. Primary objective 35](#__RefHeading___Toc526418270)

[2.2. Secondary objectives 35](#__RefHeading___Toc526418271)

[3. STUDY DESIGN 36](#__RefHeading___Toc526418272)

[3.1. Design 36](#__RefHeading___Toc526418273)

[3.2. Endpoints 38](#__RefHeading___Toc526418274)

[3.3. Measures to avoid / minimize bias 38](#__RefHeading___Toc526418275)

[3.4. Study investigational center(s) 38](#__RefHeading___Toc526418276)

[3.5. Scientific committee / Data and safety monitoring board / 39](#__RefHeading___Toc526418277)

[4. DURATION AND DATES OF THE STUDY 40](#__RefHeading___Toc526418278)

[5. STUDY POPULATION 40](#__RefHeading___Toc526418279)

[5.1. Number of patients 40](#__RefHeading___Toc526418280)

[5.2. Eligibility criteria 41](#__RefHeading___Toc526418281)

[5.2.1. Inclusion criteria 41](#__RefHeading___Toc526418282)

[5.2.2. Exclusion criteria 42](#__RefHeading___Toc526418283)

[5.3. Duration of patient participation 44](#__RefHeading___Toc526418284)

[5.4. Discontinuation criteria / stopping rule(s) 44](#__RefHeading___Toc526418285)

[5.5. Lost to follow-up 45](#__RefHeading___Toc526418286)

[6. INVESTIGATIONAL MEDICINAL PRODUCT (IMP) 45](#__RefHeading___Toc526418287)

[6.1. Description of IMP 45](#__RefHeading___Toc526418288)

[6.2. Presentation 46](#__RefHeading___Toc526418289)

[6.2.1. Packaging 46](#__RefHeading___Toc526418290)

[6.2.2. Labelling 46](#__RefHeading___Toc526418291)

[6.3. Management of IMP 46](#__RefHeading___Toc526418292)

[6.3.1. Shipment and receipt 46](#__RefHeading___Toc526418293)

[6.3.2. Storage requirements 47](#__RefHeading___Toc526418294)

[6.3.3. IMP re-supplying 48](#__RefHeading___Toc526418295)

[6.3.4. IMP return, destruction and recall 48](#__RefHeading___Toc526418296)

[6.4. Treatment of patients 49](#__RefHeading___Toc526418297)

[6.4.1. Methods for assigning patients to treatment groups 49](#__RefHeading___Toc526418298)

[6.4.2. Dispensing 49](#__RefHeading___Toc526418299)

[6.4.3. Misuse / overdose 58](#__RefHeading___Toc526418300)

[6.5. IMP accountability 58](#__RefHeading___Toc526418301)

[6.6. Randomization codes and procedures for blinding 58](#__RefHeading___Toc526418302)

[7. PRIOR AND CONCOMITANT MEDICATION 58](#__RefHeading___Toc526418303)

[7.1. Prior medication 58](#__RefHeading___Toc526418304)

[7.2. Concomitant medication 59](#__RefHeading___Toc526418305)

[8. STUDY PLAN 60](#__RefHeading___Toc526418306)

[8.1. Patient recruitment 60](#__RefHeading___Toc526418307)

[8.1.1. Informed Consent 60](#__RefHeading___Toc526418308)

[8.1.2. Patient enrolment 61](#__RefHeading___Toc526418309)

[8.1.3. Patient allocation 61](#__RefHeading___Toc526418310)

[8.1.4. Replacement of early withdrawals 61](#__RefHeading___Toc526418311)

[8.2. Schedule of visits 61](#__RefHeading___Toc526418312)

[8.2.1. Selection/patient recruitment: visit 1 62](#__RefHeading___Toc526418313)

[8.2.2. Radiation therapy period: visits 2 to 7 64](#__RefHeading___Toc526418314)

[8.2.3. Treatment period: visits 8 to 13 66](#__RefHeading___Toc526418315)

[8.2.4. End of treatment visit including study completion and premature withdrawal: visit 13 67](#__RefHeading___Toc526418316)

[8.2.5. Surgery: visit 14 67](#__RefHeading___Toc526418317)

[8.2.6. Follow-up visits: every 3 months after surgery 70](#__RefHeading___Toc526418318)

[8.2.7. End of study visit (last patient visit / last patient contact) 71](#__RefHeading___Toc526418319)

[8.3. Assessments 71](#__RefHeading___Toc526418320)

[8.3.1. Assessment performed at site 71](#__RefHeading___Toc526418321)

[8.3.2. Centralized assessments 73](#__RefHeading___Toc526418322)

[8.4 Compliance with the study plan 73](#__RefHeading___Toc526418323)

[9. SAFETY 74](#__RefHeading___Toc526418324)

[9.1. Safety reference document 74](#__RefHeading___Toc526418325)

[9.2. Benefit / risk information 74](#__RefHeading___Toc526418326)

[9.2.1. Expected risk(s) related to the IMP(s) 74](#__RefHeading___Toc526418327)

[9.2.2. Other expected risk(s) 79](#__RefHeading___Toc526418328)

[9.2.3. Benefit / risk balance 83](#__RefHeading___Toc526418329)

[9.3. Risk minimization actions throughout the protocol 84](#__RefHeading___Toc526418330)

[9.4. Alternative therapeutic management - emergencies handling 84](#__RefHeading___Toc526418331)

[9.5. Definition and reporting of (serious) adverse events 85](#__RefHeading___Toc526418332)

[9.5.1. Definition of adverse event and serious adverse event 85](#__RefHeading___Toc526418333)

[9.5.2. Period of (serious) adverse event data collection 86](#__RefHeading___Toc526418334)

[9.5.3. Recording and description of (serious) adverse event 86](#__RefHeading___Toc526418335)

[9.5.4. Procedures for reporting serious adverse events 87](#__RefHeading___Toc526418336)

[9.5.5. Medical contacts 88](#__RefHeading___Toc526418337)

[9.5.6. Follow-up of adverse events 89](#__RefHeading___Toc526418338)

[9.6. Pregnancy 89](#__RefHeading___Toc526418339)

[9.7. Regulatory safety requirements 91](#__RefHeading___Toc526418340)

[10. PARAMETERS AND ASSESSMENT CRITERIA 91](#__RefHeading___Toc526418341)

[10.1. Assessment of efficacy 91](#__RefHeading___Toc526418342)

[10.2. Assessment of safety 91](#__RefHeading___Toc526418343)

[10.3. Exploratory endpoints 93](#__RefHeading___Toc526418344)

[11. DATA MANAGEMENT 93](#__RefHeading___Toc526418345)

[11.1. eCRF completion 93](#__RefHeading___Toc526418346)

[11.1.1. Introduction 93](#__RefHeading___Toc526418347)

[11.1.2. General instructions 93](#__RefHeading___Toc526418348)

[11.1.3. Specific Case Report Form instructions 96](#__RefHeading___Toc526418349)

[11.2. CRF and data handling 96](#__RefHeading___Toc526418350)

[12. STATISTICS 96](#__RefHeading___Toc526418351)

[12.1. Statistical analysis plan 96](#__RefHeading___Toc526418352)

[12.2. Sample size determination 97](#__RefHeading___Toc526418353)

[12.3. Randomization 98](#__RefHeading___Toc526418354)

[12.4. Protocol deviations and analysis sets 98](#__RefHeading___Toc526418355)

[12.5. General rules for handling of missing or inconsistent data 98](#__RefHeading___Toc526418356)

[12.6. Demographic and baseline characteristics 99](#__RefHeading___Toc526418357)

[12.6.1. Demographic characteristics, medical history and diagnoses 99](#__RefHeading___Toc526418358)

[12.6.2. Previous treatments 99](#__RefHeading___Toc526418359)

[12.6.3. Baseline efficacy variables 99](#__RefHeading___Toc526418360)

[12.6.4. Baseline safety variables 100](#__RefHeading___Toc526418361)

[12.7. IMP and concomitant medications 100](#__RefHeading___Toc526418362)

[12.7.1. Extent of exposure 100](#__RefHeading___Toc526418363)

[12.7.2. Concomitant medications 100](#__RefHeading___Toc526418364)

[12.8. Efficacy analysis 101](#__RefHeading___Toc526418365)

[12.8.1. Primary efficacy variable(s) 101](#__RefHeading___Toc526418366)

[12.8.2. Secondary efficacy variable(s) 101](#__RefHeading___Toc526418367)

[12.9. Safety analysis 102](#__RefHeading___Toc526418368)

[12.10. Pharmacokinetics, PK/PD, and analysis 102](#__RefHeading___Toc526418369)

[13. STUDY REPORT 102](#__RefHeading___Toc526418370)

[14. CONFIDENTIALITY AND PUBLICATION 103](#__RefHeading___Toc526418371)

[14.1. Patient confidentiality 103](#__RefHeading___Toc526418372)

[14.2. Use of information 103](#__RefHeading___Toc526418373)

[15. ARCHIVING 104](#__RefHeading___Toc526418374)

[16. RESPONSIBILITIES OF PARTICIPANTS 104](#__RefHeading___Toc526418375)

[16.1. Responsibilities of the investigator(s) 104](#__RefHeading___Toc526418376)

[16.2. Responsibilities of the CRA 107](#__RefHeading___Toc526418377)

[17. ETHICS AND REGULATORY CONSIDERATIONS 107](#__RefHeading___Toc526418378)

[18. AUDIT AND INSPECTION 109](#__RefHeading___Toc526418379)

[19. REFERENCES 111](#__RefHeading___Toc526418380)

[20. APPENDICES 114](#__RefHeading___Toc526418381)

[20.0. List of principal investigators and co-principal investigators 114](#__RefHeading___Toc526418382)

[20.1. Pharmacy manual – investigator sponsored study use of avelumab 118](#__RefHeading___Toc526418383)

[20.2. Guidelines and definitions for reporting (S)AE by the investigator 119](#__RefHeading___Toc526418384)

[20.3. (Serious) Adverse Event form 120](#__RefHeading___Toc526418385)

[20.4. Pregnancy form 121](#__RefHeading___Toc526418386)

[20.5. Parent-child/fetus AE form 122](#__RefHeading___Toc526418387)

# LIST OF TABLES

[Table 1 Study flowchart 27](#__RefHeading___Toc526418388)

[Table 2 Recommended dose modifications of avelumab for infusion-related reactions 52](#__RefHeading___Toc526418389)

[Table 3 Management of immune-mediated adverse reactions 53](#__RefHeading___Toc526418390)

[Table 4 Expected adverse reactions in patients treated with avelumab in clinical studies 76](#__RefHeading___Toc526418391)

[Table 5 Other expected adverse reactions reported in the clinical avelumab program outside the pooled safety dataset 78](#__RefHeading___Toc526418392)

[Table 6 Common and less common adverse events with mFOLFOX-6 79](#__RefHeading___Toc526418393)

[Table 7 NCI-CTCAE grading for the common adverse events 80](#__RefHeading___Toc526418394)

# LIST OF FIGURES

[Figure 1 Study design 37](#__RefHeading___Toc526418395)

# ABBREVIATIONS AND DEFINITIONS OF TERMS

| ACTH | : | Adrenocorticotropic Hormone |
| --- | --- | --- |
| ADCC | : | Antibody-Dependent Cell-Mediated Cytotoxicity |
| ADL | : | Activities of Daily Living |
| AE | : | Adverse Event |
| ALT | : | Alanine Aminotransferase |
| ANC | : | Absolute Neutrophil Count |
| AST | : | Aspartate Aminotransferase |
| ATC | : | Anatomical Therapeutic Chemical |
| AUB | : | American University of Beirut |
| AUBMC | : | American University of Beirut Medical Center |
| BNP |  | B-type Natriuretic Peptide |
| BSA | : | Body Surface Area |
| BUN | : | Blood Urea Nitrogen |
| CA | : | Competent Authority |
| CA 19-9 | : | Cancer Antigen 19-9 |
| CAP | : | College of American Pathologists |
| CBC | : | Complete Blood Count |
| CCS | : | Colorectal Cancer Subscale |
| CEA | : | Carcinoembryonic Antigen |
| CI | : | Confidence Interval |
| CK-MB | : | Creatine Kinase MB |
| CRA | : | Clinical Research Associate |
| CRF | : | Case Report Form |
| CRO | : | Contract Research Organization |
| CT | : | Computed Tomography |
| CTV | : | Clinical Target Volume |
| D | : | Day |
| DFS | : | Disease Free Survival |
| DSMB | : | Data and Safety Monitoring Board |
| EC/IRB | : | Ethics Committee (EC) or Institutional Review Board (IRB) |
| ECG | : | Electrocardiogram |
| eCRF | : | electronic Case Report Form |
| EWB | : | Emotional Well-Being |
| FACT-C |  | Functional Assessment of Cancer Therapy - For patients with Colorectal cancer |
| FAS | : | Full-Analysis Set |
| FPI | : | First-Patient In |
| FSH | : | Follicle-Stimulating Hormone |
| FWB | : | Functional Well-Being |
| GCP | : | Good Clinical Practice |
| GGT | : | Gamma Glutamyl Transferase |
| GH | : | Growth Hormone |
| GTV | : | Gross Tumor Volume |
| Hbg | : | Hemoglobin |
| HBV | : | Hepatitis B Virus |
| HCV | : | Hepatitis C Virus |
| IB | : | Investigator’s Brochure |
| ICH | : | International Conference on Harmonization of Technical Requirements for Registration of Pharmaceuticals for Human Use |
| IGF-1 | : | Insulin-like Growth Factor 1 |
| IgG | : | Immunoglobulin G |
| IMP | : | Investigational Medicinal Product |
| IMRT | : | Intensity-Modulated Radiotherapy |
| irAE | : | Immune Related Adverse Event |
| IRB | : | Institutional Review Board |
| ISF | : | Investigator Site File |
| IV | : | Intravenous |
| LH | : | Luteinizing Hormone |
| LLN | : | Lower Limit of Normal |
| LPFD | : | Last Patient First Dose |
| MedDRA | : | Medical Dictionary for Regulatory Activities |
| mFOLFOX-6 | : | Modified FOLFOX-6. FOLFOX-6 is a combination of chemotherapy drugs: oxaliplatin, leucovorin and fluorouracil |
| MRI | : | Magnetic Resonance Imaging |
| MSI | : | Microsatellite Instability |
| NCCN | : | National Comprehensive Cancer Network |
| NCI-CTCAE | : | National Cancer Institute-Common Terminology Criteria for Adverse Event |
| ND | : | Not Done |
| NSAID | : | Non-steroidal Anti‑Inflammatory Drug |
| OS | : | Overall Survival |
| pCR | : | Pathologic Complete Response |
| PCR  PD-L1 | : | Polymerase Chain Reaction  Programmed Death Ligand-1 |
| PET | : | Positron Emission Tomography |
| PFS | : | Progression-Free Survival |
| PP | : | Per Protocol |
| PRL | : | Prolactin |
| PT | : | Preferred Terms |
| PT | : | Prothrombin Time |
| PTT | : | Partial Thromboplastin Time |
| PTV | : | Planning Target Volume |
| PWB | : | Physical Well-Being |
| SAE | : | Serious Adverse Event |
| SAP | : | Statistical Analysis Plan |
| SGOT | : | Serum Glutamic Oxaloacetic Transaminase |
| SGPT | : | Serum Glutamic-Pyruvic Transaminase |
| SCRT | : | Short-course Chemo-Radiotherapy |
| SOC | : | System Organ Class |
| SWB | : | Social/Family Well-Being |
| T4 | : | Thyroxine |
| TME | : | Total Mesorectal Excision |
| TPN | : | Total Parenteral Nutrition |
| TRG | : | Tumor Regression Grade |
| TSH | : | Thyroid Stimulating Hormone |
| UK | : | Unknown |
| ULN | : | Upper Limit of Normal |

# PROTOCOL SYNOPSIS

| **CLINICAL STUDY No.** | BIO-2017-0422 |
| --- | --- |
| **TITLE** | Short-course radiation followed by mFOLFOX-6 plus avelumab for locally-advanced rectal adenocarcinoma |
| **STUDY PHASE** | Phase II |
| **SPONSOR** | **Investigator Sponsored Study**  American University of Beirut (AUB)  Beirut, Lebanon |
| **PRINCIPAL INVESTIGATOR** | Dr. Ali Shamseddine, MD, FRCP  Department of Internal Medicine, Division of Hematology/Oncology  American University of Beirut Medical Center (AUBMC)  Beirut P.O. Box 11-0236, Lebanon  Phone: +961 1 350 000 (Ext.: 5390)  Email: as04@aub.edu.lb |
| **COUNTRIES/**  **CENTERS** | 2 sites in Lebanon  1 site in Jordan |
| **INVESTIGATIONAL MEDICINAL PRODUCT** | IMP description: avelumab (intravenous infusion), available as a solution of 200 mg/10 mL (20 mg/mL) in single-dose vials. |
| **TREATMENT COURSE** | Treatment of patients (Product, dose and mode of administration):   1. Short-course radiation therapy: 25 Gy in 5 fractions. 2. mFOLFOX-6 chemotherapy 6 cycles plus avelumab 10 mg/kg every 2 weeks in a successive manner such that mFOLFOX is administered 30-minutes after avelumab has already been administered..   Surgery: total mesorectal excision (TME, open, laparoscopic or robotic). |
| **CLINICAL STUDY DESIGN** | Open-label, single-arm multicenter and 2-stage phase II study conducted among patients with locally-advanced, potentially resectable rectal adenocarcinoma |
| **OBJECTIVES** | Primary: To evaluate the rate of pathologic complete response (pCR) rate following short-course radiation then mFOLFOX-6/avelumab.  Secondary:   1. To identify the proportion of patients who remain progression free at 3 years. 2. To explore changes in PD-L1 expression and T-cell infiltration. 3. To evaluate the safety and tolerability of mFOLFOX-6/avelumab. 4. To assess the quality of life of the patients in a neoadjuvant setting with avelumab. |
| **NUMBER OF PATIENTS** **AND GROUPS** | 44 |
| **CLINICAL STUDY POPULATION** | Diagnosis: locally-advanced, potentially resectable rectal adenocarcinoma  Inclusion criteria:   1. Patients aged ≥18 years. 2. Locally-advanced rectal cancer (cT2 N1-3, cT3 N0-3, evidence of extramural vascular or mesorectal fascia involvement). 3. <15 cm from anal verge. 4. Histologically proven rectal adenocarcinoma. 5. ECOG performance score ≤ 1. 6. Have adequate organ function by meeting the following:  - Absolute neutrophil count (ANC) ≥ 1.5 × 10^9^/L; - Platelet count ≥ 100 × 10^9^/L; - Hemoglobin ≥ 9 g/dL; - Total bilirubin level ≤ 1.5 × the upper limit of normal (ULN) range; - AST and ALT levels ≤ 2.5 × ULN or AST and ALT levels ≤ 5 x ULN (for subjects with documented metastatic disease to the liver); - Estimated creatinine clearance ≥ 30 mL/min according to the Cockcroft-Gault formula (or local institutional standard method.  1. Negative serum or urine pregnancy test at screening for women of childbearing potential who are sexually active. 2. Highly effective contraception for both male and female subjects throughout the study and for at least 30 days after last avelumab treatment administration if the risk of conception exists.   Exclusion criteria:   1. Distant metastasis (M1). 2. Patients with T2 N0 or T4. 3. Recurrent rectal cancer. 4. Symptoms or history of peripheral neuropathy. 5. Prior radiotherapy or chemotherapy. 6. Current use of immunosuppressive medication, except for the following:  - Intranasal, inhaled, topical steroids, or local steroid injection (e.g., intra-articular injection); - Systemic corticosteroids at physiologic doses ≤ 10 mg/day of prednisone or equivalent; - Steroids as premedication for hypersensitivity reactions (e.g., CT scan premedication).  1. Active autoimmune disease that might deteriorate when receiving an immuno-stimulatory agent. Patients with diabetes type I, vitiligo, psoriasis, or hypo- or hyperthyroid diseases not requiring immunosuppressive treatment are eligible. 2. Vaccination within 4 weeks of the first dose of avelumab and while on trials is prohibited except for administration of inactivated vaccines. 3. Active infection requiring systemic therapy. 4. Known history of testing positive for the human immunodeficiency virus or known acquired immunodeficiency syndrome. 5. Hepatitis B virus (HBV) or hepatitis C virus (HCV) infection at screening (positive HBV surface antigen or HCV RNA if anti-HCV antibody screening test positive). 6. Known prior severe hypersensitivity to investigational product or any component in its formulations, including known severe hypersensitivity reactions to monoclonal antibodies (NCI CTCAE v4.03 Grade ≥ 3). 7. Clinically significant (i.e., active) cardiovascular disease: cerebral vascular accident/stroke (< 6 months prior to enrollment), myocardial infarction (< 6 months prior to enrollment), unstable angina, congestive heart failure (≥ New York Heart Association Classification Class II), or serious cardiac arrhythmia requiring medication. 8. Persisting toxicity related to prior therapy (NCI CTCAE v. 4.03 Grade > 1); however, alopecia, sensory neuropathy Grade ≤ 2, or other Grade ≤ 2 not constituting a safety risk based on investigator’s judgment are acceptable. 9. Prior organ transplantation including allogenic stem-cell transplantation. 10. Other severe acute or chronic medical conditions including immune colitis, inflammatory bowel disease, immune pneumonitis, pulmonary fibrosis or psychiatric conditions including recent (within the past year) or active suicidal ideation or behavior; or laboratory abnormalities that may increase the risk associated with study participation or study treatment administration or may interfere with the interpretation of study results and, in the judgment of the investigator, would make the patient inappropriate for entry into this study. 11. Concurrent treatment with a non-permitted drug. 12. Patients suspected by the physician that he/she will not compliant to the protocol conduct. 13. Pregnant or breastfeeding patients. 14. Patient participating in another clinical trial. 15. Patient who is not willing to sign the consent form. 16. Any psychiatric condition that would prohibit the understanding or rendering of informed consent. 17. Legal incapacity or limited legal capacity patients receiving other oncology specific medication not authorized in the protocol. |
| **DURATION OF PATIENT PARTICIPATION** | Treatment period: 16 weeks  Follow-up period: after surgery every 3 months until the study has reached Year 3  Total: 36 months |
| **TREATMENT SCHEDULE** | 1. Week 1 ± 3 days: D1-5: radiotherapy 25 Gy in 5 fractions 2. Week 2 ± 3 days: repeat biopsy 3. Week 3 (D15) ± 3 days:  - mFOLFOX-6: Oxaliplatin 85 mg/m^2^ in a 2-hour infusion   Leucovorin 400 mg/m² over 2 hours  48-hour infusion of fluorouracil 2,400 mg/m²   - Avelumab 10 mg/kg every 2 weeks (first administration at D15, for a total of 6 cycles)  1. Chemotherapy protocol every 2 weeks ± 3 days (weeks 3, 5, 7, 9 ,11 and 13) 2. Week 16 or 17 ± 3 days (2 to 3 weeks after last cycle of chemotherapy + avelumab): TME |
| **CLINICAL STUDY PLANNING** | Screening: no screening period  First-Patient In (FPI): 20 July 2018  First Patient First Dose - First Patient Last Dose: 10 weeks after first dose  First-Patient Last Visit: 3 years from FPI  Last-Patient In: 20 January 2020  Last Patient Last Visit: 02 May 2023  Database lock: 02 June 2023  Key statistics: 20 July 2023  Clinical Study Report approved: 20 October 2023 |
| **ENDPOINTS AND EVALUATION PARAMETERS** | Primary endpoint: proportion of patients who achieve a pathological complete response, defined as no viable tumor cells on the resected specimen.  Secondary endpoints:   1. Progression-free survival (PFS) at 3 years will be estimated with the Kaplan-Meier method and presented with the 95% confidence interval (CI). 2. Evaluation of response by obtaining Tumor Regression Grade (TRG). 3. Evaluation of biomarkers: CD4+, CD8+ and CD3+ T cell infiltration, and changes in PD-L1 expression. 4. Frequency, severity, and attribution of adverse events related to avelumab in a neoadjuvant setting. 5. Quality of life assessment using FACT-C questionnaire. |
| **STATISTICS** | A Simon’s two-stage optimal design with a null hypothesis pCR rate ≤ 16% versus the alternative that pCR rate ≥ 35%, a type I error of 0.05 and a power of 80%. The results of the first 13 patients eligible for the primary efficacy analysis will be assessed in stage 1. If 2 or less patients achieved pCR, the study will be stopped, otherwise another 23 (eligible) patients will be added to the study (stage 2) for a total of 36 eligible for the primary efficacy analysis. If overall 10 or more patients achieved pCR, then the null hypothesis (the percentage of pCR is ≤16%) will be rejected. In total, 44 patients will be enrolled into the study considering that 15 to 20% of the patients might be not eligible for the primary analysis. .  Then, the percentage of patients achieving pCR will be calculated along with its one-sided 95% confidence interval. Also using Kaplan-Meier method, the median PFS will be estimated along with its 95% confidence interval. Exploratory variables will be analyzed according to their scale of measurement by using mean ± standard deviation or frequency distribution for numeric and categorical variables respectively.  Frequency distribution for AEs and SAEs will be presented per cycle and per patient. Similarly this will be done for the SAE of grade 3 or above combined. |

Table 1 Study flowchart

|  | **Inclusion Visit** | **Treatment Visit** | **Treatment Visit** | **Treatment Visit** | **Treatment Visit** | **Treatment Visit** | **Treatment Visit** | **Treatment Visit** | **End of Treatment Visit** | **Surgery** | **Follow-up Visit** | **End of Study Visit** |
| --- | --- | --- | --- | --- | --- | --- | --- | --- | --- | --- | --- | --- |
|  | **V1** | **V2 to V6**  **Week 1**  **D1-D5**  **± 3 days** | **V7**  **Week 2**  **D10**  **± 3 days** | **V8**  **Week 3 D15**  **± 3 days** | **V9**  **Week 5**  **± 3 days** | **V10**  **Week 7**  **± 3 days** | **V11**  **Week 9**  **± 3 days** | **V12**  **Week 11**  **± 3 days** | **V13**  **Week 13**  **± 3 days** | **V14**  **Week 16 or 17**  **± 3 days** | **Every 3 months**  **± 3 days** | **V27**  **Year 3**  **± 3 days** |
| **Informed consent** | x |  |  |  |  |  |  |  |  |  |  |  |
| **Inclusion/exclusion criteria** | x |  |  |  |  |  |  |  |  |  |  |  |
| **Disease diagnosis** | x |  |  |  |  |  |  |  |  |  |  |  |
| **Alcohol consumption and smoking history** | x |  |  |  |  |  |  |  |  |  |  |  |
| **Medical history** | x |  |  |  |  |  |  |  |  |  |  |  |
|  |  |  |  |  |  |  |  |  |  |  |  |  |
| **Physical / clinical examination** |  |  |  |  |  |  |  |  |  |  |  |  |
| Vital signs | x |  |  | x | x | x | x | x | x |  | x | x |
| Weight | x |  |  | x | x | x | x | x | x |  | x | x |
| Pelvic MRI (rectal protocol) | x |  |  |  |  |  |  |  |  | x |  |  |
| CT scan or PET of the chest and the abdomen | x |  |  |  |  |  |  |  |  |  |  |  |
|  |  |  |  |  |  |  |  |  |  |  |  |  |
| **Laboratory tests** |  |  |  |  |  |  |  |  |  |  |  |  |
| Hematology tests (CBC) | x |  |  | x | x | x | x | x | x | x |  |  |
| Biochemistry tests* | x |  |  | x | x | x | x | x | x | x |  |  |
| Urine or serum pregnancy test** | x |  |  | x |  | x |  | x |  | x |  |  |
| Free T4 and TSH¥ | x |  |  |  |  | x |  |  |  | x |  |  |
| Tumor markers (CEA and CA 19-9) | x |  |  |  |  |  |  |  | x |  | x | x |
| PT and PTT |  |  |  |  |  |  |  |  |  | x |  |  |
| Hepatitis B virus surface antigen, Hepatitis C virus antibodies | x |  |  |  |  |  |  |  |  |  |  |  |
| HCV polymerase chain reaction  (PCR) for patients positive for HCV antibodies (to differentiate active infection from past infection with HCV) | x |  |  |  |  |  |  |  |  |  |  |  |
|  |  |  |  |  |  |  |  |  |  |  |  |  |
| **Pathology assessment** |  |  |  |  |  |  |  |  |  |  |  |  |
| Sigmoidoscopy |  |  | x |  |  |  |  |  |  |  |  |  |
| PD-L1 expression on Tumor and Immune Infiltrating T cells & CD4+, CD8+ and CD3+ T cell infiltration^†^ | x |  | x |  |  |  |  |  |  | x |  |  |
| Microsatellite instability^‡^ | x |  | x |  |  |  |  |  |  |  |  |  |
| Tumor Regression Grading |  |  |  |  |  |  |  |  |  | x |  |  |
|  |  |  |  |  |  |  |  |  |  |  |  |  |
| **Treatment** |  |  |  |  |  |  |  |  |  |  |  |  |
| Prior treatments | x |  |  |  |  |  |  |  |  |  |  |  |
| Radiotherapy 25 Gy in 5 fractions |  | x |  |  |  |  |  |  |  |  |  |  |
| Concomitant medications (mFOLFOX-6 and anti-emetics) |  |  |  | x | x | x | x | x | x |  |  |  |
| Other concomitant medications |  |  |  | x | x | x | x | x | x | x | x | x |
| Surgery TME (open, laparoscopic or robotic) |  |  |  |  |  |  |  |  |  | x |  |  |
| Achievement of a pathological complete response after surgery |  |  |  |  |  |  |  |  |  | x |  |  |
|  |  |  |  |  |  |  |  |  |  |  |  |  |
| **IMP administration** |  |  |  |  |  |  |  |  |  |  |  |  |
| Avelumab premedication |  |  |  | x | x | x | x | x | x |  |  |  |
| Avelumab 10 mg/Kg every 2 weeks (6 cycles) |  |  |  | x | x | x | x | x | x |  |  |  |
|  |  |  |  |  |  |  |  |  |  |  |  |  |
| **Adverse event collection** |  | x | x | x | x | x | x | x | x | x | x | x |
|  |  |  |  |  |  |  |  |  |  |  |  |  |
| **Patient-Reported Outcome** |  |  |  |  |  |  |  |  |  |  |  |  |
| Quality of Life assessment (FACT-C) | x |  |  | x | x | x | x | x | x |  | x | x |
|  |  |  |  |  |  |  |  |  |  |  |  |  |
| **Patient survival status** |  |  |  |  |  |  |  |  |  |  | x | x |
| **Disease status** |  |  |  |  |  |  |  |  |  |  | x | x |

* Biochemistry tests include: blood urea nitrogen, creatinine, electrolytes, SGPT, SGOT, GGT, alkaline phosphatase, and bilirubin.

** Urine or serum pregnancy test for women of childbearing potential and who are sexually active must be performed at baseline and at least every month during treatment.

¥ Free T4 and TSH must be performed at baseline and at least every 8 weeks during treatment and at end of treatment or 30 days post-treatment safety follow-up.

† PD-L1 expression on Tumor and Immune Infiltrating T cells & CD4+, CD8+ and CD3+ T cell infiltration are determined on baseline biopsy, D10 and surgical resection specimen.

‡ The microsatellite instability (MSI or MMR status) is determined once on either the baseline biopsy or D10 biopsy.

# INTRODUCTION AND BACKGROUND INFORMATION

## Disease and context

Colorectal cancers are among the most common cancers worldwide, and there is a high mortality rate for advanced-stage disease. The incidence and mortality rates have been steadily declining over the past two decades, largely through advances in screening and improvements in treatment. However, rectal cancer remains a significant cause of morbidity and mortality worldwide (Kothari N et al., 2015). The significant majority of rectal cancers are carcinomas, which are primarily comprised of adenocarcinomas. For the best outcome, these cancers require a multidisciplinary approach including chemotherapy, radiation, and surgery (Kothari N et al., 2015).

Patients with locally-advanced rectal cancer are treated with neoadjuvant chemoradiation before total mesorectal excision (TME) to induce tumor regression, increase the probability of achieving resection with negative margins, and reduce the risk of local recurrence. Post-operative adjuvant chemotherapy is also recommended in these patients to reduce the risk of distant metastasis. This multimodality treatment achieves high levels of local tumor control and good long-term survival (Sauer R et al., 2004). However, TME is associated with some mortality, morbidity, and long-term sequelae that have a substantial negative effect on quality of life (Williams NS et al., 1983).

Also and despite the use of peri-operative radiation and chemotherapy, outcomes for patients with locally-advanced rectal adenocarcinoma remain poor with 3-year disease-free survival (DFS) approximately 50% (Bujko K et al., 2016a; Nilsson et al., 2013). The use of single-agent immune checkpoint inhibitors in microsatellite-stable colorectal cancer has shown limited efficacy. However, the strategy of combining immune checkpoint inhibition with systemic chemotherapy after radiation is a novel approach that has the potential to overcome these limitations.

## Target

Avelumab, (company code: MSB0010718C) the investigational medicinal product (IMP), is a fully human antibody (calculated molecular weight of 143,832 Dalton) of the immunoglobulin G 1 (IgG1) isotype. It specifically targets and binds the programmed death ligand-1 (PD-L1), the ligand for PD-1, and blocks the interaction between PD-L1 and PD-1. This removes the suppressive effects of PD-L1 on anti-tumor CD8+ T cells, resulting in the restoration of cytotoxic T cell response. Monoclonal antibodies targeting PD-1 and PD-L1 have shown antitumor activity in a range of human solid tumors, including melanoma and cancers of the lung, kidney, head and neck, bladder, stomach, and breast (Chen I et al., 2015).

Avelumab has received [orphan drug](https://en.wikipedia.org/wiki/Orphan_drug) designation by the European Medicines Agency for the treatment of [gastric cancer](https://en.wikipedia.org/wiki/Gastric_cancer) in January 2017. The US Food and Drug Administration (FDA) approved it on 23 March 2017 for [Merkel-cell carcinoma](https://en.wikipedia.org/wiki/Merkel-cell_carcinoma), an aggressive type of skin cancer, under the name Bavencio® (a trademark of Merck KGaA, Darmstadt, Germany). Although other agents targeting PD-L1 are in clinical development, to the best of our knowledge, avelumab is the first human anti-PD-L1 IgG1 antibody with a native Fc region. This means it is capable of inducing antibody-dependent cell-mediated cytotoxicity (ADCC) against PD-L1+ tumor cells *in vitro* and elimination of ADCC potential *in vivo* significantly reduced anti-tumor activity (Boyerinas B, 2015).

## Non-clinical and clinical information

### Non-clinical information

Based on the known role of PD-L1 in the suppression of T cell responses and the strong correlation between PD-L1 expression and prognosis in cancer, the blockade of the PD-L1/PD-1 interaction is viewed as a highly promising strategy for cancer immunotherapy (Topalian et al., 2012). Merck KGaA has developed a fully human IgG1 antibody (avelumab) with neutralizing activity against PD-L1. The non-clinical pharmacology investigations have shown that avelumab functionally enhances T cell activation in vitro and significantly inhibits the growth of PD-L1 expressing tumors in vivo. In agreement with the hypothesis that PD-L1 neutralization acts to release anti-tumor T cells from immune suppression, the anti-tumor effects of avelumab *in vivo* were found to be primarily mediated by CD8+ T cells, as highlighted by the observation that the *in vivo* depletion of this cell type was sufficient to completely abrogate anti-tumor activity.

Depletion of CD8+ T cells also eliminated the synergistic efficacy of avelumab when given in combination with radiotherapy, suggesting that this combination synergizes through cooperative immune-enhancing mechanisms. As a second mode of action, avelumab is capable of stimulating ADCC activity against PD-L1+ tumor cells *in vitro* and elimination of ADCC potential *in vivo* significantly reduced anti-tumor activity.

In summary, the available preclinical data demonstrate that avelumab is capable of inhibiting tumor growth in vivo when applied as a monotherapy and its efficacy can be further enhanced *via* combination with standard-of-care therapies. Avelumab has the potential to offer significant clinical benefits to cancer subjects through its ability to release anti-tumor CD8+ T cells from the suppressive effects of PD-L1 in the tumor microenvironment. The investigation of this agent in human clinical trials is therefore warranted.

Overall, the non-clinical safety profile established for avelumab is considered adequate to support the use of avelumab in the planned therapeutic indication in humans.

Additional information can be found in the Investigator’s Brochure.

### Clinical information

As of the date of avelumab’s IB (31 March 2017), there are no completed clinical trials to report. No clinical trial is conducted over avelumab in locally-advanced rectal adenocarcinoma. However, avelumab at a dose of 10 mg/kg once every 2 weeks has demonstrated meaningful clinical activity across various tumor types (lung cancer, ovarian cancer, gastric cancer, gastroesophageal junction cancer, urothelial carcinoma, mesothelioma, and adrenocortical carcinoma) and treatment settings. Across the aforementioned tumor types, responses with avelumab were typically observed early during treatment and appear durable in nature, including ongoing responses lasting > 1 year in several of the different cohorts. Overall, many responders were still experiencing ongoing response at the time of the data cutoff for the analysis.

As for safety data from subjects with different tumor types treated with avelumab, they suggest an acceptable safety profile of the compound. Most of the observed events were either in line with those expected in subjects with advanced solid tumors or with similar class effects of mAb blocking the PD-1/PD-L1 axis. Infusion-related reactions including drug hypersensitivity reactions and immune-mediated adverse reactions (immune-related pneumonitis, immune-related colitis, immune-related hepatitis, immune-related endocrinopathies (thyroid disorders, adrenal insufficiency, new onset type I diabetes mellitus, pituitary disorders), immune-related nephritis and renal dysfunction and other immune-related adverse events (AEs: myositis, myocarditis, Guillain-Barré syndrome, uveitis) have been identified as important risks for avelumab. The known and potential risks and benefits to the patient are further discussed in Section 9.2.

## Study rationale

### Clinical study rationale

The traditional treatment of locally-advanced but operable rectal carcinoma consists of a combination of peri-operative long-course chemoradiation therapy, or alternatively a short-course radiation therapy to be followed by a surgery (TME), then by adjuvant chemotherapy for 4 months (Benson AB et al., 2016). All randomized phase III clinical trials failed to answer the question of the efficacy of adjuvant chemotherapy to impact on the outcome as DFS or overall survival (OS) (Breugmon AJ et al., 2015). Also, there is a good correlation between the rate of pCR and OS (de Castro et al., 2012). In an attempt to increase pCR in rectal cancer, the strategy of total neoadjuvant therapy was adapted in many phase II trials with increasing pCR up to 38% and in many ongoing phase III trials (Garcia-Aguilar J et al., 2015).

In POLISH III, a randomized clinical trial, short-course radiation therapy followed by 3 cycles of FOLFOX was compared to the regular long course with chemotherapy in improving OS at 3 years. The newly supported published data were in favor of total neoadjuvant therapy with short-course followed by chemotherapy followed by surgery (Bujko K et al., 2016a; Bujko K et al., 2016b). Also, all ongoing trials including RAPIDO and OPERA trials are testing the same hypothesis with total neoadjuvant therapy but without using immunotherapy like avelumab (Nilsson PJ et al., 2013; Smith JJ et al., 2015).

In view of the above data, a phase II clinical study is highly required to evaluate the pCR rate following short-course radiation (5 days), then mFOLFOX-6 chemotherapy protocol combined with 10 mg/kg of avelumab (once every 2 weeks for 6 cycles) in patients with locall-advanced rectal adenocarcinoma before undergoing a TME.

### Primary endpoint rationale

The pathologic complete response (pCR) rate following pre-operative treatment has been shown to be a surrogate for DFS in locally-advanced rectal cancer (Mass M et al., 2010). The use of pCR as a primary endpoint with evaluation of biomarkers such as changes in PD-L1 expression, CD8+ cell-infiltration associated with therapy, will enable a rapid proof of concept. It will also enable feasibility assessment to test the hypothesis that the addition of avelumab to mFOLFOX-6 chemotherapy administered following short-course radiation for locally-advanced rectal cancer will improve the disease’s post-operative outcomes and provide the basis for the planning of future randomized studies.

### Neoadjuvant treatment rationale

The tolerance of pre-operative chemotherapy and the compliance are much better than post-operative therapy, and the need to achieve a more pathologic response rate prior to surgery may translate to an increase in survival. Also, of the combination approaches that have currently been explored for avelumab, chemotherapy with FOLFOX and radiation therapy showed the better tumor growth inhibition. In particular, radiation therapy was found to be a highly synergistic combination capable of causing complete regression of established tumors with the potential to generate anti-tumor immune memory. Additional information can be found in the Investigator’s Brochure.

### Short-course radiation therapy rationale

The use of short-course is intended to induce the inflammatory reaction and increase the immune-mediated reaction while the use of both oxaliplatin and avelumab will maintain the immune and anti-tumor effect which can translate into an increased pCR. Also, the NCCN guidelines for the management of locally-advanced disease included the option of a short-course chemo-radiotherapy (SCRT) as data show tumor down-staging with SCRT (NCCN, 2016; Nilsson PJ et al., 2013).

### Avelumab dose regimen rationale

A dose of 10 mg/kg of avelumab, IV, once every 2 weeks, was selected for the expansion cohorts of phase I trials, the phase II pivotal trial (EMR100070-003), and the ongoing phase III trials based on the preliminary pharmacokinetic (PK), target occupancy, and clinical safety data collected in the clinical trials (Heery CR et al., 2017; Investigator’s Brochure of avelumab, version 7 of 31 March 2017).

Indeed, avelumab 10 mg/kg once every 2 weeks has demonstrated meaningful clinical activity across various tumor types and treatment settings. Regardless of the tumor type, responses with avelumab were observed early during treatment and appear durable in nature, including ongoing responses >1 year in several of the cohorts. Overall, many responders were still having ongoing response at the time of database lock. Based on the above analyses, a dose of 10 mg/kg IV once every 2 weeks was considered to have a favorable risk benefit profile and thus represents an appropriate dose for the present study. Additional information can be found in the Investigator’s Brochure.

# STUDY OBJECTIVES

## Primary objective

The primary objective of the study is to evaluate the pathologic complete response (pCR) rate following short-course radiation then mFOLFOX-6/avelumab.

## Secondary objectives

The secondary objectives of the study are:

1. To identify the proportion of patients who remain progression free at 3 years.
2. To explore changes in PD-L1 expression and T-cell infiltration.
3. To evaluate the safety and tolerability of mFOLFOX-6/avelumab.
4. To assess the quality of life of the patients in a neoadjuvant setting with avelumab.

# STUDY DESIGN

## Design

This is an open-label, single-arm multicenter and stage-2 phase II study investigating short-course radiation therapy (25 Gy in 5 fractions), followed 2 weeks later by 6 cycles of mFOLFOX-6 chemotherapy plus avelumab (10 mg/kg) given every 2 weeks in a successive manner such that mFOLFOX is administered 30 minutes after avelumab has been administered in patients with locally-advanced, potentially resectable rectal adenocarcinoma (**Figure 1**).

No screening period is planned.

All chemotherapy agents will be taken intravenously (infusion) every 2 weeks for 6 cycles during the treatment period (12 weeks), or until intolerable toxicity or withdrawal of patient’s informed consent. TME will performed 2 to 3 weeks after the last cycle of chemotherapy plus avelumab, depending on the patient’s condition and laboratory exams.

Each patient will be followed up for 3 years after surgery.

No reference therapy or placebo will be used in the trial.

Written informed consent must be obtained before any study specific medical procedure is performed.

Figure 1 Study design

**Week 1, D1-D5**

Radiotherapy: 25 Gy in 5 fractions

**Week 2 ± 3 days, D10**

Sigmoidoscopy and biopsy (PD-L1 expression on tumor and infiltrating immune T cells, CD4+, CD8+ and CD3+ T cell infiltration†, and MSI‡)

**Week 16 or 17 ± 3 days**

(2-3 weeks after last cycle of chemotherapy + avelumab)

Surgery: total mesorectal excision

(open, laparoscopic or robotic)

+

Resected specimen: PD-L1 expression on tumor and infiltrating immune T cells, CD4+, CD8+ and CD45RO+ T cell infiltration†, and TRG*

**Week 3 ± 3 days**

**mFOLFOX-6:** Oxaliplatin 85 mg/m^2^ in a 2-hour infusion

Leucovorin 400 mg/m^2^ over 2 hours

by a 48-hour infusion of fluorouracil 2,400 mg/m**^2^**

+

**Avelumab** 10 mg/Kg every 2 weeks ± 3 days in a successive manner such that mFOLFOX is administered 30-minutes after avelumab has already been administered

(6 cycles, first administration at D15)

12 weeks

‡ The microsatellite instability (MSI or MMR status) is determined once on either the baseline biopsy or D10 biopsy.

## Endpoints

The primary endpoint is the proportion of patients who achieve a pathological complete response, defined as no viable tumor cells onthe resected specimen.

The secondary endpoints are:

1. Progression-free survival (PFS) at 3 years will be estimated with the Kaplan-Meier method and presented with the 95% confidence interval (CI).
2. Evaluation of response by obtaining Tumor Regression Grade (TRG).
3. Evaluation of biomarkers: CD4+, CD8+ and CD3+ T cell infiltration, and changes in PD-L1 expression.
4. Frequency, severity, and attribution of AEs related to avelumab in neoadjuvant setting.
5. Quality of life assessment using the questionnaire for the functional assessment of cancer therapy for patients with colorectal cancer (FACT-C, English version 4 of 16 November 2007 or Arabic version 4 of 03 September 2014).

TRG system aims to categorize the amount of regressive changes after cytotoxic treatment mostly refer onto the amount of therapy induced fibrosis in relation to residual tumor or the estimated percentage of residual tumor in relation to the previous tumor site (Thies S & Langer R, 2013).

## Measures to avoid / minimize bias

Neither randomization nor blinding will be performed in this study.

## Study investigational center(s)

A total of 3 centers are planned in 2 countries:

1. 2 sites in Lebanon
2. 1 site in Jordan

Each center is expected to enroll 12 eligible patients.

The Investigator sites/units should have immediate access to equipment and staff for resuscitation.

## Scientific committee / Data and safety monitoring board /

A scientific committee is in charge of the protocol design, the approval of the final protocol version and of the amendments, if any. The scientific committee will also discuss the results before writing all reports and publications.

A Data and Safety Monitoring Board (DSMB) will be constituted to ensure patient safety.

The DSMB is an expert committee, independent from the investigators and the sponsor of the clinical study, which periodically examines data accumulated during progress of the study and ensures that benefice/risk ratio remains acceptable for participating patients.

Primarily, the DSMB will approve the study protocol BIO-2017-0467 in order to confirm the study design and data safety monitoring plan.

The DSMB will independently make its recommendations for continuation, termination or modification of the conduct of the study.

The sponsor will timely provide the members of the DSMB with the safety data of the patients. In particular, a report will be sent to the DSMB members after the first stage is completed and 13 patients are accrued from all centers to decide about proceeding with the second stage of the study. By that time, they will provide the sponsor with written recommendations whether or not to proceed to the inclusion of the following patients and will give recommendations to change or not the methods of administration. An annual meeting will be held thereafter, andDSMB advices could be sought for any questions regarding the safety and well-being of the patients who will receive the study treatment.

The DSMB will include a board steering recommendations between the following experts, independent from any study involvement (apart from sponsor, subsidiaries or study team):

1. A physician, MD (Dr. Ahmad Awada) specialist in gastrointestinal oncology at Institut Jules Bordet, Brussels, Belgium
2. A physician, MD (Dr. Alain Hendlisz) specialist in gastrointestinal oncology at Institut Jules Bordet, Brussels, Belgium
3. A physician, MD (Dr. Ghassan Abou-Alfa) specialist in gastrointestinal oncology at Memorial Sloan Kettering Cancer Center
4. A biostatistician (Dr. Hani Tamim, PhD), Associate Professor of Medicine, and Director of the Biostatistics Unit at the Clinical Research Institute at the Faculty of Medicine, American University of Beirut (AUB)

All experts will necessarily have a prior clinical extended experience in immunotherapy in cancer and a significant involvement in clinical research and investigation development methods.

# DURATION AND DATES OF THE STUDY

The duration of this study depends on the results of the interim analysis, i.e., the probability of early termination, if only 2 or fewer patients achieve pCR at stage one (65.37% based on the study design). Interim analysis will be done on 13 patients once the surgery on the 13th patient is completed. The remaining patients will be enrolled based on the result of that analysis. The study duration is expected to be 4.5 years if the 2 stages of the study will be done. The first patient’s first visit is expected on 20 July 2018, and last patient last visit in on 02 May 2023. The 4.5-year duration includes 18 months of enrolment and 3 years of follow-up.

# STUDY POPULATION

## Number of patients

In this study, 44 patients with locally-advanced, potentially resectable rectal adenocarcinoma will be enrolled in total.

In this multicenter study, a mean number of 12 patients is expected to be enrolled by each center.

Since Simon’s two-stage design (Simon R, 1989) will be used:

1. 13 patients will be accrued in the first stage from all centers, until April 2019. If there are 2 or fewer patients with a pCR among these 13 patients, the study will be stopped.
2. Otherwise, 31 additional patients will be accrued for a total of 44.

The enrolment objectives for each center will take into consideration the enrolment period, i.e., 3 patients per month.

## Eligibility criteria

Before any study-related procedure is undertaken, written informed consent must be obtained (see Section 9.1.1).

For a patient to be eligible, all of the inclusion criteria and none of the exclusion criteria must be met.

### Inclusion criteria

For patient inclusion, all of the following criteria must be met:

1. Signed and dated informed consent form,
2. Patients aged ≥18 years.
3. Locally-advanced rectal cancer (cT2 N1-3, cT3 N0-3, evidence of extramural vascular or mesorectal fascia involvement).
4. <15 cm from anal verge
5. Histologically proven rectal adenocarcinoma.
6. ECOG performance score ≤ 1.
7. Have adequate organ function by meeting the following:
   - Absolute neutrophil count (ANC) ≥ 1.5 × 10^9^/L;
   - Platelet count ≥ 100 × 10^9^/L;
   - Hemoglobin ≥ 9 g/dL;
   - Total bilirubin level ≤ 1.5 × the upper limit of normal (ULN) range;
   - AST and ALT levels ≤ 2.5 × ULN or AST and ALT levels ≤ 5 x ULN (for subjects with documented metastatic disease to the liver);
   - Estimated creatinine clearance ≥ 30 mL/min according to the Cockcroft-Gault formula (or local institutional standard method).
8. Negative serum or urine pregnancy test at screening for women of childbearing potential who are sexually active.
9. Highly effective contraception for both male and female subjects throughout the study and for at least 30 days after last avelumab treatment administration if the risk of conception exists.

### Exclusion criteria

Patients will not be eligible for inclusion in this study if any of the following criteria is met:

1. Distant metastasis (M1).
2. Patients with T2 N0 or T4.
3. Recurrent rectal cancer.
4. Symptoms or history of peripheral neuropathy.
5. Prior radiotherapy or chemotherapy.
6. Current use of immunosuppressive medication, except for the following:
   - Intranasal, inhaled, topical steroids, or local steroid injection (e.g., intra-articular injection);
   - Systemic corticosteroids at physiologic doses ≤ 10 mg/day of prednisone or equivalent;
   - Steroids as premedication for hypersensitivity reactions (e.g., CT scan premedication).
7. Active autoimmune disease that might deteriorate when receiving an immuno-stimulatory agent. Patients with diabetes type I, vitiligo, psoriasis, or hypo- or hyperthyroid diseases not requiring immunosuppressive treatment are eligible.
8. Vaccination within 4 weeks of the first dose of avelumab and while on trials is prohibited except for administration of inactivated vaccines.
9. Active infection requiring systemic therapy.
10. Known history of testing positive for the human immunodeficiency virus or known acquired immunodeficiency syndrome.
11. Hepatitis B virus (HBV) or hepatitis C virus (HCV) infection at screening (positive HBV surface antigen or HCV RNA if anti-HCV antibody screening test positive).
12. Known prior severe hypersensitivity to investigational product or any component in its formulations, including known severe hypersensitivity reactions to monoclonal antibodies (NCI CTCAE v4.03 Grade ≥ 3).
13. Clinically significant (i.e., active) cardiovascular disease: cerebral vascular accident/stroke (< 6 months prior to enrollment), myocardial infarction (< 6 months prior to enrollment), unstable angina, congestive heart failure (≥ New York Heart Association Classification Class II), or serious cardiac arrhythmia requiring medication.
14. Persisting toxicity related to prior therapy (NCI CTCAE v. 4.03 Grade > 1); however, alopecia, sensory neuropathy Grade ≤ 2, or other Grade ≤ 2 not constituting a safety risk based on investigator’s judgment are acceptable.
15. Prior organ transplantation including allogenic stem-cell transplantation.
16. Other severe acute or chronic medical conditions including immune colitis, inflammatory bowel disease, immune pneumonitis, pulmonary fibrosis or psychiatric conditions including recent (within the past year) or active suicidal ideation or behavior; or laboratory abnormalities that may increase the risk associated with study participation or study treatment administration or may interfere with the interpretation of study results and, in the judgment of the investigator, would make the patient inappropriate for entry into this study.
17. Concurrent treatment with a non-permitted drug.
18. Patients suspected by the physician that he/she will not compliant to the protocol conduct.
19. Pregnant or breastfeeding patients.
20. Patient participating in another clinical trial.
21. Patient who is not willing to sign the consent form.
22. Any psychiatric condition that would prohibit the understanding or rendering of informed consent.
23. Legal incapacity or limited legal capacity patients receiving other oncology specific medication not authorized in the protocol.

## Duration of patient participation

For each patient, the participation will last 3 years.

The study planning is as follows:

1. Screening: no screening period
2. First-Patient In (FPI): 20 July 2018
3. First Patient First - Dose First Patient Last Dose: 10 weeks after first dose
4. First-Patient Last Visit: 3 years from FPI
5. Last-Patient In: 20 January 2020
6. Last Patient Last Visit: 02 May 2023
7. Follow-up: every 3 months after the surgery
8. Database lock: 02 June 2023
9. Key statistics: 20 July 2023
10. Clinical Study Report approved: 20 October 2023

## Discontinuation criteria / stopping rule(s)

The Investigator must document the reason of any patient withdrawal or discontinuation by a narrative description.

A patient may be discontinued from the study at any time for one or more of the following reasons:

1. Occurrence of an AE that, to the judgment of the Investigator or the Principal Investigator, may interfere with study conduct or study results
2. Protocol deviation that could invalidate the interpretation of the results
3. Lack of efficacy if relevant, to be defined on a “case by case” basis
4. Withdrawal of consent for any reason, at any time
5. Lost to follow-up
6. Administrative problems
7. Investigator decision in patient best interest
8. Pregnancy
9. Death

Data to be collected are detailed in Section 8.

The study can be stopped depending on the results of the interim analysis in this 2-stage study.

If a patient withdraws from the study or if the treatment is discontinued, the Investigator will make every effort to complete the final evaluation by performing an "end of study visit". All evaluation results, the date and a narrative description of the reason(s) for discontinuation must be recorded in the source documents and in the electronic Case Report Form (eCRF).

## Lost to follow-up

Investigators should make every effort to obtain a maximum of information on patients lost to follow-up. All attempts will be documented in the patient’s medical records.

# INVESTIGATIONAL MEDICINAL PRODUCT (IMP)

## Description of IMP

The IMP, avelumab, will be supplied, free of charge, by Merck KGaA*.*

It has not been assigned any ATC code.

The active pharmaceutical ingredient in avelumab drug product (also referred to as MSB0010718C) is a fully human antibody of the IgG1 isotype that specifically targets and blocks PD-L1.

The antibody is produced by mammalian cell culture in a serum-free growth medium. The antibody is purified by affinity, ion-exchange, and mixed-mode chromatography. The process also includes specific viral inactivation and removal steps. The antibody is then transferred into formulation buffer and brought to the desired concentration.

Avelumab is manufactured by EMD Serono, Inc.

Avelumab drug product is a sterile solution intended for IV infusion. It is available in the form of a sterile, clear, and colorless concentrate for solution presented at concentration of 20 mg/mL in European Pharmacopeia (Ph. Eur.) and United States Pharmacopeia (USP) type I glass vials closed with a rubber stopper and sealed with an aluminum Flip Off® crimp seal closure.

Each single-use vial contains 200 mg of avelumab as a preservative-free acetate-buffered solution (pH 5.2) containing Mannitol, and Polysorbate 20 (Tween20).

For avelumab drug product, only excipients that conform to the current Ph. Eur. and/or the current USP are used.

For more information, see the Highlights of Prescribing Information of Bavencio® (avelumab) and the Investigator’s Brochure of avelumab (IB, version 7 of 31 March 2017).

## Presentation

### Packaging

Each box will contain a treatment needed for one patient and for one infusion of avelumab.

In total, 6 boxes should be prepared for each patient, that is to say, for 6 cycles of avelumab.

### Labelling

The proposed label will contain the information required by and in accordance with Annex 13 GMP and the applicable local legislation.

The label will be adapted to local requirements and to the size of the IMP package, and translated into the local language when legally required

## Management of IMP

### Shipment and receipt

The frequency at which the IMP will be supplied to each site will be adapted to the enrolment rate of the site and will take into consideration the expiry / re-test date of the IMP**.**

The IMP will be shipped to the Investigator/Hospital Pharmacist by Merck KGaA in accordance with local requirements and as soon as the initiation of the site is validated by the Principal Investigator.

Shipment of IMP will be done at refrigerated temperature (+2/+8°C), with temperature monitoring device.

Upon receipt of treatment supplies, the Investigator or Pharmacist will inventory the supplies and complete the shipping form. Should any abnormality of the supply boxes be observed, the Investigator or Pharmacist must immediately inform the Clinical Research Associate (CRA). The shipping form must be returned to the Principal Investigator as instructed.

### Storage requirements

Avelumab should be stored in a secured limited-access area and maintained under refrigeration at 2°C to 8°C for no more than 24 hours from the time of dilution. If refrigerated, the diluted solution should be allowed to come to room temperature prior to administration.

Avelumab drug product stored at room temperature (23°C to 27°C) or higher temperatures for extended periods of time might be subject to degradation.

Avelumab should be protected from light and has not to be frozen. Rough shaking of the solution must be avoided.

For administration in clinical trials, avelumab drug product must be diluted with 0.9% saline solution (sodium chloride injection) supplied in an infusion bag; alternatively a 0.45% saline solution can be used if needed. The chemical and physical in-use stability for the infusion solution of avelumab in 0.45% or 0.9% saline solution has been demonstrated for a total of 24 hours at room temperature. However, from a microbiological point of view, the diluted solution should be used immediately. If not used immediately, it can be considered that the diluted product is sufficiently stable from a microbiological perspective for up to 8 hours when stored at ambient room temperature or up to 24 hours at 2°C to 8°C.

For more information, see the Highlights of Prescribing Information of Bavencio® (avelumab) and the IB of avelumab (version 7 of 31 March 2017).

The Investigator and/or the Hospital Pharmacist are responsible for the appropriate storage of avelumab in each center of the study.

If the required storage conditions are not respected, the Investigator and/or the Hospital Pharmacist must immediately inform the Principal Investigator. Any temperature deviation must be reported within 1 working day using a temperature deviation form. For any temperature deviation, a written approval from the Principal Investigator must be obtained prior to any dispensation/administration. During the evaluation of the temperature deviation, the concerned IMP must be placed under quarantine. If the decision is not to use the quarantined IMP, the vials will remain under quarantine until shipment back to Merck KGaA.

### IMP re-supplying

The investigational sites will be re-supplied with IMPs according to their respective enrolment rates.

### IMP return, destruction and recall

- - - 1. ***Return***

Used and unused IMPs will be returned back to Merck KGaA, Switzerland, at the closing visit. IMPs return will be organized by the Hospital Pharmacist and the CRA. The Hospital Pharmacist or a delegate should be available at the predetermined date and time when the boxes will be collected from the investigational center.

The used/unused IMPs (vials and original containers) can be destroyed locally at the site ONLY if a certified incinerator is available. If an on-site destruction is required, the site must have a written authorization from the Principal Investigator for destruction which will be filed along with the certificate of destruction in the IMP section of the Pharmacy site file.

At the end of the study, a final reconciliation between delivered, dispensed, used/unused and returned IMPs will be conducted by the Principal Investigator.

- - - 1. ***Destruction***

The written authorization for destruction will be filed along with the certificate of destruction in the IMP section of the Investigator Site File (ISF).

Used and unused syringes will not be returned back to the Principal Investigator. In each investigational site, syringes will be collected in special containers and destroyed according to the internal procedure of the center.

- - - 1. ***Recall***

If an IMP batch is suspected to be defective, the Investigator/Hospital Pharmacist will be immediately informed by the Coordinating Investigator.

The CRA will organize with the Investigator/Hospital Pharmacist the return of the concerned batch(es) as per the return procedure. Depending on the study status, new batch(es) may be sent to the investigational site.

## Treatment of patients

### Methods for assigning patients to treatment groups

Medicinal products will be numbered and treatment will be assigned to eligible patients according to the ascending order of their enrolment.

The steps for assigning patients to treatment groups are described in Section 8.1.3.

### Dispensing

Under no circumstances will the Investigator allow the IMP to be used other than as directed in the protocol.

- - - 1. ***IMP reconstitution***

The required dose of avelumab is based on the patient’s bodyweight on each visit as illustrated by **Equation (1):**

Required dose (mg) = 10 (mg/kg) x Patient weight (kg)

Avelumab must be diluted with 0.9% saline solution (sodium chloride injection) supplied in an infusion bag; alternatively a 0.45% saline solution can be used if needed. The volume of avelumab and 0.9% saline solution or 0.45% saline solution needed for the dilution can be calculated using the following equations:

Volume of avelumab (mL) = Required dose from **Equation (1)** in mg

20 mg/mL

**Equation (2):**

Volume of normal saline (mL) = 250 mL – Volume of avelumab (mL)

**Equation (3) :**

No other drugs should be added to the solution for infusion containing avelumab.

Detailed information on infusion bags and medical devices to be used for the preparation of the dilutions and subsequent administration will be provided in the manual of preparation.

To prepare the dilutions, subsequent preparation steps must be accomplished by adequately trained personnel under a laminar flow box using aseptic techniques:

1. Discard any partially used or empty vials.
2. Prior to the preparation of the dilution for final infusion, allow each vial to equilibrate to room temperature.
3. Use a disposable syringe equipped with a needle of suitable size to remove a volume of sodium chloride solution to be replaced by avelumab from the infusion bag and discard the removed solution.
4. Use a new disposable syringe equipped with a needle of suitable size to inject a volume of avelumab drug product identical to the discarded volume of sodium chloride solution into the infusion bag.
5. Gently invert the mixture 10 times.
6. Infusion bags must not be shaken, in order to avoid foaming or excessive shearing of the protein solution.
7. Assigned dose levels and concrete volumes: details are available in the pharmacy manual (Appendix 20.1).

Also, prior to administration, reconstituted IMP (avelumab + saline solution) should be inspected visually by the third party for particulate, cloudiness, color or deposits:

1. The preparation must be carefully inspected as it should result in a homogeneous looking clear and colorless to slightly yellow solution, free of visible particles.
2. The IMP must not be administered if it does not conform to the description. The Investigator/Hospital Pharmacist should immediately alert the CRA/ Principal Investigator.

For additional information on IMP preparation, see IB of avelumab (version 7 of 31 March 2017).

- - - 1. ***Method, route of administration, treatment dose and schedule***

Only intravenous route is allowed for the administration of the reconstituted solution of IMP.

Avelumab 10 mg/kg is administered to every patient as an intravenous infusion over 60 minutes, followed 30 minutes later by mFOLFOX-6 chemotherapy every 2 weeks as per the institution’s standard of practice.

No other drugs should be added to the solution for infusion containing avelumab, and no other drugs should be co-administered through the same intravenous line.

The infusion should be done through an intravenous line containing a sterile, non-pyrogenic, low protein binding in-line filter (pore size of 0.2 micron).

For additional information on IMP administration, see the Highlights of Prescribing Information of Bavencio® (avelumab) and the IB of avelumab (version 7 of 31 March 2017).

Only one infusion of IMP will be administered to each patient per cycle, i.e. 6 infusions throughout the study.

Total administered volume, date and time of administration (start, end) are to be reported in the eCRF, patient’s medical records and/or in the IMP accountability records (when applicable).

IMP label has to be stuck on corresponding visit page in the patient’s medical record.

- - - 1. ***Dose modifications***

Recommended dose modifications of AVELUMAB for adverse reactions are provided in Tables 2 and 3.

Table 2 Recommended dose modifications of avelumab for infusion-related reactions

| NCI‑CTCAE Grade | Treatment Modification for Avelumab |
| --- | --- |
| **Grade 1 – mild**  Mild transient reaction; infusion interruption not indicated; intervention not indicated. | Decrease the avelumab infusion rate by 50% and monitor closely for any worsening. |
| **Grade 2 – moderate**  Therapy or infusion interruption indicated but responds promptly to symptomatic treatment (for example, antihistamines, NSAIDs, narcotics, IV fluids); prophylactic medications indicated for ≤ 24 hours. | Temporarily discontinue avelumab infusion.  Resume infusion at 50% of previous rate once infusion-related reaction has resolved or decreased to at least Grade 1 in severity, and monitor closely for any worsening. |
| **Grade 3 or Grade 4 – severe or life‑threatening**  Grade 3: Prolonged (for example, not rapidly responsive to symptomatic medication and/or brief interruption of infusion); recurrence of symptoms following initial improvement; hospitalization indicated for clinical sequelae.  Grade 4: Life-threatening consequences; urgent intervention indicated. | Stop avelumab infusion immediately and disconnect infusion tubing from the subject.  Subjects have to be withdrawn immediately from study avelumab and must not receive any further avelumab treatment. |
| If avelumab infusion rate has been decreased by 50% or interrupted due to an infusion reaction, it must remain decreased for the next scheduled infusion.  If no infusion reaction is observed in the next scheduled infusion, the infusion rate may be returned to baseline at the subsequent infusions based on Investigator’s medical judgment.  If hypersensitivity reaction occurs, the subject must be treated according to the best available medical practice. | |
| Abbreviations: IV=Intravenous; NCI-CTCAE=National Cancer Institute-Common Terminology Criteria for Adverse Event; NSAIDs= Non-Steroidal Anti‑Inflammatory Drugs. | |

Table 3 Management of immune-mediated adverse reactions

| Gastrointestinal irAEs | | | | | | | | |
| --- | --- | --- | --- | --- | --- | --- | --- | --- |
| Severity of Diarrhea/Colitis  (NCI-CTCAE v4) | | | Initial Management | | Follow-up Management | | | |
| **Grade 1**  Diarrhea: < 4 stools/day over Baseline  Colitis: asymptomatic | | | Continue avelumab therapy.  Symptomatic treatment (e.g. loperamide). | | Close monitoring for worsening symptoms.  Educate subject to report worsening immediately.  If worsens, treat as Grade 2, 3 or 4. | | | |
| **Grade 2**  Diarrhea: 4 to 6 stools per day over Baseline; IV fluids indicated < 24 hours; not interfering with ADL  Colitis: abdominal pain; blood in stool | | | Withhold avelumab therapy.  Symptomatic treatment. | | If improves to Grade ≤ 1:  Resume avelumab therapy  If persists > 5-7 days or recurs:  Treat as Grade 3 or 4. | | | |
| **Grade 3 to 4**  Diarrhea (Grade 3): ≥ 7 stools per day over Baseline; incontinence; IV fluids ≥ 24 h; interfering with ADL  Colitis (Grade 3): severe abdominal pain, medical intervention indicated, peritoneal signs  Grade 4: life-threatening, perforation | | | Withhold avelumab for Grade 3.  Permanently discontinue avelumab for Grade 4 or recurrent Grade 3.  1.0 to 2.0 mg/kg/day prednisone IV or equivalent.  Add prophylactic antibiotics for opportunistic infections  Consider lower endoscopy. | | If improves:  Continue steroids until Grade ≤ 1, then taper over at least 1 month; resume avelumab therapy following steroids taper (for initial Grade 3).  If worsens, persists > 3 to 5 days, or recurs after improvement:  Add infliximab 5mg/kg (if no contraindication). Note: infliximab should not be used in cases of perforation or sepsis. | | | |
| Dermatological irAEs | | | | | | | | |
| Grade of Rash  (NCI-CTCAE v4) | | | Initial Management | | Follow-up Management | | | |
| **Grade 1 to 2**  Covering ≤ 30% body surface area | | | Continue avelumab therapy Symptomatic therapy (for example, antihistamines, topical steroids) | | If persists > 1 to 2 weeks or recurs:  Withhold avelumab therapy  Consider skin biopsy  Consider 0.5-1.0 mg/kg/day prednisone or equivalent. Once improving, taper steroids over at least 1 month, consider prophylactic antibiotics for opportunistic infections, and resume avelumab therapy following steroids taper.  If worsens:  Treat as Grade 3 to 4. | | | |
| **Grade 3 to 4**  Grade 3: Covering > 30% body surface area;  Grade 4: Life threatening consequences | | | Withhold avelumab for Grade 3.  Permanently discontinue for Grade 4 or recurrent Grade 3.  Consider skin biopsy.  Dermatology consult.  1.0 to 2.0 mg/kg/day prednisone or equivalent.  Add prophylactic antibiotics for opportunistic infections. | | If improves to Grade ≤ 1:  Taper steroids over at least 1 month; resume avelumab therapy following steroids taper (for initial Grade 3). | | | |
| Pulmonary irAEs | | | | | | | | |
| Grade of Pneumonitis  (NCI-CTCAE v4) | | | Initial Management | | Follow-up Management | | | |
| **Grade 1**  Radiographic changes only | | | Consider withholding avelumab therapy  Monitor for symptoms every 2 to 3 days  Consider Pulmonary and Infectious Disease consults | | Re-assess at least every 3 weeks.  If worsens:  Treat as Grade 2 or Grade 3 to 4. | | | |
| **Grade 2**  Mild to moderate new symptoms | | | Withhold avelumab therapy.  Pulmonary and Infectious Disease consults.  Monitor symptoms daily; consider hospitalization  1.0 to 2.0 mg/kg/day prednisone or equivalent  Add prophylactic antibiotics for opportunistic infections  Consider bronchoscopy, lung biopsy | | Re-assess every 1 to 3 days.  If improves:  When symptoms return to Grade ≤ 1, taper steroids over at least 1 month, and then resume avelumab therapy following steroids taper.  If not improving after 2 weeks or worsening:  Treat as Grade 3 to 4. | | | |
| **Grade 3 to 4**  Grade 3: Severe new symptoms; New/worsening hypoxia;  Grade 4: Life-threatening | | | Permanently discontinue avelumab therapy.  Hospitalize.  Pulmonary and Infectious Disease consults.  1.0 to 2.0 mg/kg/day prednisone or equivalent  Add prophylactic antibiotics for opportunistic infections  Consider bronchoscopy, lung biopsy | | If improves to Grade ≤ 1:  Taper steroids over at least 1 month.  If not improving after 48 hours or worsening:  Add additional immunosuppression (for example, infliximab, cyclophosphamide, IV immunoglobulin, or mycophenolate mofetil) | | | |
| Hepatic irAEs | | | | | | | | |
| Grade of Liver Test Elevation  (NCI-CTCAE v4) | | | Initial Management | | Follow-up Management | | | |
| **Grade 1**  Grade 1 AST or ALT > ULN to 3.0 x ULN and/or Total bilirubin > ULN to 1.5 x ULN | | | Continue avelumab therapy. | | Continue liver function monitoring  If worsens:  Treat as Grade 2 or 3 to 4. | | | |
| **Grade 2**  AST or ALT > 3.0 to ≤ 5 x ULN and/or total bilirubin > 1.5 to ≤ 3 x ULN | | | Withhold avelumab therapy.  Increase frequency of monitoring to every 3 days. | | If returns to Grade ≤ 1:  Resume routine monitoring; resume avelumab therapy.  If elevation persists > 5 to 7 days or worsens:  Treat as Grade 3 to 4. | | | |
| **Grade 3 to 4**  AST or ALT > 5 x ULN and/or total bilirubin > 3 x ULN | | | Permanently discontinue avelumab therapy.  Increase frequency of monitoring to every 1 to 2 days.  1.0 to 2.0 mg/kg/day prednisone or equivalent.  Add prophylactic antibiotics for opportunistic infections.  Consult gastroenterologist/hepatologist.  Consider obtaining MRI/CT scan of liver and liver biopsy if clinically warranted. | | If returns to Grade ≤ 1:  Taper steroids over at least 1 month.  If does not improve in > 3 to 5 days, worsens or rebounds:  Add mycophenolate mofetil 1 gram (g) twice daily.  If no response within an additional 3 to 5 days, consider other immunosuppressants per local guidelines. | | | |
| Renal irAEs | | | | | | | | |
| Grade of Creatinine Increased (NCI‑CTCAE v4) | | | | Initial Management | | Follow-up Management | | |
| **Grade 1**  Creatinine increased > ULN to 1.5 x ULN | | | | Continue avelumab therapy. | | Continue renal function monitoring  If worsens:  Treat as Grade 2 to 3 or 4. | | |
| **Grade 2 to 3**  Creatinine increased > 1.5 and ≤ 6 x ULN | | | | Withhold avelumab therapy.  Increase frequency of monitoring to every 3 days.  1.0 to 2.0 mg/kg/day prednisone or equivalent.  Add prophylactic antibiotics for opportunistic infections.  Consider renal biopsy. | | If returns to Grade ≤1:  Taper steroids over at least 1 month, and resume avelumab therapy following steroids taper.  If worsens:  Treat as Grade 4. | | |
| **Grade 4**  Creatinine increased > 6 x ULN | | | | Permanently discontinue avelumab therapy.  Monitor creatinine daily.   1. to 2.0 mg/kg/day prednisone or equivalent.   Add prophylactic antibiotics for opportunistic infections.  Consider renal biopsy.  Nephrology consult. | | If returns to Grade ≤1:  Taper steroids over at least 1 month. | | |
| Cardiac irAEs | | | | | | | | |
| Myocarditis | Initial Management | | | | | | | Follow-up Management |
| New onset of cardiac signs or symptoms and / or new laboratory cardiac biomarker elevations (e.g. troponin, CK-MB, BNP) or cardiac imaging abnormalities suggestive of myocarditis. | Withhold avelumab therapy.  Hospitalize.  In the presence of life threatening cardiac decompensation, consider transfer to a facility experienced in advanced heart failure and arrhythmia management.  Cardiology consult to establish etiology and rule-out immune-mediated myocarditis.  Guideline based supportive treatment as per cardiology consult.*  Consider myocardial biopsy if recommended per cardiology consult. | | | | | | | If symptoms improve and immune‑mediated etiology is ruled out, re‑start avelumab therapy.  If symptoms do not improve/worsen, viral myocarditis is excluded, and immune-mediated etiology is suspected or confirmed following cardiology consult, manage as immune-mediated myocarditis. |
| Immune-mediated myocarditis | Permanently discontinue avelumab.  Guideline based supportive treatment as appropriate as per cardiology consult.*  1.0 to 2.0 mg/kg/day prednisone or equivalent  Add prophylactic antibiotics for opportunistic infections. | | | | | | | Once improving, taper steroids over at least 1 month.  If no improvement or worsening, consider additional immunosuppressants (e.g. azathioprine, cyclosporine A). |
| *Local guidelines, or e.g. ESC or AHA guidelines  ESC guidelines website: https://www.escardio.org/Guidelines/Clinical-Practice-Guidelines  AHA guidelines website: http://professional.heart.org/professional/GuidelinesStatements/searchresults.jsp?q=&y=&t=1001 | | | | | | | | |
| Endocrine irAEs | | | | | | | | |
| Endocrine Disorder | | Initial Management | | | | | Follow-up Management | |
| **Grade 1 or Grade 2 endocrinopathies (hypothyroidism, hyperthyroidism, adrenal insufficiency, type I diabetes mellitus)** | | Continue avelumab therapy.  Endocrinology consult if needed.  Start thyroid hormone replacement therapy (for hypothyroidism), anti‑thyroid treatment (for hyperthyroidism), corticosteroids (for adrenal insufficiency) or insulin (for Type I diabetes mellitus) as appropriate.  Rule-out secondary endocrinopathies (i.e. hypopituitarism / hypophysitis). | | | | | Continue hormone replacement/suppression and monitoring of endocrine function as appropriate. | |
| **Grade 3 or Grade 4 endocrinopathies (hypothyroidism, hyperthyroidism, adrenal insufficiency, type I diabetes mellitus)** | | Withhold avelumab therapy.  Consider hospitalization.  Endocrinology consult.  Start thyroid hormone replacement therapy (for hypothyroidism), anti‑thyroid treatment (for hyperthyroidism), corticosteroids (for adrenal insufficiency) or insulin (for type I diabetes mellitus) as appropriate.  Rule-out secondary endocrinopathies (i.e. hypopituitarism / hypophysitis). | | | | | Resume avelumab once symptoms and/or laboratory tests improve to Grade ≤ 1 (with or without hormone replacement/suppression).  Continue hormone replacement/suppression and monitoring of endocrine function as appropriate. | |
| **Hypopituitarism/Hypophysitis (secondary endocrinopathies)** | | If secondary thyroid and/or adrenal insufficiency is confirmed (i.e. subnormal serum FT4 with inappropriately low TSH and/or low serum cortisol with inappropriately low ACTH) :   - Refer to endocrinologist for dynamic testing as indicated and measurement of other hormones (FSH, LH, GH/IGF-1, PRL, testosterone in men, estrogens in women) - Hormone replacement/suppressive therapy as appropriate - Perform pituitary MRI and visual field examination as indicated   **If hypophysitis confirmed:**   - Continue avelumab if mild symptoms with normal MRI. Repeat the MRI in 1 month - Withhold avelumab if moderate, severe or life-threatening symptoms of hypophysitis and/or abnormal MRI. Consider hospitalization. Initiate corticosteroids (1 to 2 mg/kg/day prednisone or equivalent) followed by corticosteroids taper during at least 1 month. - Add prophylactic antibiotics for opportunistic infections. | | | | | Resume avelumab once symptoms and hormone tests improve to Grade ≤ 1 (with or without hormone replacement).  In addition, for hypophysitis with abnormal MRI, resume avelumab only once shrinkage of the pituitary gland on MRI/CT scan is documented.  Continue hormone replacement/suppression therapy as appropriate. | |
| **Other irAEs (not described above)** | | | | | | | | |
| **Grade of other irAEs (NCI‑CTCAE v4)** | | **Initial Management** | | | | | **Follow-up Management** | |
| **Grade 2 or Grade 3 clinical signs or symptoms suggestive of a potential irAE** | | Withhold avelumab therapy pending clinical investigation | | | | | If irAE is ruled out, manage as appropriate according to the diagnosis and consider re-starting avelumab therapy.  If irAE is confirmed, treat as Grade 2 or 3 irAE. | |
| **Grade 2 irAE or first occurrence of Grade 3 irAE** | | Withhold avelumab therapy  1.0 to 2.0 mg/kg/day prednisone or equivalent  Add prophylactic antibiotics for opportunistic infections  Specialty consult as appropriate | | | | | If improves to Grade ≤ 1:  Taper steroids over at least 1 month and resume avelumab therapy following steroids taper. | |
| **Recurrence of same Grade 3 irAEs** | | Permanently discontinue avelumab therapy   1. to 2.0 mg/kg/day   prednisone or equivalent  Add prophylactic antibiotics for opportunistic infections  Specialty consult as appropriate | | | | | If improves to Grade ≤ 1:  Taper steroids over at least 1 month. | |
| **Grade 4** | | Permanently discontinue avelumab therapy   1. to 2.0 mg/kg/day   prednisone or equivalent and/or other immunosuppressant as needed  Add prophylactic antibiotics for opportunistic infections  Specialty consult. | | | | | If improves to Grade ≤ 1:  Taper steroids over at least 1 month. | |
| **Requirement for 10 mg per day or greater prednisone or equivalent for more than 12 weeks for reasons other than hormonal replacement for adrenal insufficiency**  **Persistent Grade 2 or 3 irAE lasting 12 weeks or longer** | | Permanently discontinue avelumab therapy  Specialty consult | | | | |  | |

Abbreviations: ACTH=Adrenocorticotropic Hormone; ADL=Activities of Daily Living; ALT=Alanine Aminotransferase; AST=Aspartate Aminotransferase; BNP=B-type Natriuretic Peptide; CK-MB=Creatine kinase MB; CT=Computed Tomography; FSH=Follicle-Stimulating Hormone; GH=Growth Hormone; IGF-1=Insulin-Like Growth Factor 1; irAE=Immune-Related Adverse Event; IV=Intravenous; LH=Luteinizing hormone; MRI=Magnetic Resonance Imaging; NCI-CTCAE=National Cancer Institute-Common Terminology Criteria for Adverse Events; PRL=Prolactin; T4=Thyroxine; TSH=Thyroid Stimulating Hormone; ULN=Upper Limit of Normal.

For additional information on IMP dose modifications, see IB of avelumab (version 7 of 31 March 2017) and Merck safety related items to be included in the investigator sponsored study protocols for avelumab.

Given the investigational nature of the IMP and in order to provide subjects with the maximum level of safety in case of unexpected events, the following requirements must be fulfilled before any administration to the subjects:

 Immediate access to appropriate resuscitative equipment/treatment (epinephrine, oxygen…) must be secured.

 Properly qualified and trained medical personnel must be present.

### Misuse / overdose

Any IMP misuse/or overdose associated or not with any AE should be reported on an Adverse Event form and faxed or emailed to the Principal Investigator (see Section 10.5.4).

## IMP accountability

The Principal Investigator will provide specific forms for drug accountability, which will be kept up-to-date by the Investigator and/or Hospital Pharmacist throughout the study.

The accountability forms should be filed in the ISF*.* The CRA will verify the drug accountability forms for completeness and accuracy at each site visit.

At the end of the study, delivery records should be reconciled with dispensing and used/unused records. Any discrepancy must be accounted for.

Used/unused IMP should be kept in a secure place at the site until the records have been verified by the CRA.

## Randomization codes and procedures for blinding

Not applicable.

# PRIOR AND CONCOMITANT MEDICATION

## Prior medication

The term ‘prior medication’ refers to any medication given before study entry, i.e. before informed consent signature.

All relevant prior medication taken within 30 days before inclusion must be recorded in the patient’s medical records and documented on the appropriate pages of the eCRF.

Non-inclusion criteria related to prior medication are:

1. Prior radiotherapy or chemotherapy.
2. Prior vaccinations and immunosuppressants within 30 days prior to starting the study
3. Concurrent treatment with a non-permitted drug.

All authorized medications taken regularly at baseline will be continued and documented as a concomitant medication throughout the study protocol.

In order to mitigate infusion‑related reactions, a premedication with an antihistamine and with paracetamol (acetaminophen) 30 to 60 minutes prior to the first 4 infusions of avelumab is mandatory (for example, 25‑50 mg diphenhydramine and 500‑650 mg paracetamol IV or oral).

Premedication should be administered for subsequent avelumab infusions based upon clinical judgment and presence/severity of prior infusion reactions. This may be modified based on local treatment standards and guidelines, as appropriate.

Avelumab should be administered in a setting that allows for immediate access to an intensive care unit or equivalent environment and administration of therapy for anaphylaxis, such as the ability to implement immediate resuscitation measures. Steroids (dexamethasone 10 mg), epinephrine (1:1,000 dilution), allergy medications (IV antihistamines), bronchodilators, or equivalents, and oxygen should be available for immediate access.

Following avelumab infusions, patients must be observed for 30 minutes post‑infusion for potential infusion‑related reactions.

## Concomitant medication

The term ‘concomitant medication’ refers to any medication that the patient receives at any time during the study, i.e. from study entry to the last study visit. This includes the screening/baseline period, treatment period and follow-up as defined in the protocol.

Concomitant medication should be avoided as much as possible during the study. However, if it is considered to be necessary for the patient’s welfare or well-being and is unlikely to interfere with the study assessments, it may be given at the discretion of the Investigator, preferably after consultation with the Principal Investigator. The decision to withdraw a patient on the basis of concomitant medication should preferably be made jointly by the Principal Investigator and the Investigator.

The following treatments will be more specifically tracked in the eCRF and will be administered in accordance with the following recommendations:

- Anti-emetics
- Week 3 (D15) ± 3 days – start of mFOLFOX-6 chemotherapy protocol
  - Oxaliplatin 85 mg/m^2^ in a 2-hour infusion
  - Leucovorin 400 mg/m^2^ over 2 hours
  - 48-hour infusion of fluorouracil 2,400 mg/m²
- mFOLFOX-6 chemotherapy protocol is given with avelumab in a successive manner such that mFOLFOX is administered 30-minutes after avelumab has already been administered every 2 weeks ± 3 days (weeks 3, 5, 7, 9, 11 and 13)

All concomitant medications must be recorded in the patient’s medical records and documented in the appropriate pages of the eCRF

# STUDY PLAN

The method for assessing each study parameters is described in Section 10. .

Investigators may also refer to the study flowchart (Table 1).

## 8.1. Patient recruitment

### Informed Consent

The Investigator should provide each patient with relevant, comprehensive, verbal and written information regarding the objectives and procedures of the study as well as the possible risks involved. A patient information sheet will be given to the patient.

The patient should have enough time and opportunity to inquire about study details. All his/her questions should be answered in a clear manner. The patient must be informed about his/her right to withdraw from the study at any time.

Signed informed consent must be obtained from the patient prior to undertaking any study-related procedure. If possible, informed consent must be obtained from the patient.

Two original copies of the informed consent form should be signed and dated by the patient (or the designated person as described above) and the Investigator. One original fully signed copy should be given to the patient (or the designated person as described above). The Investigator will keep the other copy. The process for obtaining consent will be documented in the patient’s file.

### Patient enrolment

The patient is considered as enrolled when informed consent has been signed by the patient.

### Patient allocation

All patients fulfilling all inclusion and none of the exclusion criteria will be considered as eligible.

Eligible patients will receive a short-course radiation therapy followed by 6 cycles of mFOLFOX-6 chemotherapy plus avelumab every 2 weeks ± 3 days. Two to three weeks after the last cycle of chemotherapy plus avelumab, the patient will undergo a TME, depending on the patient’s condition and laboratory exams.

Study treatment units will be allocated sequentially in chronological order of inclusion in ascending order.

### Replacement of early withdrawals

No replacement of early withdrawal will be performed.

## 8.2. Schedule of visits

The study flowchart (Table 1) lists all of the assessments and indicates with an “X”, the visits when they are performed. All data obtained from these assessments must be supported in the patient’s source documentation.

After enrolment, all patients will be treated with IMP. The start of radiation therapy corresponds to D1 (week 1), and IMP administration at D15 ± 3 days (week 3).

During all visits, all relevant data are to be collected in the eCRF.

### 8.2.1. Selection/patient recruitment: visit 1

Patients who fulfil the eligibility criteria will be informed of all details concerning the study. Only those who agree to participate in the study and sign the informed consent will be enrolled.

Study-related procedures such as IMP administration can be only undertaken after the obtainment of the informed consent.

The visit procedures are as per the following points:

- - 1. Informed consent
    2. Verification of selection criteria
    3. Patient number:
    - Each patient in the study is uniquely identified by a 6-digit patient number which is a combination of his/her 3-digit center number and 3-digit subject number.
    - The center number is assigned by the Principal Investigator to the investigative site.
    - Upon signing the informed consent form, the patient is assigned a patient number by the Investigator.
    - At each site, the first patient is assigned patient number 1, and subsequent patients are assigned consecutive numbers (e.g. the second patient is assigned patient number 2; the third patient is assigned patient number 3).
    1. Medical history
    2. Physical examination/vital signs/weight
    3. Laboratory tests:
    - Hematology assessments: complete blood count (CBC)
    - Blood chemistry: blood urea nitrogen (BUN), creatinine, electrolytes, SGPT, SGOT, GGT, alkaline phosphatase, bilirubin
    - Virology:
      - - HBV surface antigen and HCV antibodies
        - Conduct of HCV PCR for patients positive for HCV antibodies (to differentiate active infection from past infection with HCV)
    - Urine or serum pregnancy test for women of childbearing potential who are sexually active
    - Free T4 and TSH
    - Tumor markers (CEA and CA 19-9)
    1. Baseline biopsy to evaluate:
    - PD-L1 expression:
      - - Will be evaluated on tumor cells and infiltrating immune T cells.
        - Will be classified as negative or positive or not applicable (categorical variable).
    - CD4+, CD8+ and CD3+ T cell infiltration:
      - - Will be quantified in mm^2^ in the most abundant tumor-infiltrating area in both, the stroma and the tumor, of the baseline biopsy (continuous variable).
        - After the recruitment of around 5 patients, a cut-off value will be agreed upon and the numerical values corresponding to T cell infiltration (continuous variable) will then be classified as low or high or not applicable (categorical variable).
    - Microsatellite instability (MSI or MMR status):
      - - Will be evaluated once on either the baseline biopsy or D10 biopsy.
        - The MSI predictive markers to be assessed are: MLH-1, MSH-2, MSH-6, and PMS-2.
    1. Prior treatment (within the 30 previous days).
    2. Imaging: pelvic MRI (rectal protocol), CT or PET scan of the chest and the abdomen
    3. Patient-reported outcome: quality of life assessment using FACT-C questionnaire (Lynch BM et al., 2008):
    - The questionnaire is available in Arabic and English versions.
    - It should be completed by the patient in Arabic, or in English in case he/she does not understand Arabic.
    - Once completed, the site staff will do the data entry in the eCRF.
    - The average time to complete the questionnaire is 5 to10 minutes.
    - FACT-C is a 36-item quality-of-life questionnaire with five subscales: physical well-being (PWB), social/family well-being (SWB), emotional well-being (EWB), and functional well-being (FWB), and colorectal cancer-specific additional concerns (colorectal cancer subscale, CCS).
    - Participants rate how they have felt over the past 7 days, on a 5-point Likert scale ranging from 0 (not at all) to 4 (very much).
    - The total FACT-C score is obtained by summing individual subscale scores (PWB + EWB + SWB + FWB + CCS).
    - The overall quality-of-life score can range from 0 to 136 (two items relevant only to stoma patients are not included in the score).
    - Higher scores indicate better quality of life.
    - Each subscale has a maximum score of 28, except for the emotional well-being subscale, which has a maximum of 24.

### 8.2.2. Radiation therapy period: visits 2 to 7

The procedures to be done for 5 days from D1 to D5 during week 1 (visits 2 to 6) are as follows:

- - 1. Short-course radiation therapy:
    - **Treatment technique:**
      - Either 3D conformal or intensity-modulated radiotherapy (IMRT) treatment planning may be used on this study.
      - Both techniques are approved, so either technique may be used.
      - Fields should be chosen to minimize dose to organs at risk (small bowel, femoral heads, and bladder).
      - Multiple beam techniques, both coplanar and non-coplanar may be used to achieve the objective.
      - CT with immobilization is required for planning.
      - Patients are to be placed in a position that best suits the technique used and ensures immobilization and displacement of normal tissues.
      - The slice thickness must be no greater than 5 mm, and the Gross Tumor Volume (GTV), Clinical Target Volume (CTV), and Planning Target Volume (PTV) must be defined on all axial CT slices.
      - Data on radiation technique and parameters should be made available for review at the beginning or completion of radiation therapy.
    - **Equipment:**
      - High energy X-rays with nominal energy of 6 MV (IMRT) or 15 MV (3D) should be used.
      - Image guidance (cone beam) should be used on daily basis.
    - **Target volume definitions.**
      - Volume definition and contouring will be done on planning CT scans.
      - Use of contrast enhanced MRI for better target delineation is encouraged.
      - GTV must include all primary tumor extent, and enlarged lymph nodes.
      - CTV includes GTV with 0.5 cm extension and all perirectal, presacral, and internal iliac lymph nodes all the way up to the sacral promontory.
      - PTV is a 1cm expansion of the CTV in all directions.
      - Adjustments of PTV anteriorly could be made to reduce incidental radiation to the bladder and bowel.
    - **Target dose:**
      - The daily dose will be 5 Gy to a total dose of 25 Gy.
      - Dose will be prescribed to an isodose surface that encompasses the PTV and allows the dose uniformity requirements of ≤5%.
      - Dose is specified in Gy to muscle.
      - Density corrections are required as all patients will be treated using CT-based planning.
    - All radiation plans should be submitted for audit to AUBMC.
    1. Adverse event collection

The procedures to be done at D10 during week 2 ± 3 days (visit 7) are as follows:

- - 1. Sigmoidoscopy
    2. Biopsy to evaluate:
    - PD-L1 expression:
      - - Will be evaluated on tumor cells and infiltrating immune T cells.
        - Will be classified as negative or positive or not applicable (categorical variable).
    - CD4+, CD8+ and CD3+ T cell infiltration:
      - - Will be quantified in mm^2^ in the most abundant tumor-infiltrating area in both, the stroma and the tumor, of the D10 biopsy (continuous variable).
        - After the recruitment of around 5 patients, a cut-off value will be agreed upon and the numerical values corresponding to T cell infiltration (continuous variable) will then be classified as low or high or not applicable (categorical variable).
    - Microsatellite instability (MSI or MMR status):
      - - Will be evaluated once on either the baseline biopsy or D10 biopsy.
        - The MSI predictive markers to be assessed are: MLH-1, MSH-2, MSH-6, and PMS-2.
    - Image of the biopsy is to be provided.
    1. Concomitant medications
    2. Adverse event collection

### 8.2.3. Treatment period: visits 8 to 13

The procedures to be done from week 3 ± 3 days to week 13 ± 3 days (visits 8 to 14), starting D15 are as follows:

- - 1. Physical examination/vital signs/weight
    2. Laboratory tests:
    - Hematology assessments: CBC
    - Blood chemistry: BUN, creatinine, electrolytes, SGPT, SGOT, GGT, alkaline phosphatase, bilirubin
    - Urine or serum pregnancy test for women of childbearing potential who are sexually active
    - Free T4 and TSH
    1. Concomitant medications including mFOLFOX-6 chemotherapy protocol and anti-emetics:
    - Oxaliplatin 85 mg/m^2^ in a 2-hour infusion
    - Leucovorin 400 mg/m^2^ over 2 hours
    - 48-hour infusion of fluorouracil 2,400 mg/m^2^
    1. IMP administration: avelumab 10 mg/kg first administration at D15, then every 2 weeks for 6 cycles
    2. Adverse event collection
    3. Patient-reported outcome: quality of life assessment using FACT-C questionnaire

### 8.2.4. End of treatment visit including study completion and premature withdrawal: visit 13

Besides the procedures to be done at week 13 ± 3 days, tumor markers (CEA and CA 19-9) should be assessed at the end of treatment. Also, at the time patients discontinue the study treatment, a visit should be scheduled as soon as possible, but no later than 14 days from the last day of study medication, at which time all of the assessments listed for the End of Treatment visit will be performed. An End of Treatment eCRF page should be completed, giving the date and reason for stopping the study treatment.

### 8.2.5. Surgery: visit 14

Depending on the patient’s condition and laboratory exams, the procedures to be done 2 to 3 weeks ± 3 days after the last cycle of chemotherapy plus avelumab are as follows:

- - 1. Prior to surgery: pelvic MRI (rectal protocol), optional
    2. Frequency, grade, and attribution of surgical complications to the neoadjuvant treatment using the Clavien-Dindo classification (to grade surgical complications).
    3. Laboratory tests:
    - Hematology assessments: CBC
    - Coagulation tests (PT and PTT) should be done a couple of days prior to surgery on week 16 or 17
    - Blood chemistry: BUN, creatinine, electrolytes, SGPT, SGOT, GGT, alkaline phosphatase, bilirubin
    - Urine or serum pregnancy test for women of childbearing potential who are sexually active
    1. Concomitant medications
    2. Adverse event collection
    3. Surgery: TME (open, laparoscopic, or robotic):
    - All TME procedures will be video recorded and the corresponding videotapes and images of the resected specimens are to be made available.
    - Centralized random audit of videos will be performed (Curtis NJ et al., 2017).
    1. Quality assessment and completeness in a TME specimen: all TME specimens are to be processed and graded using the recommendations of the College of American Pathologists (CAP, 2013; Parfitt JR et al., 2007).
    - Specimen handling upon receiving:
      - Paint the non-peritonealized bare areas of the specimen with ink.
      - Open the specimen along the anterior aspect from the top and the bottom, leaving the bowel intact at a level just above and just below the tumor.
      - Place loose, formalin-soaked gauze wicks into the unopened ends of the bowel.
      - Fix the specimen for at least 48 hours.
    - Fixed specimen:
      - Slice through the unopened rectum at 3–5 mm intervals; lay slices down on the work surface.
      - Inspect these slices to note:
        - Extent of tumor and the closest distance of tumor to the CRM (circumferential radial margin, record this distance).
        - Any obviously positive nodes and the distance of any positive node to the CRM (record this distance).
        - Record whether the closest distance of tumor to CRM is anterior, posterior or lateral.
        - Fat away from the tumor must also be examined to detect lymph nodes.
    - Block selection for microscopic examination:
      - Three blocks of tumor showing closest CRM.
      - Two blocks of tumor showing luminal aspect.
      - All lymph nodes (being careful not to double-count nodes present in more than one slice).
      - Any polyps.
      - Proximal and distal resection margins (distal margin includes both mucosa and mesorectum; blocks from mucosal margins may be omitted if tumor is greater than 3 cm away).
    - Completeness of the mesorectum in a total mesorectal excision specimen, as per the criteria of the College of American Pathologists (CAP, 2013):
      - The specimen should be assessed grossly to assess the completeness of the surgery according to the below definition:
      - Complete: Mesorectum is intact and smooth and any defect in surface is not deeper than 5 mm. CRM is smooth and regular.
      - Nearly complete: The mesorectum is irregular however no muscularis propria is visible. CRM is irregular.
      - Incomplete: The mesorectum is little bulk with defects reaching muscularis propria. CRM is irregular.
    1. After the surgery: documentation of whether the patient achieved a pathological complete response defined as no viable tumor cells on the resected specimen.

9) Resected tumor specimen to evaluate:

- - - PD-L1 expression:
      - - Will be evaluated on tumor cells and infiltrating immune T cells.
        - Will be classified as negative or positive or not applicable (categorical variable).
    - CD4+, CD8+ and CD3+ T cell infiltration:
      - - Will be quantified in mm^2^ in the most abundant tumor-infiltrating area in both, the stroma and the tumor, of the surgical specimen (continuous variable).
        - In case of pathologic complete response, the T cell count corresponding to the tumor area of the surgical specimen will be noted as “not applicable”.
        - After the recruitment of around 5 patients, a cut-off value will be agreed upon and the numerical values corresponding to T cell infiltration (continuous variable) will then be classified as low or high or not applicable (categorical variable).
    - TRG:

The Becker et al. tumor regression grading system will be used to categorize the amount of regressive changes after cytotoxic treatment as follows:1a. No viable tumor/ tumor bed + chemotherapy effect;

1b. <10% viable tumor/ tumor bed + chemotherapy effect;

2. 10-50% viable tumor/ tumor bed + chemotherapy effect;

3. >50% viable tumor/ tumor bed **±** chemotherapy effect.

### 8.2.6. Follow-up visits: every 3 months after surgery

The procedures to be done every 3 months for 3 years after surgery are as follows:

- - 1. Physical examination/vital signs/weight
    2. Laboratory tests:
    - Tumor markers (CEA and CA 19-9)
    1. Concomitant medications
    2. Adverse event collection
    3. Patient-reported outcome: quality of life assessment using FACT-C questionnaire
    4. Patient survival status
    5. Disease Status

### 8.2.7. End of study visit (last patient visit / last patient contact)

This visit and procedures also apply to patients who are prematurely withdrawn.

The last patient visit will be done at 3 years after the surgery, the visit procedures are as follows:

- - 1. Physical examination/vital signs/weight
    2. Laboratory tests:
    - Tumor markers (CEA and CA 19-9)
    1. Concomitant medications
    2. Adverse event collection
    3. Patient-reported outcome: quality of life assessment using FACT-C questionnaire
    4. Patient survival status
    5. Disease Status

## 8.3. Assessments

### 8.3.1. Assessment performed at site

- - 1. Clinical assessments

| **Clinical assessment** | **Time of assessment** |
| --- | --- |
| Routine monitoring of vital signs (heart rate, blood pressure, and body temperature) | At inclusion  Every 2 weeks ± 3 days during chemotherapy  Every 3 months ± 3 days after surgery for 3 years |
| Weight |
| Physical examinations: total body examination (i.e., general appearance, skin, neck, including thyroid, eyes, ears, nose, throat, lungs, heart, abdomen, back, lymph nodes and extremities) and a clinical neurological examination |
| Pelvic MRI (rectal protocol) | At inclusion  Prior to surgery (optional) |
| CT or PET scan of the chest and the abdomen | At inclusion |

- - 1. Laboratory assessment:

| **Blood assessments** | **Volume of blood sample** | **Type of tube** | **Time of assessment** |
| --- | --- | --- | --- |
| CBC | 10 mL | EDTA | Prior to study initiation  Start of chemotherapy  Then every two weeks ± 3 days  Then prior to surgery |
| SGPT, SGOT, GGT, alkaline phosphatase, bilirubin electrolytes, creatinine, BUN | 10 mL | Regular (no anti-coagulants) |  |
| Tumor markers (CEA and CA 19-9) |  |  | At inclusion  End of treatment  During follow-up visits  End of study |
| Free T4 and TSH |  |  | At inclusion  At least every 8 weeks during treatment  At end of treatment or 30 days post-treatment safety follow-up |
| Virology | 5 mL |  | At inclusion |

All biological parameters will be determined locally.

- - 1. Urine or serum pregnancy test:
       - For women of childbearing potential and who are sexually active must be performed at baseline and least every month during treatment.
       - It should be negative when the patient is enrolled in the study.
    2. Given the potential risk for delayed immune-related toxicities, safety follow-up must be performed up to 90 days after the last dose of avelumab administration.
    3. The extended safety follow-up beyond 30 days after last avelumab administration may be performed either via a site visit or via a telephone call with subsequent site visit requested in case any concerns noted during the telephone call.

### 8.3.2. Centralized assessments

- - 1. Pathology assessment:

| **Assessment** | **Time of assessment** |
| --- | --- |
| PD-L1 expression on tumor and infiltrating immune cells & CD4+, CD8+ and CD3+ T cell infiltration | Baseline biopsy  D10  and  Surgical resection specimen |
| MSI (MMR status) | Once on either the baseline biopsy  or  D10 biopsy |
| TRG | Resected specimen |

Details about the samples preparation and the shipment to the central lab (at AUBMC) are provided in the lab manual.

## 8.4 Compliance with the study plan

The Investigator should make every effort to comply with the study plan. If the Investigator encounters difficulties in complying with the study plan, e.g. with regard to the schedule of visits or the required procedures, he/she must alert the CRA. The Principal Investigator may consider it relevant to issue an amendment.

The Investigator should make every effort to avoid the occurrence of deviations. If deviations occur or if the Investigator knows that a deviation will occur, he/she must promptly inform the CRA to determine how to manage the deviation.

# SAFETY

## Safety reference document

In this study, the IB of the IMP (version 7 of 31 March 2017), applicable at the time of an AE occurrence, will be considered as the Safety Reference Document, based on which evaluation of the AE will be performed, in particular regarding expectedness, causality, severity and outcome.

## Benefit / risk information

### 9.2.1. Expected risk(s) related to the IMP(s)

The following adverse reaction met the threshold of causal association (based on comprehensive medical evaluation considering the frequency, mechanism of action and temporal relationship after excluding other possible etiologies) defined by the Principal Investigator.

The Adverse Reactions for avelumab are presented in Tables 4 and 5 by SOC and PTs based on the experience in avelumab clinical studies.

The adverse reactions include those reported with a frequency ≥ 10% in the single arm trials for AEs observed in the pooled safety dataset of 1,738 patients treated with avelumab in various solid tumors (studies EMR100070-001 and EMR100070-003) or those adverse reactions that following medical evaluation met the pre-specified criteria to be classified as an immune-related AEs or an infusion-related reaction.

Adverse reactions will be considered as expected, unless nature, severity, specificity, or outcome is not consistent with the one previously observed. Due to request received from some European Health Authorities, adverse reactions with a single or no serious adverse event (SAE) reported with a fatal outcome will be reported in an expedited manner to the respective HAs.

The following definitions apply to the frequency terminology used hereafter:

- - - - Very common (≥ 1/10)
      - Common (≥ 1/100 < 1/10)
      - Uncommon (≥ 1/1,000 < 1/100)
      - Rare (≥ 1/10,000 < 1/1,000)
      - Very rare (< 1/10,000)

Table 4 Expected adverse reactions in patients treated with avelumab in clinical studies


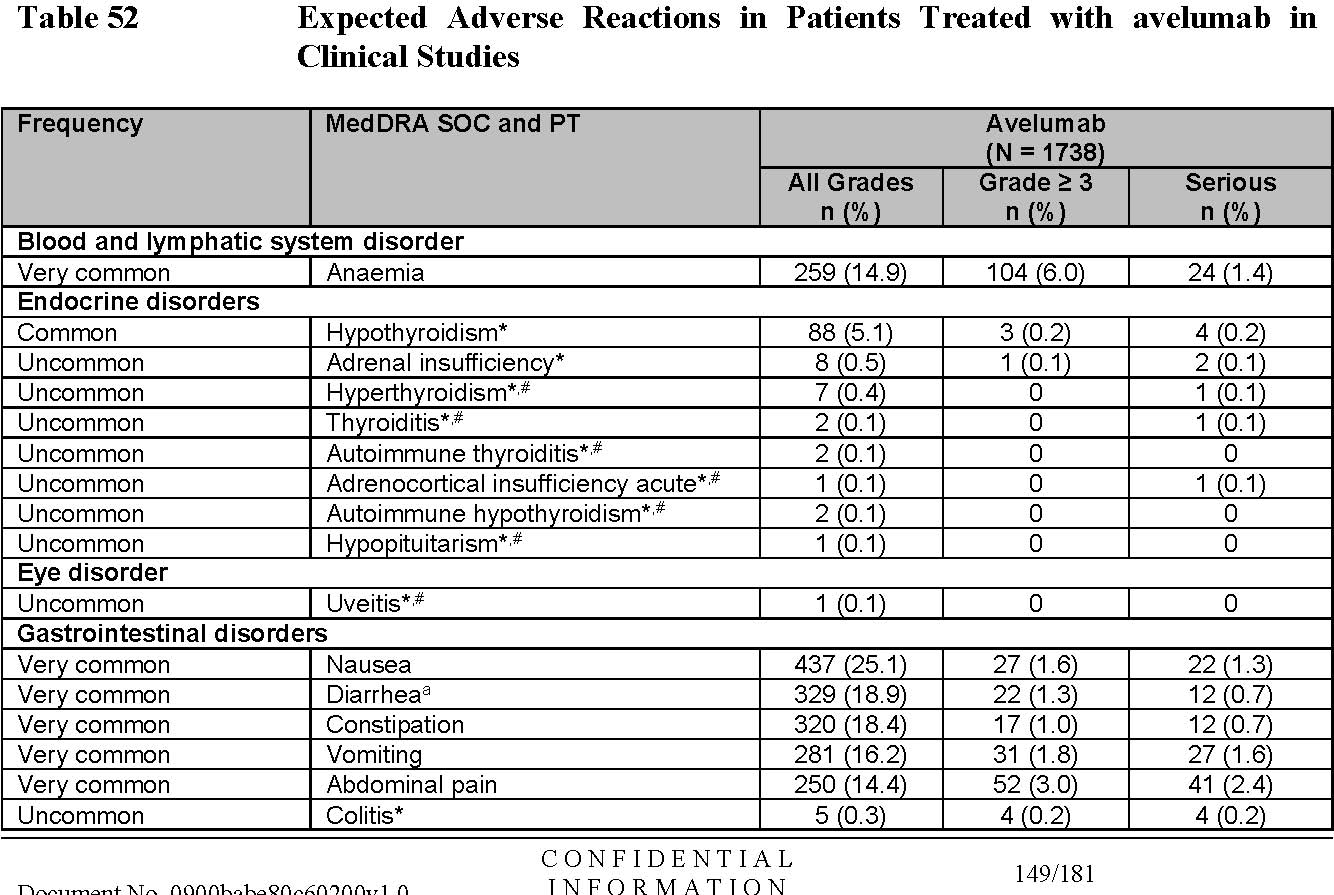


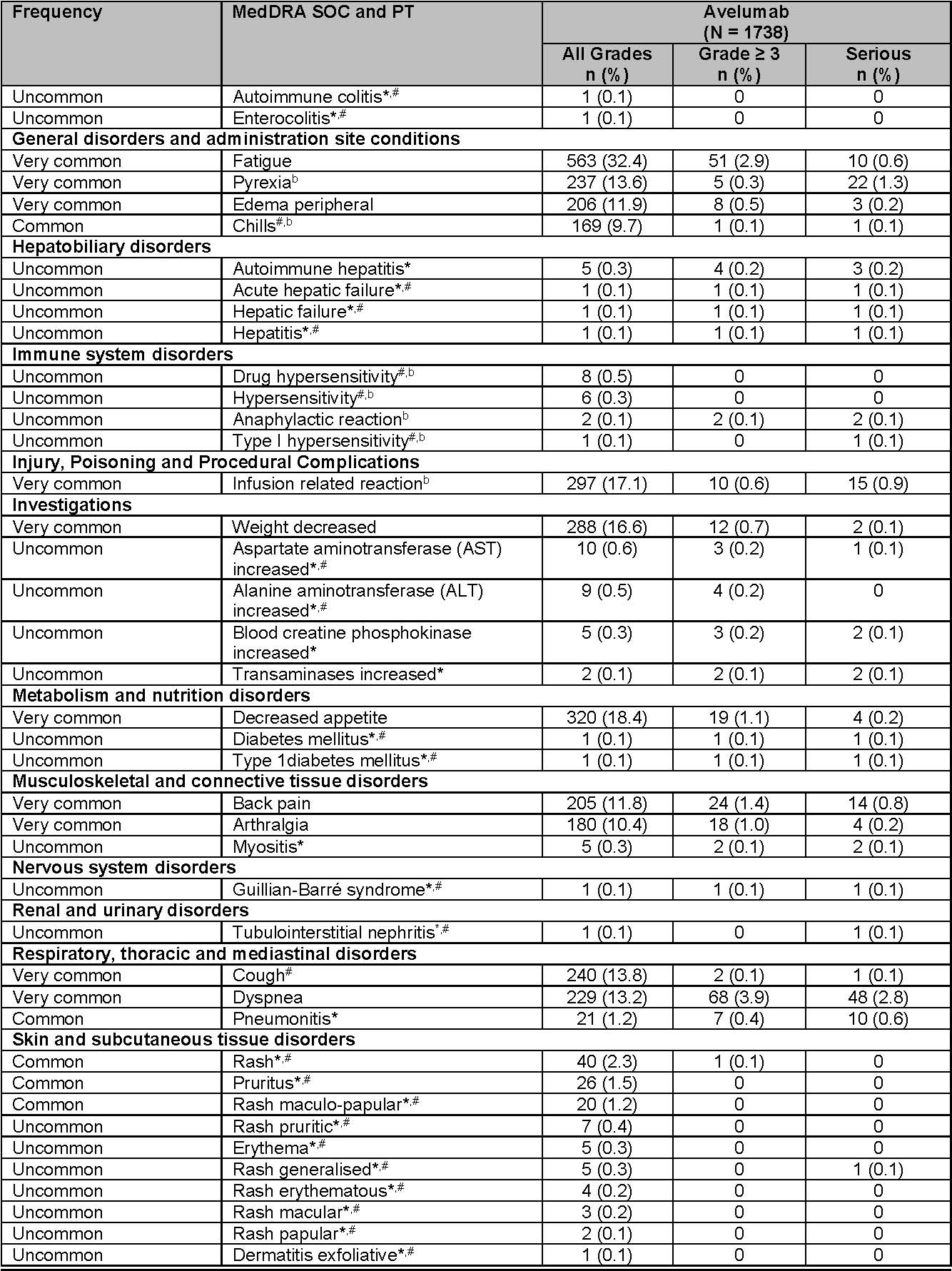

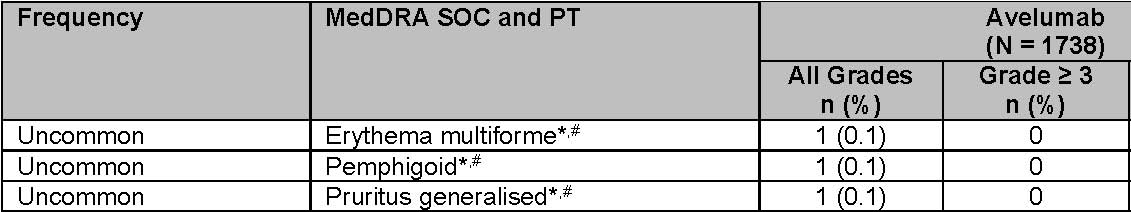


Abbreviations: MedDRA=Medical Dictionary for Regulatory Activities; n=number; PT=Preferred Term; SOC=System Organ and Class.

* Immune-related adverse reaction based on medical review

# Single or no SAEs, will be reported in an expedited manner to European Health Authorities as requested

a Frequencies presented in the table represent all events of diarrhea (all causalities) including immune-related diarrhea: 21 (1.2%); Grade ≥ 3 4 (0.2%); serious 4 (0.2%).

b Frequencies presented in the table represent all events (all causalities) including infusion-related adverse reaction based on predefined definition.

Pyrexia: 62 (3.6%); Grade ≥ 3 0 (0%), serious 3 (0.2%)

Chills: 94 (5.4%); Grade ≥ 3 0 (0%), serious 1 (0.1%)

Infusion related reaction: 296 (17.0%), Grade ≥ 10 (0.6%), serious 15 (0.9%)

Drug hypersensitivity: 5 (0.3%), Grade ≥ 3 0 (0%), serious 0 (0%)

Hypersensitivity: 3 (0.2%), Grade ≥ 3 0 (0%), serious 0 (0%)

Anaphylactic reaction: 1 (0.1%), Grade ≥ 3 1 (0.1%), serious 1 (0.1%)

Type I hypersensitivity: (0.1%), Grade ≥ 3 0 (0%), serious 1 (0.1%).

**Table 5** Other expected adverse reactions reported in the clinical avelumab program outside the pooled safety dataset

| MedDRA SOC and PT |  | Avelumab |  |
| --- | --- | --- | --- |
|  | Number of | Overall | Serious |
|  | patients studied | n | n |
|  | n |  |  |
| **Cardiac disorders** |  |  |  |
| Myocarditis* | 2600 | 2 | 2 |
| **Immune system disorders** |  |  |  |
| Graft versus host disease in liver** | 29 | 2 | 2 |
| Graft versus host disease in skin** | 29 | 1 | 1 |

* Immune-related adverse reaction based on medical review

** In the Hodgkin lymphoma study population

Abbreviations: MedDRA=Medical Dictionary for Regulatory Activities; n=number; PT=preferred term; SOC=System Organ and Class.

Adverse reactions with observed fatal outcome in the avelumab clinical development programs included: immune-related pneumonitis, immune-related hepatitis, immune-related myocarditis.

**Immunogenicity**

Of 1,738 patients treated with avelumab 10 mg/kg as an intravenous infusion every 2 weeks ± 3 days, 1,558 were evaluable for treatment-emergent anti-drug antibodies (ADA) and 64 (4.1%) tested positive. The development of treatment-emergent ADA against avelumab did not appear to alter the pharmacokinetic profile or risk of infusion-related reactions.

### 9.2.2. Other expected risk(s)

**Related to Non-IMP: Adverse events and toxicity grading for mFOLFOX-6 (NCI-CTCAE)**

AEs that are expected in association with the use of mFOLFOX-6 chemotherapy protocol will be assessed along with the toxicity grading using the fourth version of NCI-CTCAE (2010).

The common and less common AEs with mFOLFOX-6 are available in Table 6, and NCI-CTCAE grading for the common AEs in Table 7.

**Table 6** Common and less common adverse events with mFOLFOX-6

| **Common adverse events** | **Less common adverse events** |
| --- | --- |
| Fatigue  Neuropathy  Nausea/vomiting  Increased liver function tests  Diarrhea/constipation  Abdominal pain/anorexia  Pharyngolaryngeal dysesthesia  Mucositis  Myelosuppression ± infection  Bleeding  Rash/hand-foot syndrome/alopecia  Musculoskeletal pain/edema | Dyspepsia  Pancreatitis  Hypersensitivity  Conjunctivitis  Rhabdomyolysis  Hemolysis/hemolytic uremic syndrome  Nephrotoxicity  Pneumonitis  Arterial/venous thromboembolism  Cardiotoxicity/arrhythmia  Reversible posterior leukoencephalopathy syndrome |

**Table 7 NCI-CTCAE grading for the common adverse events**

| **Adverse Event** | **Grade 1** | **Grade 2** | **Grade 3** | **Grade 4** | **Grade 5** |
| --- | --- | --- | --- | --- | --- |
| Fatigue | Fatigue relieved by rest | Fatigue not relieved by rest; limiting instrumental ADL | Fatigue not relieved by rest, limiting self-care ADL | - | - |
| Dysesthesia/  Paresthesia | Mild sensory alteration | Moderate sensory alteration; limiting instrumental ADL | Severe sensory alteration; limiting self-care ADL | - | - |
| Peripheral sensory  neuropathy | Asymptomatic; loss of deep tendon reflexes or paresthesia | Moderate symptoms; limiting  instrumental ADL | Severe symptoms; limiting self-care ADL | Life-threatening  consequences; urgent intervention indicated | Death |
| Nausea | Loss of appetite without alteration in eating habits | Oral intake decreased without  significant weight loss, dehydration or malnutrition | Inadequate oral caloric or fluid intake; tube feeding, TPN, or hospitalization indicated | - | - |
| Vomiting | 1-2 episodes (separated by 5 minutes) in 24 hours | 3-5 episodes (separated by 5  minutes) in 24 hours | ≥6 episodes (separated by 5  minutes) in 24 hours; tube feeding, TPN or hospitalization indicated | Life-threatening  consequences; urgent intervention indicated | Death |
| Anorexia | Loss of appetite without  alteration in eating habits | Oral intake altered without  significant weight loss or  malnutrition; oral nutritional  supplements indicated | Associated with significant  weight loss or malnutrition  (e.g. inadequate oral caloric  and/or fluid intake); tube  feeding or TPN indicated | Life-threatening  consequences; urgent  intervention indicated | Death |
| Abdominal pain | Mild pain | Moderate pain; limiting instrumental ADL | Severe pain; limiting self-care ADL | - | - |
| Constipation | Occasional or intermittent symptoms; occasional use of  stool softeners, laxatives,  dietary modification, or enema | Persistent symptoms with  regular use of laxatives or  enemas; limiting instrumental  ADL | Obstipation with manual evacuation indicated; limiting  self-care ADL | Life-threatening  consequences; urgent  intervention indicated | Death |
| Diarrhea | Increase of <4 stools per day over baseline; mild increase in ostomy output compared to  baseline | Increase of 4-6 stools per day over baseline; moderate  increase in ostomy output compared to baseline | Increase of ≥7 stools per day  over baseline; incontinence;  hospitalization indicated;  severe increase in ostomy output compared to baseline; limiting self-care ADL | Life-threatening  consequences; urgent  intervention indicated | Death |
| Mucositis | Asymptomatic or mild symptoms; intervention not  indicated | Moderate pain; not interfering with oral intake; modified diet  indicated | Severe pain; interfering with  oral intake | Life-threatening  consequences; urgent  intervention indicated | Death |
| Increased bilirubin | >ULN - 1.5 x ULN | >1.5 - 3.0 x ULN | >3.0 - 10.0 x ULN | >10.0 x ULN | - |
| Increased ALT/AST | >ULN - 3.0 x ULN | >3.0 - 5.0 x ULN | >5.0 - 20.0 x ULN | >20.0 x ULN | - |
| Increased Alk Phos | >ULN - 2.5 x ULN | >2.5 - 5.0 x ULN | >5.0 - 20.0 x ULN | >20.0 x ULN | - |
| Febrile neutropenia | - | - | ANC <1,000/mm^3^ with a single temperature of >38.3ºC (101ºF) or a sustained temperature of  ≥38ºC (100.4ºF) for more than one  hour. | Life-threatening  consequences; urgent  intervention indicated | Death |
| Anemia | Hgb <LLN -10.0 g/dL; <LLN - 6.2 mmol/L;  <LLN - 100 g/L | Hgb <10.0 - 8.0 g/dL; <6.2 -4.9 mmol/L; <100 -80g/L | Hgb <8.0 g/dL;  <4.9 mmol/L;  <80 g/L;  transfusion indicated | Life-threatening  consequences; urgent  intervention indicated | Death |
| Bleeding | Mild; intervention not indicated | Moderate symptoms; medical  intervention or minor cauterization indicated | Transfusion, radiologic, endoscopic, or elective operative intervention indicated | Life-threatening  consequences; urgent  intervention indicated | Death |
| Rash | Macules/papules covering <10% BSA with or without  symptoms (e.g. pruritus, burning, tightness) | Macules/papules covering 10-30% BSA with or without symptoms (e.g. pruritus,  burning, tightness); limiting instrumental ADL | Macules/papules covering >30% BSA with or without  Associated symptoms; limiting self-care ADL | - | - |
| Alopecia | Hair loss of <50% of normal for that individual that is not obvious from a distance but only on close inspection; a different hair style may be required to cover the hair loss but it does not require a wig or hair piece to camouflage | Hair loss of ≥50% normal for that individual that is readily apparent to others; a wig or hair piece is necessary if the patient desires to completely camouflage the hair loss; associated with psychosocial impact | - | - | - |
| Hand-foot syndrome | Minimal skin changes or dermatitis (e.g. erythema, edema, or hyperkeratosis)  without pain | Skin changes (e.g. peeling, blisters, bleeding, edema, or  hyperkeratosis) with pain; limiting instrumental ADL | Severe skin changes (e.g.  peeling, blisters, bleeding, edema, or hyperkeratosis)  with pain; limiting self-care ADL | - | - |
| Edema | 5 - 10% inter-limb discrepancy in volume or circumference at  point of greatest visible difference; swelling or obscuration of anatomic  architecture on close inspection | >10 - 30% inter-limb  discrepancy in volume or  circumference at point of greatest visible difference;  readily apparent obscuration of anatomic architecture;  obliteration of skin folds; readily apparent deviation  from normal anatomic  contour; limiting instrumental ADL | >30% inter-limb discrepancy in volume; gross deviation from normal anatomic contour; limiting self care ADL | - | - |
| Myalgia | Mild pain | Moderate pain; limiting  instrumental ADL | Severe pain; limiting self care ADL | - | - |

Abbreviations: ADL=Activities of Daily Living; ALT=Alanine Aminotransferase; ANC=Absolute Neutrophil Count; AST=Aspartate Aminotransferase; BSA=Body Surface Area; Hgb=Hemoglobin; LLN=Lower Limit of Normal; NCI-CTCAE=National Cancer Institute-Common Terminology Criteria for Adverse Events; ULN=Upper Limit of Normal; TPN=Total Parenteral Nutrition.

**Related to Surgery: The Clavien-Dindo Classification of surgical complications**

- - 1. **Grade I:** Any deviation from the normal post-operative course without the need for pharmacological treatment or surgical, endoscopic and radiological interventions. Allowed therapeutic regimens are: drugs as anti-emetics, antipyretics, analgetics, diuretics and electrolytes and physiotherapy. This grade also includes wound infections opened at the bedside.
    2. **Grade II:** Requiring pharmacological treatment with drugs other than such allowed for grade I complications. Blood transfusions and total parenteral nutrition are also included.
    3. **Grade III:** Requiring surgical, endoscopic or radiological intervention.

**Grade IIIa:** intervention not under general anesthesia.

**Grade IIIb:** intervention under general anesthesia.

- - 1. **Grade IV:** Life-threatening complication (including complications of the central nervous system such as brain hemorrhage, ischemic stroke, sub-arrachnoidal bleeding, but excluding transient ischemic attacks) requiring intensive care management.

**Grade IVa:** single organ dysfunction (including dialysis).

**Grade IVb:** multi organ dysfunction.

- - 1. **Grade V:** Death.
    2. Suffix ‘d: If the patients suffers from a complication at the time of discharge, the suffix “d” (for ‘disability’) is added to the respective grade of complication. This label indicates the need for a follow-up to fully evaluate the complication.

### 9.2.3. Benefit / risk balance

Avelumab has already received [orphan drug](https://en.wikipedia.org/wiki/Orphan_drug) designation by the European Medicines Agency for the treatment of gastric cancer in January 2017. The US Food and Drug Administration (FDA) approved it on 23 March 2017 for Merkel-cell carcinoma, an aggressive type of skin cancer, under the name Bavencio® (a trademark of Merck KGaA, Darmstadt, Germany).

The aim of the present study is to evaluate the rate of pCR rate following short-course radiation then mFOLFOX-6/avelumab. The expected benefits of the proposed therapeutic strategy are:

- - 1. The addition of avelumab to mFOLFOX-6 chemotherapy after short-course radiation will increase the pathologic complete response rate for locally-advanced rectal cancer amenable to surgery compared to historical controls.
    2. The addition of avelumab to mFOLFOX-6 chemotherapy after short-course radiation will increase the 3-year progression-free survival rate for locally-advanced rectal cancer amenable to surgery compared to historical controls.
    3. The addition of avelumab to mFOLFOX-6 chemotherapy after short-course radiation will be safe and well-tolerated.

This protocol will allow the evaluation of the therapeutic relevance of the proposed strategy by reliable and clinically approved endpoints.

Infusion-related reactions including drug hypersensitivity reaction and immune-mediated adverse reactions (immune-related pneumonitis, immune-related colitis, immune-related hepatitis, immune-related endocrinopathies [thyroid disorders, adrenal insufficiency, new onset type I diabetes mellitus, pituitary disorders], immune-related nephritis and renal dysfunction and other immune-related AEs (myositis, myocarditis, Guillain-Barré syndrome, uveitis) have been identified as important risks for avelumab. However, the hypothesis of an increased risk of AEs is not highly plausible in this context. Nevertheless, this protocol will assess the safety of avelumab.

## Risk minimization actions throughout the protocol

To control possible risks related to patient’s participation in the study, mainly to IMP administration, please refer to Section 6.4. Tables 2 and 3.

## Alternative therapeutic management - emergencies handling

If allergic or anaphylactic-type of reactions occur, the infusion should be stopped immediately.

In case of abnormally persistent bleeding, medical care will be given according to local management strategies.

In case of misuse or overdose, emergency care is left to the discretion of the Investigator.

## Definition and reporting of (serious) adverse events

### Definition of adverse event and serious adverse event

**An adverse event** **(AE)** is any untoward medical occurrence in a patient or clinical investigation subject administered a medicinal product and that does not necessarily have a causal relationship with this treatment.

It can be any unfavorable sign, including an abnormal clinically significant laboratory finding or ECG, any symptom, syndrome, or disease, whether or not related to the IMP that can be new or exacerbated by the deterioration of a pre-existing condition.

**A Serious Adverse Event** **(SAE)** is an AE that, at any time, fulfils one or more of the following criteria:

1. **results in death,**
2. **is life threatening**, i.e. the patient was at immediate risk of death at the time of the event; it does NOT refer to an event which might have caused death if it were more severe,
3. **requires in-patient hospitalization or prolongation of existing hospitalization**, i.e. hospitalization signifies that the patient has been detained, usually (but not systematically) involving at least an overnight stay,
4. **results in persistent or significant disability/incapacity,** i.e. substantial disruption of a person’s ability to carry out normal life functions,
5. **is a congenital anomaly/birth defect,**
6. **is any important medical event** that may not be immediately life threatening or result in death or hospitalization but, based upon appropriate medical judgment, may endanger the patient or may require intervention to prevent one of the other outcomes listed above.

- **Occurrence of IMP overdose, abuse/misuse, or drug dependency**, whether or not clinical signs or symptoms are present, should be reported as an SAE to the Principal Investigator.
- **Surgical intervention** is not to be considered as SAE but the medical condition requiring the surgery is to be reported as such.
- **Events initially reported as an AE may become serious**. For example, diarrhea may become debilitating and require hospitalization or prolongation of hospitalization and is then reported as SAE.
- **Distinction should be made between serious and severe AEs.** Severity is a measure of intensity whereas seriousness is based on the seriousness criteria described above. For example, nausea that persists for several hours may be considered as “severe”, but not as an “SAE”. On the contrary, a cardiovascular event that causes limited degree of disability may be considered as “not severe” but would be recorded as an “SAE”.

### Period of (serious) adverse event data collection

In order to ensure complete safety data collection, recording and reporting, all (S)AEs occurring during the study, i.e. after signature of the Informed Consent, including any pre- and post-treatment periods required by the protocol, must be recorded, even if no IMP was taken.

The period of safety observation for this study is from visit 2 (D1, week 1) to 3 years after the surgery (see Study Flowchart, Table 1).

**If the Investigator detects an (S)AE after the above-defined period of observation** and considers the event as possibly related to the IMP, he/she should contact the Principal Investigator to determine how it must be documented and reported.

### Recording and description of (serious) adverse event

Apart from AEs clinically observed by the Investigator, or recorded on self-assessment forms (e.g. patient diary), the patient will be given the opportunity to report AEs spontaneously, by questioning for instance “Did you notice anything unusual about your health since your last visit?”

It is the responsibility of the Investigator to record all the relevant information regarding the event.

The guidelines and definitions for reporting (S)AE by the Investigator are described in Appendix 20.2.

The Investigator is requested to assess the relationship between the IMP and the occurrence of each (S)AE. Alternatives causes, such as the underlying diseases, concomitant therapy or the temporal relationship of the event to the IMP will be considered.

### Procedures for reporting serious adverse events

At the occurrence of a patient event that fulfils one or more seriousness criteria, the Investigator must **immediately** forward to AUB/IRB-related site and Merck KGaA, duly completed “SERIOUS ADVERSE EVENT FORM”, even if the data are incomplete, but as soon as the following minimum information is available:

1. Identification of the notifying person
2. Identification of the clinical study
3. Identification of a patient (patient number and/or initials)
4. Description of the SAE and causality

A template of the “SERIOUS ADVERSE EVENT FORM” is provided in Appendix 20.3.

Where needed, the Investigator will ensure a follow-up of an initial SAE to elucidate the nature, the outcome or the causality of the SAE. This may include additional lab tests, histo-pathological examinations, and consultations with other healthcare professionals or any post-mortem findings. This follow-up information should be provided to AUB/IRB-related site and Merck KGaA 24 working hours as of their availability.

TIME FRAME

| **“Initial” SAE**  form | **“Follow-up” SAE**  form |
| --- | --- |
| **IMMEDIATELY**  As of awareness of an SAE occurrence | **24 working hours**  As of availability of essential follow-up information |

FAX OR EMAIL TRANSMISSION

The “SERIOUS ADVERSE EVENT FORM” should be sent to:

**Fax:** +961 1 738 025 (AUB) / +49 6151 72 6914 (Merck KGaA)

Or

**Email:** irb@aub.edu.lb / ICSR_GDS@merckgroup.com

Specifying:

PROTOCOL Number and/or Title

Merck assigned Study Number

SUBJECT Number

SITE Number/PI Name

SAE/ONSET DATE

In rare circumstances, when fax transmission is not possible, reporting by telephone is acceptable. But this should be followed with a completed “SERIOUS ADVERSE EVENT FORM” signed and faxed by the Investigator as soon as possible.

### Medical contacts

For urgent medical and clinical matters or questions, the Investigator may contact the Principal Investigator:

**Name:** Dr Ali Shamseddine, MD, FRCP

**Location:** Department of Internal Medicine, Division of Hematology/Oncology, American University of Beirut Medical Center, Beirut PO Box 11-0236, Lebanon

**Tel nr:** +961 1 355 500 (Ext.: 5390)

**Fax nr:** +961 1 738 025

**Email:** as04@aub.edu.lb

For medical information on the IMP, the Investigator may contact Merck Medical Information portal:

**Name:** Dr. Neyra Manoubi

**Position:** Medical Affairs Director, Merck Serono - Near East region

**Location:** Merck Serono Middle East FZ-LLC - DIFC - Central Park Towers, 5th floor - Gate Boulevard - P.O. Box 22730 - Dubai, UAE

**Tel nr:** +971 443 308 00

**Mobile nr:** +971 529 520 834

**Fax nr:** +971 442 913 90

**Email:** [neyra.mannoubi@merckgroup.com](mailto:neyra.mannoubi@merckgroup.com)

**Website:** [www.merckgroup.com](http://www.merckgroup.com/)

### Follow-up of adverse events

During the study, all AEs and SAEs must be followed up during the study period until resolution or stabilization.

After the end of the study, or at patient's premature discontinuation: Only ongoing AEs or SAEs related to the study IMP, or to non-IMPs required by the study protocol, that are still not “resolved” must be followed-up until they are resolved (with or without sequelae), or until the condition is stabilized or until the Investigator no longer estimates it is clinically significant in a written justification.

If no follow-up information can be provided, the Investigator must:

- Provide a written justification and
- Document the outcome as “unknown” (except for cases where the outcome is known, such as death)

## Pregnancy

Pregnancy is an exclusion criterion.

Highly effective contraception should be applied for both male and female subjects throughout the study and for at least 30 days after last avelumab treatment administration if the risk of conception exists.

However, any occurrence of pregnancy during the study period should be documented by the Investigator on the “PREGNANCY FORM” and faxed or emailed to AUB/IRB-related site and Merck KGaA. If a pregnancy occurs during the study period, the patient should be withdrawn from the study as soon as pregnancy is known and intake of the IMP should be discontinued immediately.

The Investigator should:

- Collect the name and the contact of the physician/obstetrician following-up the patient’s pregnancy
- Complete the “PREGNANCY FORM” and/or the “PARENT-CHILD/FETUS AE FORM”
- And fax or email it to AUB/IRB-related site and Merck KGaA as soon as possible.

A template of the “PREGNANCY FORM” is provided in Appendix 20. 4 and a template of the “PARENT-CHILD/FETUS AE FORM” is provided in Appendix 20.5.

FAX OR EMAIL TRANSMISSION

The “PREGNANCY FORM” and/or the “PARENT-CHILD/FETUS AE FORM” should be sent to:

**Fax:** +961 1 738 025 (AUB) / +49 6151 72 6914 (Merck KGaA)

Or

**Email:** irb@aub.edu.lb / ICSR_GDS@merckgroup.com

Specifying:

PROTOCOL Number and/or Title

Merck assigned Study Number

SUBJECT Number

SITE Number/PI Name

SAE/ONSET DATE

The progression of the pregnancy will be followed up by the Principal Investigator in collaboration with the Investigator or the patient physician and/or obstetrician, and the health of both the mother and newborn will be documented.

## Regulatory safety requirements

The decision to notify SAEs to Competent Authorities (CA)/Ethics Committee is under the responsibility of the Principal Investigator.

The Principal Investigator will ensure that the Investigators, and all other appropriate persons, are informed in a timely manner of findings that could adversely affect the safety of patients.

The Principal Investigator will communicate additional safety information to the appropriate Health Authorities/Ethics Committee and all Investigators, as it becomes available.

Whenever local regulations request from the Investigator to directly notify local Authorities and/or Ethics Committee, the Investigator should provide the Principal Investigator or its representatives with evidence of such notification.

# PARAMETERS AND ASSESSMENT CRITERIA

## Assessment of efficacy

The primary efficacy endpoint is the proportion of patients who achieve a pathological complete response, defined as no viable tumor cells on the resected specimen.

The secondary efficacy endpoints are:

1. PFS at 3 years will be estimated with the Kaplan-Meier method and presented with the 95% CI.
2. Evaluation of response by obtaining TRG just after surgery (week 16 or 17 ± 3 days).

## Assessment of safety

Secondary safety endpoints are the frequency, severity, and attribution of adverse events related to avelumab in a neoadjuvant setting.

AEs and SAEs from all patients followed throughout the study will be recorded and reported, as described in Section 9.5, whether or not the AE was determined to be related to the administration of IMP.

Safety evaluation will include the monitoring of the following items:

- - 1. Clinical assessments

| **Clinical assessment** | **Time of assessment** |
| --- | --- |
| Routine monitoring of vital signs (heart rate, blood pressure, and body temperature) | At inclusion  Every 2 weeks± 3 days during chemotherapy  Every 3 months after surgery for 3 years |
| Weight |
| Physical examinations: total body examination (i.e., general appearance, skin, neck, including thyroid, eyes, ears, nose, throat, lungs, heart, abdomen, back, lymph nodes and extremities) and a clinical neurological examination |
| Pelvic MRI (rectal protocol) | At inclusion  Prior to surgery: pelvic MRI (rectal protocol), optional |
| CT or PET scan of the chest and the abdomen | At inclusion |

- - 1. Laboratory assessment:

| **Blood assessments** | **Time of assessment** |
| --- | --- |
| CBC, BUN, creatinine, electrolytes, SGPT, SGOT, GGT, alkaline phosphatase, bilirubin | Prior to study initiation  Start of chemotherapy  Then every two weeks ± 3 days  Then prior to surgery |
| Free T4 and TSH | At inclusion  At least every 8 weeks during treatment  At end of treatment or 30 days post-treatment safety follow-up |
| Virology | At inclusion |

- - 1. Urine or serum pregnancy test:
       - For women of childbearing potential and who are sexually active must be performed at baseline and least every month during treatment.
       - It should be negative when the patient is enrolled in the study.

Significant findings of any safety evaluation must be recorded either on the Relevant Medical History/Current Medical Conditions eCRF (if present before signing informed consent) or on the AEs eCRF (if newly occurring or worsening since signing informed consent).

## Exploratory endpoints

The exploratory endpoints are:

1. Evaluation of biomarkers CD4+, CD8+ and CD3+ T cell infiltration, and changes in PD-L1 expression. This endpoint aims to demonstrate proof of concept that short-course radiation therapy up-regulates PD-L1 expression and induces immune cell infiltration.
2. Serum markers of inflammation: neutrophil/lymphocyte ratio.
3. Quality of life assessed using FACT-C questionnaire (FACT-C, English version 4 of 16 November 2007 or Arabic version 4 of 03 September 2014).

# DATA MANAGEMENT

## eCRF completion

### Introduction

All of the information required to be reported to the Principal Investigator as per protocol will be entered in the eCRF for each study patient. The eCRF must be completed by the Investigator or any study center staff designated by the Investigator. The Investigator is responsible for ensuring that data recorded in the eCRF are complete, accurate and legible. The eCRF should be completed before review by the CRA.

### General instructions

- - - 1. ***Overview***

The CRFs are electronic. CRFs should be completed in English.

The patient-reported outcome (FACT-C) should be completed by the patient in Arabic, or in English if the patient does not understand Arabic. Once completed, the site staff will do the data entry in the eCRF.

All data in the eCRF must come from and be consistent with the source documents, i.e. patient’s file or medical records.

Any discrepancy between the data in the eCRF and those in the source documents should be corrected by the Investigator.

Abbreviations will be avoided since they are often ambiguous.

- - - 1. ***Header information***

Header information should include at least:

The protocol number,

The name/number of the visit,

The date of the visit,

Patient identification number: patient number is recorded with the center number provided by the CRA at the set-up visit. The patient number is a sequential number incremented with each patient included in the order of signature of informed consent.

- - - 1. ***Recording dates and time***

Dates will be recorded in the eCRF using two digits for the day and the month and four digits for the year.

Example: February 28, 2014 must be entered as 28/02/2014.

Time will be recorded using four digits according to the 24-hour clock.

Example: 3:25 pm must be entered as 15:25.

- - - 1. ***Recording of missing data***

The Investigator should make every effort to provide the information required. Use of the following codes should be kept to a minimum.

The Investigator or designated person should enter in the eCRF:

UK: If the requested item is unknown or not available

NA: If the requested item is not applicable

ND: If the requested item was not done

- - - 1. ***eCRF corrections***

Discrepancies may be identified by the data management system automatically at entry or after entry via rules defining acceptable data. They can also by manually identified at any point during the review process by CRAs or Data Manager. Identified discrepancies will be shown as queries on eCRF and will need to be resolved by the Investigator or designated person.

All corrections to an original eCRF entry must indicate the reason for change. The Investigator is required to sign the eCRF after all data have been captured for each patient. If corrections are made after review and signature by the Investigator, he or she must be made aware of the changes, and his or her awareness documented by re-signing the eCRF.

- - - 1. ***Confidentiality***

Patient names must be kept confidential and should not appear on any eCRF page or study-specific documents provided to the CRO. The CRAs or other agents of the sponsor shall not access the site’s medical records unaccompanied by the site’s medical staff. Also, no personal health information is recorded by the CRA except as authorized by the IRB approved protocol; the IRB approved signed subject consent form and the signed clinical trial agreement.

- - - 1. ***eCRF Completion in case of premature withdrawal***

An eCRF must be completed for all patients with a signed informed consent up. Premature withdrawal must be documented on the end of study form of the eCRF with the cause having led to premature withdrawal.

### Specific Case Report Form instructions

Specific instructions for completing the eCRF will be detailed in a specific document “eCRF completion guidelines”.

## CRF and data handling

Data processing, from data collection to database lock, will be carried out in accordance with GCP (see ICH-E6, Section 5).

The database structure, data entry manual, coding rules and computerized validation, are defined in a Data Management Plan.

The database and data entry screens will be created in software specifically designed for clinical data management in compliance with ICH-E6 requirements.

All eCRF received in the Data Management Unit will be tracked by the Data Manager. The consistency of data will be checked by computerized programs and related queries will be generated for resolution by the Investigator. The database will then be updated accordingly.

Coding of medical terms and drug names, quality controls to ensure the overall quality and consistency of the database and reconciliation of SAE reports with the pharmacovigilance database will also be carried out.

At the end of the data handling process, a data review meeting will be held to prepare the database lock. After database lock, data will be transferred into a statistical software for the production of statistical analyses.

# STATISTICS

## Statistical analysis plan

The detailed technical aspects of the statistical analyses will be provided in the SAP. The SAP will possibly take protocol amendments into account and adapt to unexpected issues raised by the trial running and/or data that affect planned analyses in the protocol.

Any deviations from the protocol regarding the description of statistical analysis in the SAP will be discussed in the study report.

Prior to locking the database, a data review meeting will be planned to review individual data and validate the SAP.

The distribution of parameters, including safety data, will be summarized using descriptive statistics according to the study variable:

- Categorical variables (binary, nominal and ordinal) will be presented by contingency tables (frequencies and percentages). Number and percentages of missing data will also be mentioned.
- Quantitative variables will be presented by their mean, standard deviation, standard error, median, quartiles and range, minimum and maximum values. The number of documented values will also be mentioned.

The primary endpoint will be presented by number and proportion of patients who achieved pCR along with the one-sided 95% confidence interval. Using Kaplan-Meier method, the median PFS will be estimated along with its 95% confidence interval. Exploratory variables will be analyzed according to their scale of measurement by using mean ± standard deviation or frequency distribution for numeric and categorical variables respectively.

Frequency distribution for AEs and SAEs will be presented per cycle and per patient. Similarly this will be done for the SAE of grade 3 or above combined.

No subgroup analysis is planned.

## Sample size determination

Assuming the historical rate of pCR with short radiation followed by systemic 5FU-oxaliplatin chemotherapy is 16% to 19% (Bujko et al., 2016a) and the rate of pCR with the addition of avelumab increases to 35%, Simon’s two-stage design (Simon R, 1989) will be used. The null hypothesis that the true response rate is ≤16% will be tested against a one-sided alternative. In the first stage, 13 patients eligible for the primary efficacy analysis will be accrued. If there are 2 or fewer patients with a pCR in these 13 patients (2 patients out of 13 constitutes 15%), the study will be stopped. Otherwise, 23 additional (eligible) patients will be accrued for a total of 36 patients eligible for the primary efficacy analysis. The null hypothesis will be rejected if at least 10 patients out of 36 have a pCR. This design yields a type 1 error rate of 0.05 and a power of 0.8 when the true pCR rate is 35%. The use of the Simon 2-stage design enables an interim analysis for both efficacy and safety to be performed following treatment of the first 13 (eligible) patients. In order to achieve the eligible 36 patients for the primary efficacy analysis, the study might have to recruit 15 to 20% more patients and this will increase the sample size to 44 patients. This increase in sample size will also benefit the analysis of the secondary outcomes for example by possibly increasing the precision with the estimation of the progression free survival outcome.

## Randomization

Not applicable.

## Protocol deviations and analysis sets

All the deviations from protocol definitions will be listed and defined as major or minor deviations in the SAP.

According to the proposed analysis, the definition of analysis sets is recommended as follows:

- - 1. ITT set: all patients who received at least one administration of the IMP.
    2. Modified ITT Set (MITT): all ITT patients who received at least one administration of IMP, with (the baseline) assessment available and have undergone surgical resection. When further restrictions/qualifications are suggested, they must be specified in a protocol amendment.
    3. Full-Analysis Set (FAS): all patients who received at least one administration of IMP.
    4. Total Treated Set: all patients who received at least one administration of IMP.
    5. Per Protocol (PP) Set: all MITT patients without major deviations. These major deviations must be defined in the protocol or in the SAP.

## General rules for handling of missing or inconsistent data

No replacement of missing data will be performed except for:

- - 1. The primary efficacy endpoint is a dichotomous criterion (yes/no). A missing primary endpoint will be considered as if the endpoint is not fulfilled (no pCR).
    2. The duration would be based on the time difference between the date the patient signs the informed consent and is enrolled in the study and the date of progression of disease on imaging. Otherwise, if the patient’s disease did not progress, the patient will be censored in the analysis and the last follow-up date or date of last communication with the patient will be applied instead.

All estimated or replaced data will be edited along with a flag in the statistical appendices.

## Demographic and baseline characteristics

Demographic and baseline characteristics of all patients in the FAS will be analyzed.

The primary efficacy analysis will be performed on the MITT set.

### Demographic characteristics, medical history and diagnoses

All demographic characteristics and all medical history data will be presented.

### Previous treatments

All previous treatments will be listed by patient with the associated ATC codes for the WHO drug dictionary.

Descriptive statistics will be produced to analyze the previous treatments by therapeutic class as defined in Section 7.1.

### Baseline efficacy variables

Categorical variables (binary, nominal and ordinal) will be summarized by contingency tables (frequencies and percentages),

Quantitative variables will be summarized by their mean, standard deviation, standard error, median, quartiles and range, minimum and maximum values.

### Baseline safety variables

All safety variables will be presented. Baseline data are defined as the last available data before the start of IMP infusion.

All laboratory data except those defined as efficacy variables (in the above section) or as exploratory variables will be defined as safety criteria. All laboratory data will not be displayed in the baseline section but in the analysis of safety in order to display in the same table the baseline and other data.

In addition, the frequency, grade, and attribution of surgical complications to the neoadjuvant treatment will be described using the Clavien-Dindo classification (to grade surgical complications).

## IMP and concomitant medications

### Extent of exposure

Summary tables will display the number of patients exposed, duration of exposure (in minutes, automatically derived from the start and end times of IMP infusion/injection), total volume (mL) and total administered dose (g) to which each patient was exposed.

### Concomitant medications

All concomitant medications will be listed by patient with their ATC codes.

Descriptive statistics will be produced to analyze the Concomitant medications by therapeutic class as defined in Section 7.2.

A particular attention will be paid to:

- - Anti-emetics
  - Oxaliplatin
  - Leucovorin
  - Fluorouracil (infusion)

## Efficacy analysis

### Primary efficacy variable(s)

- - 1. ***Description of the primary efficacy variable***

The primary endpoint is the proportion of patients who achieve a pathologic complete response, defined as no viable tumor cells on the resected specimen, in the MITT set.

The primary efficacy variable is a dichotomous variable (yes/no) that will be summarized using a proportion.

- - - 1. ***Hypothesis Test and Primary Analysis Model***

A Simon’s two-stage optimal design will be applied with the following hypothesis:

- H_0_: p (pCR) ≤0.16
- H_1_: p (pCR) ≥0.35

If 2 or less patients achieved pCR in the first stage with 13 MITT patients, the study will be stopped, otherwise another 23 MITT patients will be added to the study (stage 2) and the results of the 36 MITT patients will be analyzed at the end of the study. If overall 10 or more patients achieved pCR, then the null hypothesis will be rejected with a type I error of 0.05.

In addition, the one-sided 95% confidence interval (Wilson Score method) of the percentage of patients with pCR will be estimated for all patients in the MITT set and the PP set.

- - - 1. ***Multiple comparisons and interim analyses***

Interim analysis will be done upon recruitment of the first 13 MITT patients to assess the futility and efficacy of the study.

### Secondary efficacy variable(s)

#### *Description of the secondary efficacy variable*

The secondary efficacy variable are listed in Section 10.1.

#### *Analysis of secondary efficacy variables*

Mainly, PFS will be estimated using Kaplan Meier methods. Median PFS and the PFS rate at 3 years and corresponding 95% confidence intervals will be presented. PFS will be defined for all patients regardless of their pCR as the time between the date the patient is enrolled in the study and the date the disease progression is shown on imaging or the date of death whatever is earlier and the last follow up date in the absence of disease progression or death. Patients without progression or death will be censored at the last assessment regarding progression. PFS will be analyzed for the ITT, the MITT and the PP set.

## Safety analysis

Safety data will be summarized for all patients who received at least one administration of IMP. AEs and SAEs will be summarized per cycle and overall using frequency tables. Also, the number and percentage of patients with AEs of grades 3 to 5 will be presented.

## Pharmacokinetics, PK/PD, and analysis

Not applicable.

# STUDY REPORT

A clinical study report, will be prepared in accordance with the ICH-E3 guidelines, by the Principal Investigator or subcontractor in collaboration with the Principal Investigator and, if any, with the scientific committee.

Within 1 year after the end of the study, the Principal Investigator will provide the Health Authorities with the full study report or summary. Only the Principal Investigator is entitled to make the study report available to the Authorities.

Neither the complete report nor any part of the study report may be used without the approval of the Principal Investigator.

# CONFIDENTIALITY AND PUBLICATION

## Patient confidentiality

Patient data will be kept strictly confidential and patient anonymity will be protected by using number codes and initials.

The Principal Investigator, Phoenix Clinical Research and the Health Authorities will not disclose any personal patient information.

## Use of information

The Investigator shall not disclose unpublished data or information related to the study provided by the Principal Investigator, including but not limited to the study product characteristics, the IB, the study protocol, CRF/eCRF, assay methods and scientific data, to any third party without written approval from the Principal Investigator.

In addition, any new information that may become available during the course of the study shall be considered as confidential and shall not be used for any purpose other than the performance of the clinical study.

The study data are the property of the Principal Investigator. The co-Principal Investigators and the Investigators and any of the research staff shall obtain written approval from the Principal Investigator prior to the publication/communication of the results of any work carried out during or in relation to the study.

Publication and/or communication of the results of the clinical study is the responsibility of the Principal Investigator. It will be of a cooperative nature involving authors representing the Principal Investigator, the Investigators and the scientific committee, if any.

The Principal Investigator reserves the right to request modification of the content and/or timing of any publication or presentation if a patent application, an existing patent or other proprietary rights may be jeopardized.

Authorship of any publication related to the study and the order of presentation of the authors’ names shall be approved by the Principal Investigator. The Principal Investigator shall not use an Investigator’s name in any publication without his/her written permission and vice versa.

Under all circumstances, the Principal Investigator should revert back to Merck’s approval for publication.

# ARCHIVING

The Investigator must retain all study documents for 15 years or longer if required by specific local requirements. These documents include but are not limited to signed protocol, IB, CRFs, medical records, laboratory reports, informed consent forms, drug disposition records, safety reports, information regarding participants who discontinued, and other relevant documents and data.

The study-related documents should be kept together in the ISF provided to the Investigator by the Principal Investigator.

Sufficient information about the identity of all study patients, e.g. name, medical records number, patient number and study number, should be retained by the Investigator so that any Principal Investigator representatives, auditors or inspectors may access this information when required.

The Investigator will contact the Principal Investigator for authorization prior to the destruction of any study records or in the event of accidental loss or destruction of any of them.

The Investigator will also notify the Principal Investigator should he/she relocate or move the study-related files to a location other than that specified in the Principal Investigator Study Master File.

All records should be kept in a secure area. In the cases of audit or inspection, they should be easily made available.

# RESPONSIBILITIES OF PARTICIPANTS

## Responsibilities of the investigator(s)

The Investigators will conduct the study in accordance with ICH-E6, all applicable laws in the country where the study is conducted and in accordance with this study protocol.

The responsibilities of the Investigators are summarized below but not limited to:

Patient information and consent:

- 1. Prior to undertaking any study-related procedure, it is the responsibility of the Investigator, or a formal designee, to provide each patient with relevant, comprehensive, verbal and written information, including the written information which received approval or a favorable opinion from the IEC/IRB and the Health Authorities.
  2. Signed informed consent must be obtained prior to undertaking any study-related procedure. Obtaining of consent and how it was obtained must be described and documented in the patient’s file.

Adverse events:

- 1. The Investigator is responsible for ensuring adequate safety monitoring and follow-up of the study patients.
  2. The Investigator must report and handle any serious and non-serious AE, whether clinically observed or spontaneously reported by the patient, using concise medical terminology

Data recording:

- 1. It is the Investigator’s responsibility to ensure, on an on-going basis, completion and validation of all case report forms as well as study-related supportive data. The eCRF must be signed by the Investigator. If the Investigator formally delegates completion of the eCRF, the Investigator nevertheless has the final responsibility for signing the eCRF to certify the accuracy and reliability of the data recorded therein.

Record retention:

- 1. To enable inspections and audits from Health Authorities or the Principal Investigator, the Investigator agrees to keep records, including the identity of all participating patients, i.e. sufficient information to link records, all original signed informed consent forms and copies of all CRFs and detailed records of treatment disposition. The Investigator should maintain a site file with all essential documents.

Use of study-related information:

- 1. The Investigator is obligated to provide the Principal Investigator with complete test results and all data derived from the study.
  2. Only the Principal Investigator may make information available to physicians, Health Authorities and/or patients enrolled in the study, except as required by local regulations.

IMP:

- 1. Responsibility for IMP accountability at the study center rests with the Investigator or with the institution, depending on local regulations.

Quality control:

- 1. The Investigator and the relevant personnel should be available during monitoring visits and possible audits or inspections and ensure that sufficient time is devoted to the process.
  2. The Investigator guarantees the Principal Investigator or its representative and appropriate Health Authorities direct access to source documents.

Study discontinuation:

- 1. Should the Health Authorities or the Principal Investigator decide to discontinue the study prematurely for any reason, the Investigator must promptly, i.e. within 7 days, contact all participating patients so they can be appropriately followed-up. All study supplies must be collected and all case report forms must be completed as fully as possible.

Delegation of Investigator duties:

- 1. The Investigator can delegate tasks to the study team but he/she remains ultimate responsible for the study conduct. The Investigator should maintain a list of appropriately qualified persons to whom significant study-related duties will be delegated.
  2. The Investigator should ensure that all persons assisting with the study are adequately qualified, and are informed about the study protocol, any amendments to the protocol, the study treatments, and their study-related duties and functions.
  3. The Investigator, as well as the study team should supply an up-to-date curriculum vitae (CV) in English.

Study agreement discontinuation:

- 1. During the study, if events such as retirement, promotion or relocation prevent the Investigator from conducting the study as agreed, the Investigator should appropriately transfer his/her responsibilities, knowledge and documents to another willing individual, with the agreement of the Principal Investigator. Study specific contracts must be signed between the Principal Investigator and the newly assigned person.

## Responsibilities of the CRA

The responsibilities of the study CRA are defined in ICH-E6, Chapter 5. The CRA, who is mandated by the Principal Investigator, must ensure that the study is conducted in accordance with Good Clinical Practice guidelines and all applicable local laws, and that the rights, the security and the well-being of the patients are respected.

Communication:

- 1. The CRA is the main line of communication between the Investigator and the Principal Investigator.

Compliance:

- 1. During periodic monitoring visits at mutually convenient times, the CRA has the responsibility of assessing the progress of the study, of checking that the informed consent forms have been signed, of IMP accountability, of ensuring compliance with the study protocol (including storage conditions), and of ensuring the accuracy and completeness of the eCRF. Inconsistencies in the study records are to be resolved.

Source data verification:

- 1. The CRA will perform 100% source data verification and validation and request clarification to ensure the accuracy, completeness and reliability of data.

IMP:

- 1. The CRA must ensure that IMP handling is properly carried out and documented.

ISF:

- 1. The CRA must ensure that the ISF is accurately completed and up-to-date.

# ETHICS AND REGULATORY CONSIDERATIONS

The current study is to be conducted in accordance with globally accepted standards of Good Clinical Practice (ICH-E6) and the revised version of the Declaration of Helsinki (World Medical Association, 2013) as well as with applicable local requirements*.*

The protocol will be submitted to CA and EC/IRB for formal approval of the study conduct in accordance with local regulations.

The study should not begin until the protocol has received applicable approval(s).

In accordance with specific local requirements, the Investigator may be responsible for submitting the protocol and any amendments to the local EC/IRB and CA. A copy of the decision letter, a list and versions of documents submitted the list of EC/IRB members and compliance to regulations should be provided by the Investigator to the Principal Investigator.

During the study, the Principal Investigator should promptly notify the Investigators, CA and EC/IRB of any relevant information that could affect the safety of patients and could impact on the conduct of the study.

**Insurance**

The American University of Beirut will contract civil liability insurance to provide patients with compensation for any injury, including the consequences of administration of the IMP and of the study procedures.

In case of injury or disability resulting from participation in the study, the patient is requested to promptly inform the Investigator.

**Indemnity**

Participation in this study will not entail any financial compensation to patients. Only-trial specific procedures will be covered by the Principal Investigator while the standard of care will be paid by the patient.

Trial-specific procedures:

1. Pre-treatment and on-treatment thyroid function tests
2. Pre-treatment and on-treatment pregnancy test
3. Virology testing
4. Experimental pathology assessment (day 10 post-radiation): sigmoidoscopy + biopsy + pathology analysis
5. Avelumab administration

Standard of care:

1. Pre-treatment imaging: MRI/endoscopic ultrasound/CT or PET-CT scan
2. Pre-treatment biopsy
3. Pre-treatment laboratory tests: CBC, biochemistry, tumor markers (CEA, CA 19-9)
4. Radiotherapy
5. mFOLFOX-6 chemotherapy with supportive anti-emetics and insertion of vascular access device
6. Surgical TME (or abdominoperineal resection if TME is not possible)
7. Follow-up laboratory tests and imaging

**Changes to the protocol**

The Principal Investigator must assume all responsibility and liability resulting from implementation of unapproved deviations or changes.

The only circumstance in which an amendment may be initiated prior to approval by the Health Authorities is where the change is necessary to eliminate immediate hazards to the patients. In this event, the Investigator must notify the Principal Investigator and the Ethics Committee, in writing within 5 working days after implementation.

# AUDIT AND INSPECTION

An audit/inspection may be carried out by qualified Principal Investigator staff, by subcontracted auditors or by representatives of national or foreign Health Authorities to ensure that the study is conducted as per protocol and in accordance with regulatory requirements, and to ensure the validity of the data.

Participation in this study implies acceptance to cooperate in any potential audit/inspection.

The audit/inspection may consist of an inspection of the premises and equipment together with verification of the study documents and data.

The investigational team must be available for inspection or audit.

Audits/inspection may take place after the end of the study.

# REFERENCES

Benson AB et al. NCCN Guidelines Version 2.2016 Panel Members Rectal Cancer. Version 2.2016 [Online] Available from: https://www.tri-kobe.org/nccn/guideline/colorectal/english/rectal.pdf (Last accessed: 04 July 2017)

Boyerinas B et al. Antibody-dependent cellular cytotoxicity activity of a novel anti-PD-L1 antibody avelumab (MSB0010718C) on human tumor cells. Cancer Immunol Res. 2015; 3:1148-57.

[Breugom AJ](https://www.ncbi.nlm.nih.gov/pubmed/?term=Breugom AJ%5BAuthor%5D&cauthor=true&cauthor_uid=25589192) et al. Adjuvant chemotherapy after preoperative (chemo)radiotherapy and surgery for patients with rectal cancer: a systematic review and meta-analysis of individual patient data. [Lancet Oncol.](https://www.ncbi.nlm.nih.gov/pubmed/25589192) 2015; 16(2):200-7.

Bujko K et al., on behalf of the Polish Colorectal Study Group. Neoadjuvant chemoradiation for fixed cT3 or cT4 rectal cancer: Results of a Polish II multicentre phase III study. J Clin Oncol. 34; 2016a (suppl 4S; abstr 489).

Bujko K et al. Long-course oxaliplatin-based preoperative chemoradiation versus 5 × 5 Gy and consolidation chemotherapy for cT4 or fixed cT3 rectal cancer: results of a randomized phase III study. Ann Oncol. 2016; 27(5):834-42.

Chen L et al. Anti-PD-1/PD-L1 therapy of human cancer: past, present, and future. J Clin Invest. 2015; 125:3384-91.

College of American Pathologists (CAP). Protocol for the examination of specimens from patients with tumors of soft tissue. Based on AJCC/UICC TNM, 7th edition. Protocol web posting date: October 2013. [Online] Available from: http://www.cap.org/ShowProperty?nodePath=/UCMCon/Contribution%20Folders/WebContent/pdf/softtissue-13protocol-3120.pdf (Last accessed: 22 June 2017)

[Curtis NJ](https://www.ncbi.nlm.nih.gov/pubmed/?term=Curtis NJ%5BAuthor%5D&cauthor=true&cauthor_uid=28470365) et al. Objective assessment of minimally invasive total mesorectal excision performance: a systematic review. [Tech Coloproctol.](https://www.ncbi.nlm.nih.gov/pubmed/28470365) 2017; 21(4):259-68.

de Castro et al. Impact on overall survival and disease-free survival of adjuvant chemotherapy after neoadjuvant chemoradiotherapy for rectal cancer. DOI: 10.1200/jco.2012.30.15_suppl.e14173. Journal of Clinical Oncology 30, no. 15_suppl - published online before print.

FACT-C. Functional Assessment of Cancer Therapy – For patients with Colorectal cancer. Version 4 of 16 November 2007. [Online] Available from: <http://www.facit.org/facitorg/questionnaires> (Last accessed: 23 June 2017)

Garcia-Aguilar J et al. Effect of adding mFOLFOX6 after neoadjuvant chemoradiation in locally advanced rectal cancer: a multicentre, phase 2 trial. [Lancet Oncol.](https://www.ncbi.nlm.nih.gov/pubmed/26187751) 2015; 16(8):957-66.

Heery CR et al Avelumab for metastatic or locally advanced previously treated solid tumours (JAVELIN Solid Tumor): a phase 1a, multicohort, dose-escalation trial. The Lancet Oncol. 2017; 18(5):587-98.

Highlights of Prescribing Information. BAVENCIO® (avelumab). March 2017. [Online] Available at: <https://www.accessdata.fda.gov/drugsatfda_docs/label/2017/761049s000lbl.pdf> (Last accessed: 19 June 2017)

International Council for Harmonisation of Technical. Requirements for Pharmaceuticals for Human USE (ICH). Integrated Addendum to ICH E6 (R1): Guideline for good clinical practice E6 (R2). Current Step 4 version. Dated 9 November 2016.

Investigator’s brochure of Avelumab. Version 7 of 31 March 2017. Merck KGaA.

Kothari N et al. Management of Locally Advanced Rectal Adenocarcinoma. Hospital Physician Board Review Manual 2015; 11(5):1-7.

[Lynch BM](https://www.ncbi.nlm.nih.gov/pubmed/?term=Lynch BM%5BAuthor%5D&cauthor=true&cauthor_uid=18802160) et al. Prospective relationships of physical activity with quality of life among colorectal cancer survivors. [J Clin Oncol.](https://www.ncbi.nlm.nih.gov/pubmed/18802160) 2008; 26(27):4480-7.

Mass M et al. Long-term outcome in patients with a pathological complete response after chemoradiation for rectal cancer: a pooled analysis of individual patient data. Lancet Oncology 2010; 11(9):835-44.

NCI-CTCAE. U.S. Department of Health and Human Services. Common Terminology Criteria for Adverse Events (CTCAE). VERSION 4.0. Published: May 28, 2009 (v4.03: June 14, 2010). [Online] Available at: <https://evs.nci.nih.gov/ftp1/CTCAE/CTCAE_4.03_2010-06-14_QuickReference_8.5x11.pdf> (Last accessed: 19 June 2017)

Nilsson PJ et al. Short-course radiotherapy followed by neo-adjuvant chemotherapy in locally advanced rectal cancer – the RAPIDO trial. BMC Cancer 2013; 13:279.

Network, N.C.C., NCCN clinical guidelines in oncology - NCCN guidelines: Rectal Cancer. 2016.

Parfitt JR, Driman DK. The total mesorectal excision specimen for rectal cancer: a review of its pathological assessment. J Clin Pathol. 2007; 60(8):849-55.

Sauer R et al. Preoperative versus postoperative chemoradiotherapy for rectal cancer. N Engl J Med 2004; 351:1731-40.

Simon R. Optimal two-stage designs for phase II clinical trials. [Control Clin Trials.](https://www.ncbi.nlm.nih.gov/pubmed/2702835) 1989;10(1):1-10.

[Smith JJ](https://www.ncbi.nlm.nih.gov/pubmed/?term=Smith JJ%5BAuthor%5D&cauthor=true&cauthor_uid=26497495) et al. Organ Preservation in Rectal Adenocarcinoma: a phase II randomized controlled trial evaluating 3-year disease-free survival in patients with locally advanced rectal cancer treated with chemoradiation plus induction or consolidation chemotherapy, and total mesorectal excision or nonoperative management. [BMC Cancer.](https://www.ncbi.nlm.nih.gov/pubmed/26497495) 2015; 15:767.

Thies S, Langer R. Tumor Regression Grading of Gastrointestinal Carcinomas after Neoadjuvant Treatment. Front Oncol. 2013; 3:262.

Topalian SL et al. Targeting the PD-1/B7-H1 (PD-L1) pathway to activate anti-tumor immunity. Curr Opin Immunol. 2012; 24:207-12.

Williams NS et al. The quality of life after rectal excision for low rectal cancer. Br J Surg 1983; 70:460-62.

World Medical Association. Declaration of Helsinki. Ethical Principles for Medical Research Involving Human Subjects. Adopted by the 18th WMA General Assembly Helsinki, Finland, June 1964 and amended by the 29th WMA General Assembly, Tokyo, Japan, October 1975; 35th WMA General Assembly, Venice, Italy, October 1983; 41st WMA General Assembly, Hong Kong, September 1989; 48th WMA General Assembly, Somerset West, Republic of South Africa, October 1996; 52nd WMA General Assembly, Edinburgh, Scotland, October 2000; 53rd WMA General Assembly, Washington DC, USA, October 2002 (Note of Clarification added); 55th WMA General Assembly, Tokyo, Japan, October 2004 (Note of Clarification added); 59th WMA General Assembly, Seoul, Republic of Korea, October 2008; and 64th WMA General Assembly, Fortaleza, Brazil, October 2013.

# APPENDICES

## List of principal investigators and co-principal investigators

| **Principal Investigator** | **Ali Shamseddine, MD, FRCP**  Department of Internal Medicine  Division of Hematology/Oncology  American University of Beirut Medical Center  Beirut P.O. Box 11-0236, Lebanon  Phone : +961 1 355 500 (Ext.: 5390)  Email: as04@aub.edu.lb |
| --- | --- |
| **Co-Principal Investigators** | **Joseph Kattan, MD**  Department of Hematology and Oncology  Hôtel-Dieu de France University Hospital  Beirut, Lebanon, 165191  Phone: +961 1 615 300 (Ext.: 8013)  Email: kattan62@hotmail.com  **Rim Turfa, MD**  Department of Internal Medicine  Division of Hematology/Oncology  King Hussein Cancer Center  P.O. Box 1269, Amman 11941, Jordan  Phone: +962 6 53 00 460 (Ext.: 1656)  Email: [rturfa@khcc.jo](mailto:rturfa@KHCC.JO) |
| **Co-investigators** | **Deborah Mukherji, MD, MRCP**  Department of Internal Medicine  Division of Hematology/Oncology  American University of Beirut Medical Center  Beirut P.O. Box 11-0236, Lebanon  Phone: +961 1 355 500 (Ext.: 7980)  Email: dm25@aub.edu.lb  **Sally Temraz, MD**  Department of Internal Medicine  Division of Hematology/Oncology  American University of Beirut Medical Center  Beirut P.O. Box 11-0236, Lebanon  Phone: +961 1 355 500 (Ext.: 7980)  Email: [st29@aub.edu.lb](mailto:st29@aub.edu.lb)  **Youssef Zeidan, MD, PhD**  Department of Radiation Oncology  American University of Beirut Medical Center  Beirut P.O. Box 11-0236, Lebanon  Phone: +961 1 355 500 (Ext.: 5090)  Email: [yz09@aub.edu.lb](mailto:yz09@aub.edu.lb)  **Ibrahim Khalifeh, MD**  Department of Pathology and Laboratory Medicine  American University of Beirut Medical Center  Beirut P.O. Box 11-0236, Lebanon  Phone: +961 1 355 500 (Ext.: 5175)  Email: [ik08@aub.edu.lb](mailto:ik08@aub.edu.lb)  **Faek Jamali, MD, FACS, FSSO**  Department of Surgery  Division of General Surgery  American University of Beirut Medical Center  Beirut P.O. Box 11-0236, Lebanon  Phone: +961 1 355 500 (Ext.: 5800)  Email: fj03@aub.edu.lb  **Fady Geara, MD, PhD**  Department of Radiation Oncology  American University of Beirut Medical Center  Beirut P.O. Box 11-0236, Lebanon  Phone: +961 1 355 500 (Ext.: 5090-5091)  Email: fg00@aub.edu.lb  **Yasser Shaib, MD, FSCP**  Department of Internal Medicine  Division of Gastroenterology and Hepatology  American University of Beirut Medical Center  Beirut P.O. Box 11-0236, Lebanon  Phone: +961 1 355 500 (Ext.: 5800)  Email: ys22@aub.edu.lb  **Assaad Soweid, MD, FASGE, FACG**  Department of Internal Medicine  Division of Gastroenterology and Hepatology  American University of Beirut Medical Center  Beirut P.O. Box 11-0236, Lebanon  Phone: +961 1 350 000 (Ext: 5341)  Email: as25@aub.edu.lb  **Samer Deeba, MD**  Department of Surgery  Division of General Surgery  American University of Beirut Medical Center  Beirut P.O. Box 11-0236, Lebanon  Phone: +961 1 355 500 (Ext.: 5800)  Email: sd08@aub.edu.lb  **Ghassan Chakhtoura**  **Department of Surgery**  **Hotel Dieu de France University Hospital**  **Beirut, Lebanon**  **Phone: +961 615 300**  **Email: ghassan.chakhtoura@usj.edu.lb**  **Tony Felefly**  **Clement Khoury**  **Issa Mohamad, MD**  Department of Radiation Oncology  King Hussein Cancer Center  P.O. Box 1269, Amman 11941, Jordan  Phone: +962 6 53 00 460  Email: imohamad@khcc.jo  **Faiez Daoud, MD**  Department of Surgery (Surgical Oncology)  King Hussein Cancer Center  P.O. Box 1269, Amman 11941, Jordan  Phone: +962 6 53 00 460  Email: fdaoud@khcc.jo |

## Pharmacy manual – investigator sponsored study use of avelumab

## Guidelines and definitions for reporting (S)AE by the investigator

## (Serious) Adverse Event form

## Pregnancy form

## Parent-child/fetus AE form
